# Bioinformatic Exploration of Metal-Binding Proteome of Zoonotic Pathogen *Orientia tsutsugamushi*

**DOI:** 10.3389/fgene.2019.00797

**Published:** 2019-09-24

**Authors:** Dixit Sharma, Ankita Sharma, Birbal Singh, Shailender Kumar Verma

**Affiliations:** ^1^Centre for Computational Biology and Bioinformatics, School of Life Sciences, Central University of Himachal Pradesh, Kangra, India; ^2^ICAR-Indian Veterinary Research Institute, Regional Station, Palampur, India

**Keywords:** *Orientia tsutsugamushi*, metal-binding proteins, therapeutic targets, structure modeling, bioinformatics

## Abstract

Metal ions are involved in many essential biological processes and are crucial for the survival of all organisms. Identification of metal-binding proteins (MBPs) of human affecting pathogens may provide the blueprint for understanding biological metal usage and their putative roles in pathogenesis. This study is focused on the analysis of MBPs from *Orientia tsutsugamushi* (*Ott*), a causal agent of scrub typhus in humans. A total of 321 proteins were predicted as putative MBPs, based on sequence search and three-dimensional structure analysis. Majority of proteins could bind with magnesium, and the order of metal binding was Mg > Ca > Zn > Mn > Fe > Cd > Ni > Co > Cu, respectively. The predicted MBPs were functionally classified into nine broad classes. Among them, gene expression and regulation, metabolism, cell signaling, and transport classes were dominant. It was noted that the putative MBPs were localized in all subcellular compartments of *Ott*, but majorly found in the cytoplasm. Additionally, it was revealed that out of 321 predicted MBPs 245 proteins were putative bacterial toxins and among them, 98 proteins were nonhomologous to human proteome. Sixty putative MBPs showed the ability to interact with drug or drug-like molecules, which indicate that they may be used as broad-spectrum drug targets. These predicted MBPs from *Ott* could play vital role(s) in various cellular activities and virulence, hence may serve as plausible therapeutic targets to design metal-based drugs to curtail its infection.

## Introduction

*Orientia tsutsugamushi* (*Ott*) is a Gram-negative mite-borne bacterium responsible for life-threatening zoonotic disease, scrub typhus (Walker, 2016; Jain et al., 2018; Jiang et al., 2018). Scrub typhus (mite-borne typhus) represents one of the oldest vector-borne disease which is endemic in Asia Pacific, with some reports from Middle East and South America. One billion people are at risk of attaining the infection and one million people get infected each year globally (Xu et al., 2017). The outbreak of the infection was reported from different Indian provinces and is related to various complications with 30% fatality rate or even higher (Mahajan et al., 2010; Khan et al., 2017; Xu et al., 2017; Jain et al., 2018). There are certain antibiotics for the treatment of disease, but resistance to these antibiotics by strains of *Ott* has been reported and reviewed earlier (Watt et al., 1996; Mathai et al., 2003; Kelly et al., 2017). According to the Centers for Disease Control and Prevention of the United States of America, there is no effective licensed vaccine available for scrub typhus till date.

The ancient bacteria have originated from metal-rich environments, and therefore, metal ions (mostly transition metals) are integral constituents of proteins (Maret, 2016). Metal ions, such as iron (Fe), zinc (Zn), manganese (Mn), and copper (Cu) are engaged in vital biological processes and are crucial for the survival of the microorganisms. Approximately 45% of the proteins require metal ion as a cofactor for their functioning (Klein and Lewinson, 2011). The bacterial pathogens have the ability to sense metal ions, which is important to invade the host tissue and cause disease. The bacterial metalloproteases may disrupt the important host physiological processes such as destructing key signaling intermediates, breakdown of barriers, and release of metals from the host metalloproteins (Porcheron et al., 2013; Ma et al., 2015).

The coevolution of pathogens and host for the desire of metal ions as an indispensable component of cellular metabolism revealed emerging paradigms in the field of microbiology, rapid evolution, and metal homeostasis (Palmer and Skaar, 2016). Therefore, to acquire a deep understanding of metal homeostasis mechanisms of the pathogens, we first need detailed knowledge of their metal-binding proteins (MBPs). The computational biology and bioinformatics have emerged as highly promising, rapid and efficient approaches for extracting information of genes and proteins from the available sequence data for commercial and therapeutic applications (Singh et al., 2017; Sharma et al., 2018a). In addition, the *in silico* approaches are time saving, less expensive, and can serve as the startup for further experimental studies. We herein present the detailed *in silico* report on metalloproteome of human bacterial pathogen, *Ott* describing its putative MBPs that are probably involved in virulence and may serve as potential targets for the drug discovery process.

## Materials and Methods

### Sequence Data Retrieval

The available complete dataset of protein sequences of *Orientia tsutsugamushi* strain Ikeda (Nakayama et al., 2008) was downloaded from the RefSeq database at the National Centre for Biotechnology Information (ftp://ftp.ncbi.nlm.nih.gov/genomes/all/GCF/000/010/205/GCF_000010205.1_ASM1020v1/GCF_000010205.1_ASM1020v1_protein.faa.gz). RefSeq is the comprehensive, curated, annotated, and nonredundant sequence database of genomes, transcripts, and proteins (Pruitt et al., 2006).

### Identification of Putative MBPs of *Orientia tsutsugamushi*

**Figure 1:**
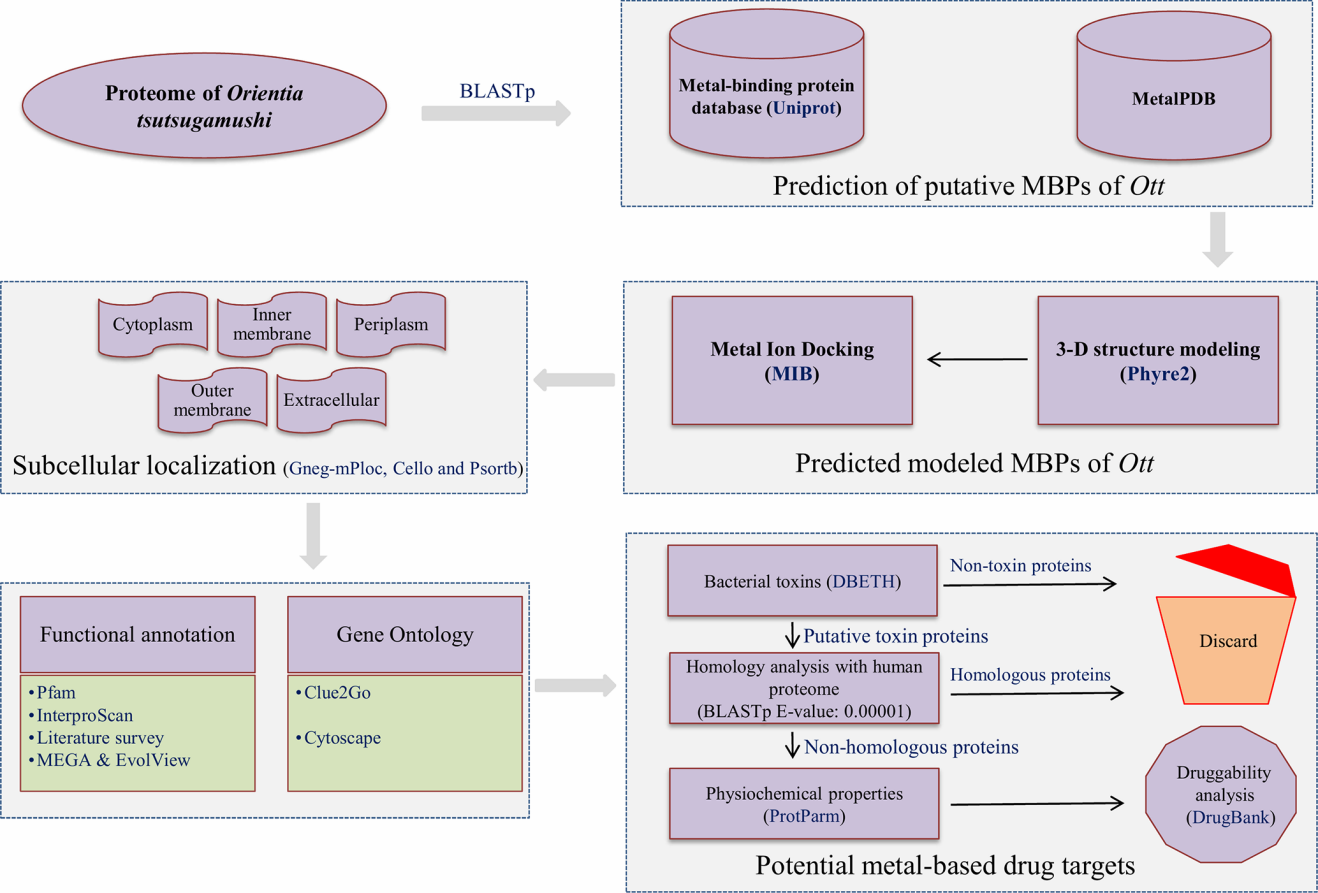
Overall scheme used for the work. The entire proteome of *Orientia tsutsugamushi* (*Ott*) strain Ikeda contains 1,325 protein sequences downloaded from the RefSeq database (Pruitt et al., 2006). The whole protein sequences were investigated for the prediction of metal (Mg, Ca, Zn, Mn, Fe, Cd, Ni, Co, and Cu) binding sequence motifs in two steps. In the first step, local BLASTp (Altschul et al., 1990) search of *Ott* proteome was accomplished with dataset of MBPs that was retrieved from the Uniprot at E-value of 0.00001. In the second step, the resultant proteins from the first step was searched against the dataset of MetalPDB (Putignano et al., 2017) with E-value 0.00001. The 3D-structural modeling of selected putative MBPs was done by Phyre2 program (Kelley et al., 2015), and selected 3D models were analyzed for metal-binding structural motifs using MIB (Lin et al., 2016). The subcellular localization of predicted MBPs was carried out using consensus of bioinformatics server Gneg-mPloc (Shen and Chou, 2010), CELLO (Yu et al., 2004), and PSORTb 3.0.2 (Yu et al., 2010). The functional domain and family characterization was carried out by InterProScan (Jones et al., 2014) and Pfam (Finn et al., 2015) at default parameters. Clustering of the putative MBPs was completed by MEGA v6 (Tamura et al., 2013), and constructed clustergram was visualized by EvolView v2 program (He et al., 2016). The Gene Ontology-based functional (biological and molecular) network were created by ClueGO v2.3.3 (Bindea et al., 2009) and visualized by cytoscape (Shannon et al., 2003). The potential bacterial toxins were predicted by DBETH (Chakraborty et al., 2011) among the putative MBPs, and BLASTp (Altschul et al., 1990) search of selected potential virulent MBPs was done against the host proteome (human; taxid: 9906) at E-value of 0.0001 to exclude homologous proteins and select nonhomologous putative virulent MBPs. The physiochemical properties of selected proteins were calculated with Expasy's ProtParm (Gasteiger et al., 2003). Furthermore, the druggability analysis of nonhomologous putative virulent MBPs was checked by DrugBank 5.0.11(Wishart et al., 2017).

The overall scheme of the work is explained in **Figure 1**. The prediction of MBPs was carried out in two steps. In the first step, the search for MBPs was performed in Uniprot. The keywords (iron binding, zinc binding, calcium binding, magnesium binding, manganese binding, copper binding, cadmium binding, cobalt binding, and nickel binding) were used as inputs to identify proteins which bind to respective metals. The resulting data were retrieved from Uniprot protein knowledgebase (release November 2017). The retrieved data were converted into local database, and standalone BLASTp (Altschul et al., 1990) search for *Ott* proteome was performed with expect value (E-value) 0.00001. This was performed in order to acquire the authentic sequences with considerable similarity to other annotated MBPs in the database. In the second step, the shortlisted proteins from the first step were searched against MetalPDB database with E-value of 0.00001. MetalPDB is the database which offers the information on various metal-binding sites present in three-dimensional (3D) structure of biological macromolecules (Putignano et al., 2017). The proteins which showed homology with desired E-value cutoff (≤0.00001) were nominated as putative MBPs of *Ott*.

### Three-Dimensional Structure Modeling and Metal Docking

3D structure modeling of the putative MBPs of *Ott* was performed by Protein Homology/analogY Recognition Engine v2.0 (Phyre2) program (Kelley et al., 2015). The criteria was set as earlier defined, i.e., confidence score of ≥90% and coverage ≥50% (Sharma et al., 2017). The Phyre2 tool employs the Hidden Markov Model (HMM-HMM) alignment homology approach to predict and shape 3D protein structures. The 3D modeled proteins were further investigated for metal (Fe^2+^, Fe^3+^, Zn^2+^, Cu^+^, Cu^2+^, Mg^2+^, Mn^2+^, Ca^2+^, Cd^2+^, Ni^2+^, and Co^2+^) binding structural motifs with metal ion-binding site prediction and docking server (MIB) (Lin et al., 2016). MIB uses the fragment transformation method to predict the residues which bind to the metal ion within the range of 3.5Å (Lin et al., 2016).

### Subcellular Localization

The subcellular localization of the predicted metalloproteins was carried out by Gneg-mPLoc (Shen and Chou, 2010), CELLO (Yu et al., 2004), and PSORTb 3.0.2 (Yu et al., 2010). Gneg-mPLoc is based on the information of functional domain, Gene Ontology, and sequential evolution. CELLO uses support vector machine classifiers, which are based on numerous n-peptide compositions to predict the subcellular location of the Gram-negative bacterial proteins. PSORTb employs six modules and creates a Bayesian network for predicting the final subcellular localization on the basis of performance of each module. These tools are specific and efficient for the prediction of subcellular location of Gram-negative bacterial proteins, and consensus of these tools was used for final prediction. The overall success rates accomplished by these bioinformatics servers, i.e., Gneg-mPLoc, CELLO, and PSORTb 3.0.2, are 85.5%, 89%, and 98.3%, respectively.

### Functional Classification and Gene Ontology Network Construction of Putative MBPs

The functional annotation of the putative MBPs was done by exploring conserved domains, family and superfamily with the help of InterProScan (Jones et al., 2014) and Pfam (Finn et al., 2015) at default parameters. The literature survey was performed for each predicted MBP based on the identified domain/family, and then, these proteins were further divided into broad functional classes. The predicted MBPs of *Ott* were clustered on the basis of identified domains using MEGA software, ver. 6 (Tamura et al., 2013). The constructed clustergram was further visualized by EvolView v2 program (He et al., 2016). After domain-based functional annotation, the Gene Ontology (GO) functional (biological and molecular) network construction of putative MBPs was executed by ClueGO v2.3.3 (Bindea et al., 2009) plugin of Cytoscape (Shannon et al., 2003). Each GO biological process or GO molecular function term was depicted by node (circle), and the contacts between GO terms (biological or molecular) were represented by edge. ClueGO utilizes kappa score (statistical method) for functional grouping of the GO terms (Huang et al., 2007), and significance of the network was determined by node size.

### Bacterial Toxins Prediction

The bacterial toxins are considered as one of the effective targets for drug development process. We used the Database of Bacterial Exotoxins for Humans (DBETH) (Chakraborty et al., 2011) database for prediction of virulence factors. DBETH database utilizes support vector machine algorithm to predict bacterial toxins of human affecting pathogens. We have first performed the DBETH analysis on the whole proteome of *Ott* strain Ikeda and then extracted putative virulent MBPs among them. This was done in order to check the proportion of putative virulent MBPs among the total putative virulent proteins of *Ott* strain Ikeda.

### Subtractive Proteomic Approach

Furthermore, to avoid cytotoxicity and cross-reactivity of the drug with the host cell, we have adopted the subtractive proteomics approach. Using this approach, the BLASTp search was performed on screened putative virulent MBPs (https://blast.ncbi.nlm.nih.gov/Blast.cgi?PAGE=Proteins) against the host (human; taxid: 9906) proteome at E-value of 0.0001 in order to select the nonhomologous proteins and to exclude homologous proteins. The putative virulent MBPs showing hits at E-value ≤10^−4^ were considered as homologous and were neglected from the further study. The rest of putative virulent nonhomologous proteins were shortlisted for further analysis.

### Druggability Analysis

Furthermore, to select suitable or efficient therapeutic targets the physiochemical characterization and druggability analysis of the shortlisted putative virulent nonhomologous MBPs were performed. The Expasy’s ProtParam (Gasteiger et al., 2003) was used for the physiochemical characterization. The physiochemical parameters [molecular weight, theoretical PI, aliphatic index, instability index, and grand average of the hydropathicity (GRAVY)] estimation provides deep insight into biochemical behavior of the protein, which can serve as the basis for further studies like developing drugs against pathogens. DrugBank Version 5.0.11 (Wishart et al., 2017) was used to evaluate the druggable properties of putative virulent MBPs. The search of predicted virulent nonhomologous MBPs was performed against the DrugBank database at 0.00001 E-value, and other parameters were set as default. DrugBank is the freely accessible comprehensive database containing detailed information about drug targets and drug data. It contains nonredundant protein (i.e., drug targets) sequences which are linked to the drug entries in the database. The protein showing homology with the DrugBank database was appraised as probable druggable proteins or druggable targets.

## Results and Discussion

The report presents *in silico* proteome-wide identification of putative MBPs of human intracellular bacterial pathogen *Ott*. Notably, numerous metal ions are essential for microorganisms and play vital role(s) ranging from acting as a cofactor for enzymes, DNA replication, oxidative stress, structural stability, and bacterial virulence (Porcheron et al., 2013; Ma et al., 2015; Palmer and Skaar, 2016). *Ott* is an intracellular pathogen causing scrub typhus with vast incidents of infections across the globe. In this study, we have integrated various standard computational tools to investigate the putative MBPs in *Ott*. Nearly 24.22% of the total proteins were the putative MBPs, which conform to earlier reports revealing that one quarter of the bacterial proteins require metals for their biological activities (Waldron and Robinson, 2009). The *in silico* mining of MBPs of an organism provides primarily information on size, nature, and functional diversity of its metalloproteome. Furthermore, the putatively identified MBPs probably serve as viable targets for experimental studies, which may enhance our knowledge regarding metal homeostasis mechanism of the particular organism. Earlier, the proteome scale *in silico* approaches has been efficiently implemented for the prediction and characterization of MBPs in plant pathogens (Sharma et al., 2017; Sharma et al., 2018a; Sharma et al., 2019).

Bacteria exploit a variety of metal acquisition and export system to overcome the host defense strategies and to maintain the metal homeostasis by various transcriptional regulators, which help them to adapt the changing environmental conditions (Porcheron et al., 2013). Indeed, to curtail pathogen outgrowth, host employs different defense strategies like metal starvation to bacteria by metal sequestration mechanism (nutritional immunity) and metal toxicity by the release of metal ions in high concentration (Palmer and Skaar, 2016). The necessity of these metal ions during pathogenesis is because of their involvement in various cellular processes. Iron is a vital micronutrient which acts as a cofactor for various enzymes and play important role(s) in various metabolic processes, i.e., energy generation, tricarboxylic acid (TCA) cycle, DNA replication, and protection against oxidative stress (Skaar, 2010; Ma et al., 2015). Bacteria secrete siderophores to acquire iron from the host cell and regulate iron-mediated virulence (Skaar, 2010). Zinc is also a crucial micronutrient involved in structural stability, catalytic activities, and regulation of vital biological processes including mechanisms of virulence such as invasion, formation of biofilm, and adhesion to host cells (Shafeeq et al., 2013). The role of Mg^2+^ (most abundant divalent metal ion in a living cell) is also well known earlier in numerous physiological processes, transport, and virulence of bacteria (Groisman et al., 2013). The function of Ca^2+^ in bacteria is mostly noticed in protein stability, cell signaling, cell cycle, and cell division (Michiels et al., 2002). Other metals such as Cu, Cd, Mn, Co, and Ni integrate with biomolecules such as proteins and play catalytic role(s) in various cellular and biological process ranging from respiration, DNA replication, transcription, and response to oxidative stress (Kim et al., 2008; Porcheron et al., 2013; Palmer and Skaar, 2016).

### Predicted MBPs of *Orientia tsutsugamushi* and Their Metal-Binding Patterns

The standalone BLASTp search of *Ott* whole proteome (1,325 proteins) was accomplished with the dataset of MBPs that was retrieved from the Uniprot. The resultant protein sequences which specify the defined threshold were further selected for second step. In the second step, the selected protein sequences were subjected to MetalPDB search. A total of 605 proteins were shortlisted further, which followed the desired cutoff. It was found that many protein sequences could bind with more than one metal; therefore, the shortlisted 605 proteins were checked for their ability to bind with more than one metal. A total of 345 proteins could bind with one or more than one metal and considered as putative MBPs (**Table S1**).

**Figure 2:**
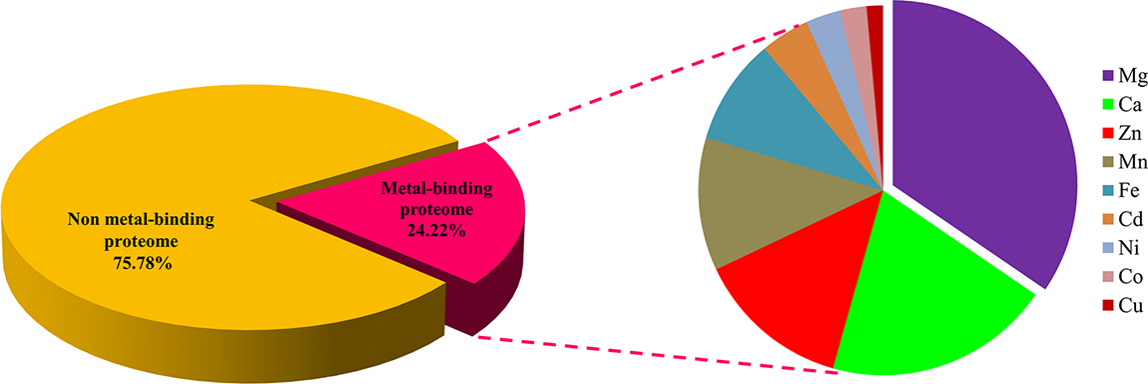
Abundance of MBPs in *Orientia tsutsugamushi* proteome. The abundance of putative MBPs or the size of metalloproteome in the whole proteome of human intracellular bacterial pathogen *Ott* was predicted by sequence and structure-based computational approach. We have predicted nine metals in this study, which are vital for microorganisms to perform diverse role(s) at cellular and biological level including bacterial virulence. Various computational tools were integrated to investigate the putative MBPs in *Ott* proteome. Approximately a quarter (24.22%) of the proteome is putative metal-binding proteome, and most abundant bound metal in the proteome was Mg. The order of metal-binding to proteins was Mg > Ca > Zn > Mn > Fe > Cd > Ni > Co > Cu.

The regions of similarity are located by the alignment between two proteins, which are useful to fetch functional, structural, and evolutionary information (Rost, 1999). Identification of sequence similarity of anonymous protein with the validated proteins of known function submitted in the database enables homology-based annotation (Friedberg, 2006; Singh et al., 2017). In our study, BLASTp search with Uniprot MBPs dataset and MetalPDB database predicted that *Ott* metalloproteome is predominantly rich in Mg followed by Ca, Zn, Mn, Fe, Cd, Ni, Co, and Cu (**Figure 2**). The presence of fewer fractions of Cu-binding proteins correlates towards copper’s efficiency in high redox activity that leads to generation of reactive oxygen species, oxidative stress, and copper toxicity, ultimately damaging the bacterial cell (Andreini et al., 2007b). Therefore, lower concentration of intracellular Cu is needed to be maintained efficiently to prevent toxicity in the cell.

**Figure 3:**
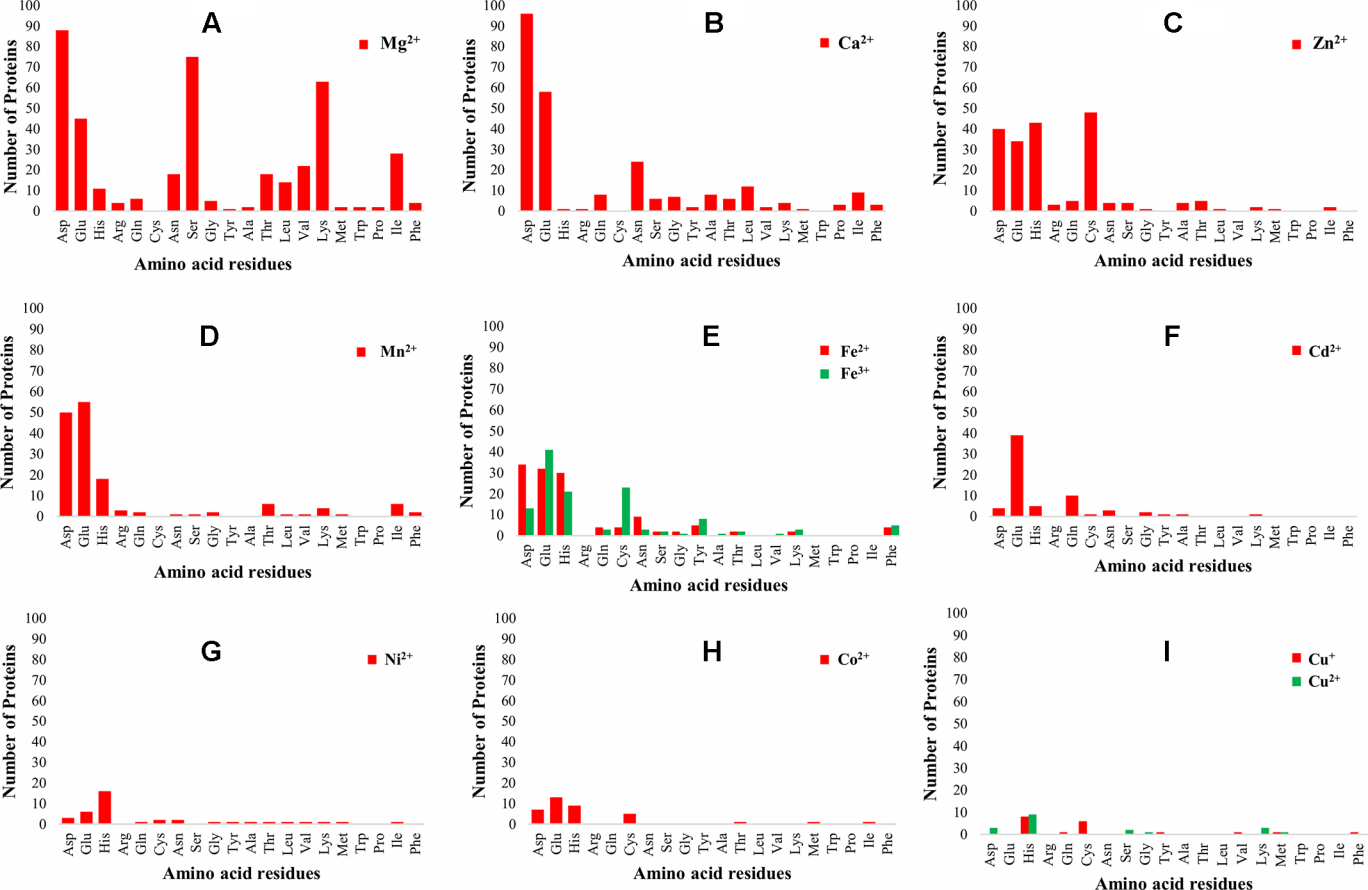
Pattern of amino acid residues present in different metal-binding sites. The graph shows the interaction of amino acid residues with different metal ions (Mg^2+^, Ca^2+^, Zn^2+^, Mn^2+^, Fe^2+^, Fe^3+^, Cd^2+^, Ni^2+^, Co^2+^, Cu^+^, and Cu^2+^). The *X*-axis represents the name of interacting amino acid residues, and the *Y*-axis represents the number of proteins. **(A)** Graph for Mg^2+^, **(B)** graph for Ca^2+^, **(C)** graph for Zn^2+^, **(D)** graph for Mn^2+^, **(E)** graph for Fe^2+^ and Fe^3+^, **(F)** graph for Cd^2+^, **(G)** graph for Ni^2+^, **(H)** graph for Co^2+^, and **(I)** graph for Cu^+^ and Cu^2+^. The most common interacting amino acid residues within binding pocket of the proteins for most abundant metal ion Mg^2+^ are Asp, Ser, Lys, and Glu, whereas for second most abundant metal ion Ca^2+^ are Asp, Glu, and Asn. The favored amino acids for metal ions binding were charged amino acid residues, which were the same as reported earlier, i.e., charged amino acid residues required for coordination of metal ions (Lu et al., 2012; Akcapinar and Sezerman, 2017).

The 3D-structure modeling of the identified 345 putative MBPs was performed by Phyre2. Among these putative MBPs of *Ott*, 325 proteins were modeled by homology modeling method within the specified criteria of query coverage ≥50 and confidence ≥90. The 3D structure of the protein aid in understanding its function and also provides insight into their molecular mechanism (Ilgü et al., 2016). It was investigated with MIB that 321 proteins had structure motifs for binding different metal ions. These 321 proteins were considered as putative MBPs of *Ott*, which had both sequence and structural motifs for binding metal ions (**Table 1**). The obtained putative MBPs showed diversity in their metal-binding sites. The common interacting amino acid residues within binding pocket for Mg^2+^ion was Asp, Ser, Lys, and Glu; for Ca^2+^ was Asp, Glu, and Asn; for Zn^2+^ was Cys, His, Asp, and Glu; for Mn^2+^ was Glu, Asp, and His; for Fe^2+^ was Asp, Glu, and His; for Fe^3+^ was Glu, Cys, His, and Asp; for Cd^2+^ was Glu, Gln, and His; for Ni^2+^ His, Glu, and Asp; for Co^2+^ was Glu, His, Asp, and Cys; for Cu^+^ was His and Cys; and for Cu^2+^ was His, Asp, and Lys. The pattern of interacting amino acid residues with different metal ions are shown in **Figure 3**, and details of interacting residues are listed in **Tables S2A–S2K**. Our findings conform to earlier reports showing that charged amino acid residues are required for the coordination of metal ions (Lu et al., 2012; Akcapinar and Sezerman, 2017).

**Table 1:** Matrix representing Mg-, Ca-, Zn-, Mn-, Fe-, Cd-, Ni-, Co-, and Cu-binding proteins.

Metals	Mg	Ca	Zn	Mn	Fe	Cd	Ni	Co	Cu
Mg	193	71	40	27	22	11	12	8	3
Ca	71	111	23	22	8	13	4	4	2
Zn	40	23	76	16	7	2	2	5	1
Mn	27	22	16	65	6	3	4	7	0
Fe	22	8	7	6	52	4	1	3	2
Cd	11	13	2	3	4	24	4	0	1
Ni	12	4	2	4	1	4	17	1	0
Co	8	4	5	7	3	0	1	13	0
Cu	3	2	1	0	2	1	0	0	8

### Distribution of Putative MBPs in Bacterial Cell

The distribution of MBPs in the subcellular compartments of *Ott* was investigated. Consensus of three prediction programs was used in the study, i.e., Gneg-mPLoc, CELLO, and PSORTb 3.0. The subcellular localization of the 321MBPs of *Ott* showed that 250 were cytoplasmic, 60 were inner membramic, 6 were outer membranic, 3 were periplasmic, and 2 were predicted as extracellular. Prediction of protein subcellular localization is a crucial step in numerous analyses from genome annotation to function prediction (Rey et al., 2005). It can aid in designing an experiment for studying specific protein and can assist in the development of probable vaccines and antimicrobial targets (Paine and Flower, 2002; Mora et al., 2003). Furthermore, precise localization of MBPs also ensure that each metalloprotein get right metal (Waldron and Robinson, 2009)

A considerable amount of MBPs were observed in the cytoplasm, which encompasses three-fourths of the identified MBPs. The highest number of Mg binding (154 proteins) found in cytoplasm, followed by Ca binding (88 proteins), Zn binding (64 proteins), Mn binding (59 proteins), Fe binding (39 proteins), Co binding (13 proteins), equal number of Cd and Ni binding (10 proteins), and Cu binding (5 proteins). It is reviewed earlier that, to meet the cellular metal demand, the cytoplasm should efficiently concentrate metal ions (Ma et al., 2009). Furthermore, the proteome scale subcellular compartmentalization of other intracellular bacteria like *Rickettsia typhi*, *Leptospira interrogans*, and *Mycobacterium tuberculosis* (*M. tuberculosis*) also suggest that most of the proteins localized in cytoplasm (Viratyosin et al., 2008; Sears et al., 2012; Zhu et al., 2015).

In the current study, Mg-binding proteins were found in four subcellular locations, i.e., cytoplasm, inner membrane, outer membrane, and extracellular with a highest fraction (154 Mg-binding sequences) in the cytoplasm. The extensive presence of Mg-binding proteins in cytoplasm might be interpreted either for stabilizing and neutralizing nucleic acids (DNA and RNA) or probably in ribosome assembling (Groisman et al., 2013; Mushegian, 2016). Calcium accounted to be the second abundant metal in the study and the pivotal role of Ca in protein stability, signal transducer in cell cycle, and cell division observed from the existence of 88 Ca-binding proteins present in cytoplasm (Michiels et al., 2002). Zn-binding proteins were found in all the compartments of the bacterial cell, with 64 proteins being in the cell cytoplasm. The ample use of Zn in the cytoplasm of bacteria may be for the regulation of gene expression and for maintaining integrity of the genome (Bertini et al., 2010; Porcheron et al., 2013). Approximate amount of Fe- (11 proteins) and Zn-binding proteins (9 proteins) were detected in the inner membrane. The occurrence of Fe-binding proteins in the inner membrane explains their notable role of electron transport in electron transport chain (Andreini et al., 2007a). Zn-binding proteins in the inner membrane might be involved in import and export of nutrients, metal ions, and toxic substances (Ma et al., 2015). The presence of three Cu-binding proteins in the inner membrane defines their significant role as cofactor of cytochrome c oxidase (Samanovic et al., 2012). A small fraction of MBPs are found to present in the outer membrane, which can help to promote bacterial invasion and transfer of substances across bacterial cell (El-Housseiny et al., 2010; Rollauer et al., 2015). The findings have proclaimed that subcellular localization of predicted MBPs provided the basis for functional information.

### Functional Classification of Putative MBPs

**Figure 4:**
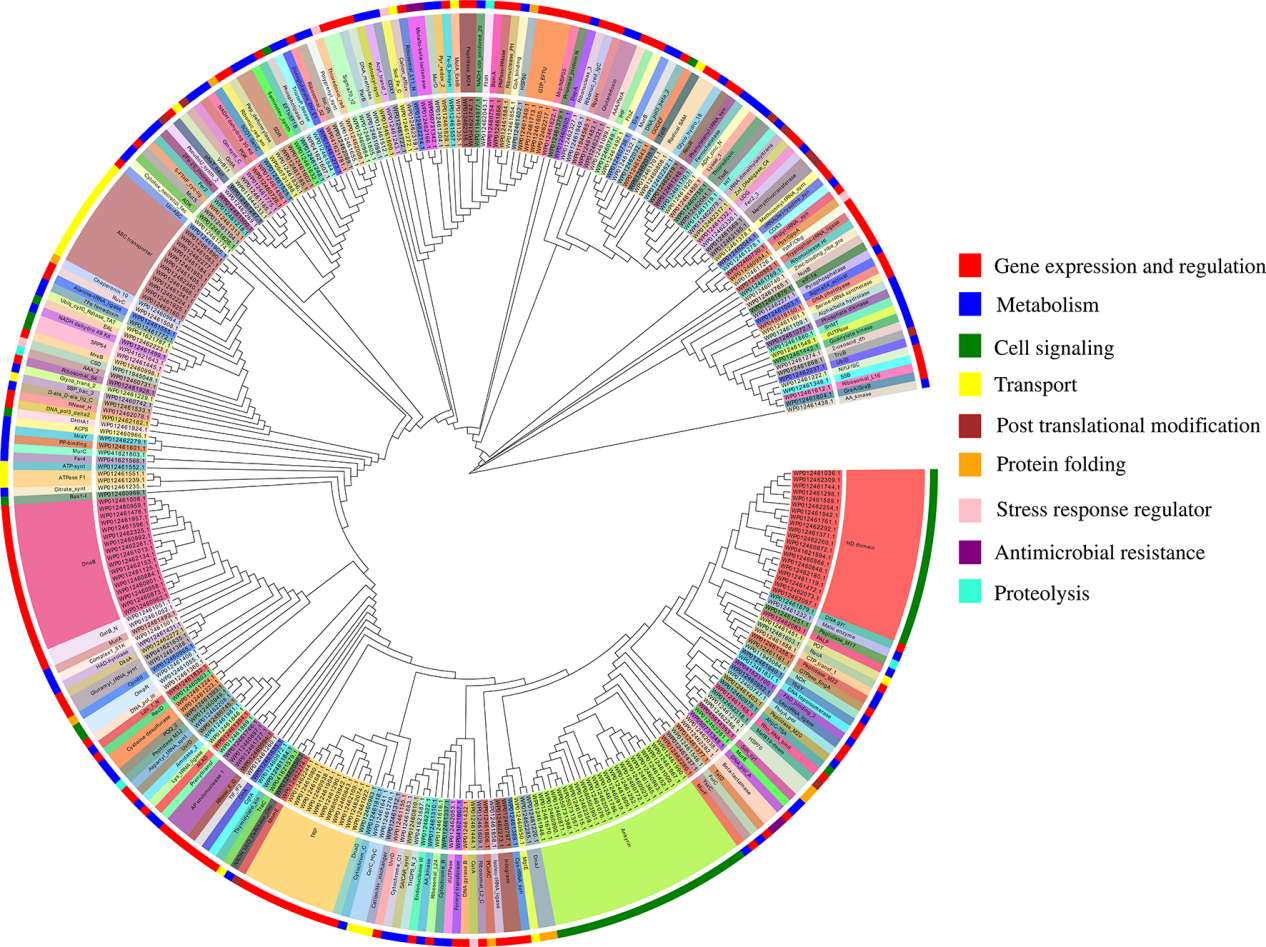
Functional classification of putative 321 MBPs. The predicted 321 MBPs of *Ott* were clustered into 9 broad classes based on their domains or family using MEGA v6 and further designed by EvolView v2. Three circles were shown in the clustergram. The innermost circle shows the sequence identifiers, middle circle represents the functional domains of sequence identifies, and the outermost circle symbolize the broad classification of MBPs based on their role (s) in various biological processes obtained by literature survey. The common domains found in putative 321 MBPs were ankyrin repeat (23), followed by HD domain (20), DnaB-like helicase (17), ABC transporter (12), and tetratricopeptide repeat (11). The nine broad classes include gene expression and regulation, metabolism, cell signaling, transport, posttranslational modification, protein folding, stress response regulator, antimicrobial resistance, and proteolysis (color code of the outermost circle represents each class). The first four categories were predominant in the metalloproteome of *Ott*.

The functional annotation of predicted MBPs showed that proteins containing ankyrin repeat (23), HD domain (20), DnaB-like helicase (17), ABC transporter (12), and tetratricopeptide repeat (TPR) (11) were predominantly present (**Table S3A**). The domain prediction of MBPs and their literature study enables us to identify their function and classify them into nine broad functional classes, i.e., gene expression and regulation (127 proteins), metabolism (84 proteins), cell signaling (53 proteins), transport (26 proteins), posttranslational modification (9 proteins), protein folding (9 proteins), stress response regulator (5 proteins), proteolysis (4 proteins), and antimicrobial resistance (4 proteins) (**Figure 4**, **Table S3A**). Earlier, Cho et al. (2010) used microarray and proteomic approaches to study global gene expression of *Ott* strain Boryong and stated that most of the expressed genes belong to the functional classes protein translation, protein processing or secretion, and DNA repair/replication (Cho et al., 2010). This study also supported our findings as we have also noticed that most of the proteins found in the category of gene expression and regulation.

Furthermore, the whole genome sequencing of the strains of *Ott* revealed that genomes contain repetitive sequences and many of them were pseudogenes (Nakayama et al., 2008; Batty et al., 2018). Ankyrin repeat-containing proteins (Anks), TPR, HD domains, and DnaB helicase are some families of repeats which belong to the category of pseudogenes. In our study, we have noticed the presence of these families, which indicate that metalloproteome of *Ott* also contain pseudogenes. In addition to *Orientia*, other obligate and facultative intracellular bacteria (*Coxiella burnetii*, *Rickettsia* spp., *L*. *pneumophila*, *Wolbahia* *pipientis*, and *Anaplasma* *phagocytophilum*) also contain ankyrin repeats (Voth et al., 2009), and these repeats are functionally diverse. A report on Anks of *Ott* strain Ikeda revealed that Anks resembles with substrates of type-1 secretion system, which inserts traffic to different subcellular localizations or shows a tropism for secretory pathways of the host cell and modulates host cell processes during the infection (VieBrock et al., 2015). Beyer et al. (2015) reported that Anks of *Ott* strain Ikeda have a eukaryotic/Pox-virus F-box motif which recruits or co-opt host cell SCF1 polyubiquitination machinery and exploit host cell ubiquitination (Beyer et al., 2015). Furthermore, regarding metal-binding activity of Anks, it was reported previously that some Anks require cations for their structural and functional stability (Campanacci et al., 2013). Recently, an *in vitro* study on TPR proteins of *Ott* indicate that these binds to DDX3 RNA helicases through N-terminal of DEAD box domain to inhibit eukaryotic translation and to enhance their own replication (Bang et al., 2016). HD domain containing proteins is one of the dominating categories in the metalloproteome of *Orientia*. The finding is supported by earlier studies that HD domain defines metal-dependent phosphohydrolases, i.e., require different metal ions for their catalytic activity (Aravind and Koonin, 1998; Huynh et al., 2015). Furthermore, a report on HD domain phosphodiesterase of intracellular pathogen *Listeria monocytogenes* indicates that this domain is involved in cooperative hydrolysis of c-di-AMP and affects bacterial growth, physiology, and virulence (Huynh et al., 2015). Replicative DnaB helicases also reported in our study, which requires divalent cations for their activity and catalyzes the separation of double-stranded DNA into single-stranded DNA in an ATP-dependent manner (Soni et al., 2003). Our report also enriched with ABC transporters which are conserved across all the organisms (Higgins, 1992). Furthermore, it is documented earlier that microbial ABC transporters are involved in import and export of wide range of substrates (metals ions and their ionic complexes, amino acids, metabolites, sugars, lipids, and antibiotics) and play indispensable roles in their growth, survival, and pathogenesis (Garmory and Titball, 2004; Renesto et al., 2005).

The functional annotation of Zn-binding proteins showed the appearance of ABC transporters, metallo-β-lactamase, zinc finger, and DnaJ domain. The identified domains of Zn-binding proteins have important roles in transporting ions, nutrients and toxic substances, gene regulation, antimicrobial resistance, and protein folding (Wommer et al., 2002; Bertini et al., 2010). The chief domains found in Fe-binding proteins of *Ott* are cytochrome, ABC transporters, ankyrin repeats, glutaredoxin, cysteine desulfurase, and succinate dehydrogenase. These Fe-binding domains may be involved in a variety of functions ranging from metabolism, transport, gene regulation to posttranslational modification (Palmer and Skaar, 2016; Sharma et al., 2018a). The Fe transport and concentration helps in regulating growth and metabolism of Gram-negative bacteria (Kim et al., 2009).

The predicted Mn-binding proteins mainly showed the predominant presence of HD domain, which is primarily involved in signal transduction and metabolism. The role of HD domain has already been discussed in the above paragraph. Predicted 17 Ni-binding proteins mainly comprised of ABC transporters, which may be involved in the transport of toxic substances and metal ions. Earlier, it was reported that Ni transport and metabolism allows immense colonization of *Helicobacter pylori* and *Staphylococcus aureus* (Benoit et al., 2013; Remy et al., 2013). In Co-binding proteins, metallopeptidase (M24 and M20) and ribonucleotide reductase are the prime domains which might help in metabolic process and mediates the synthesis of precursor for DNA replication, respectively (Rawlings and Barrett, 1995; Torrents, 2014). It was also documented previously that Ni- and Co-binding proteins are involved in gene expression regulation and protein metabolism (Sun et al., 2013). The computationally predicted Cu-binding proteins of *Ott* are less in number (0.6% of total proteome) than other predicted MBPs. The results were in accordance with the earlier reports that the fraction of Cu-binding proteins in prokaryotes is <1% (Andreini et al., 2007b). Cytochrome c, CutA, and copper chaperone were the main domains present in identified Cu-binding proteins. The literature survey showed that these Cu-binding proteins play a significant role in metabolic process, stress response, and protein folding (Festa and Thiele, 2011; Palm-Espling et al., 2012).

**Figure 5:**
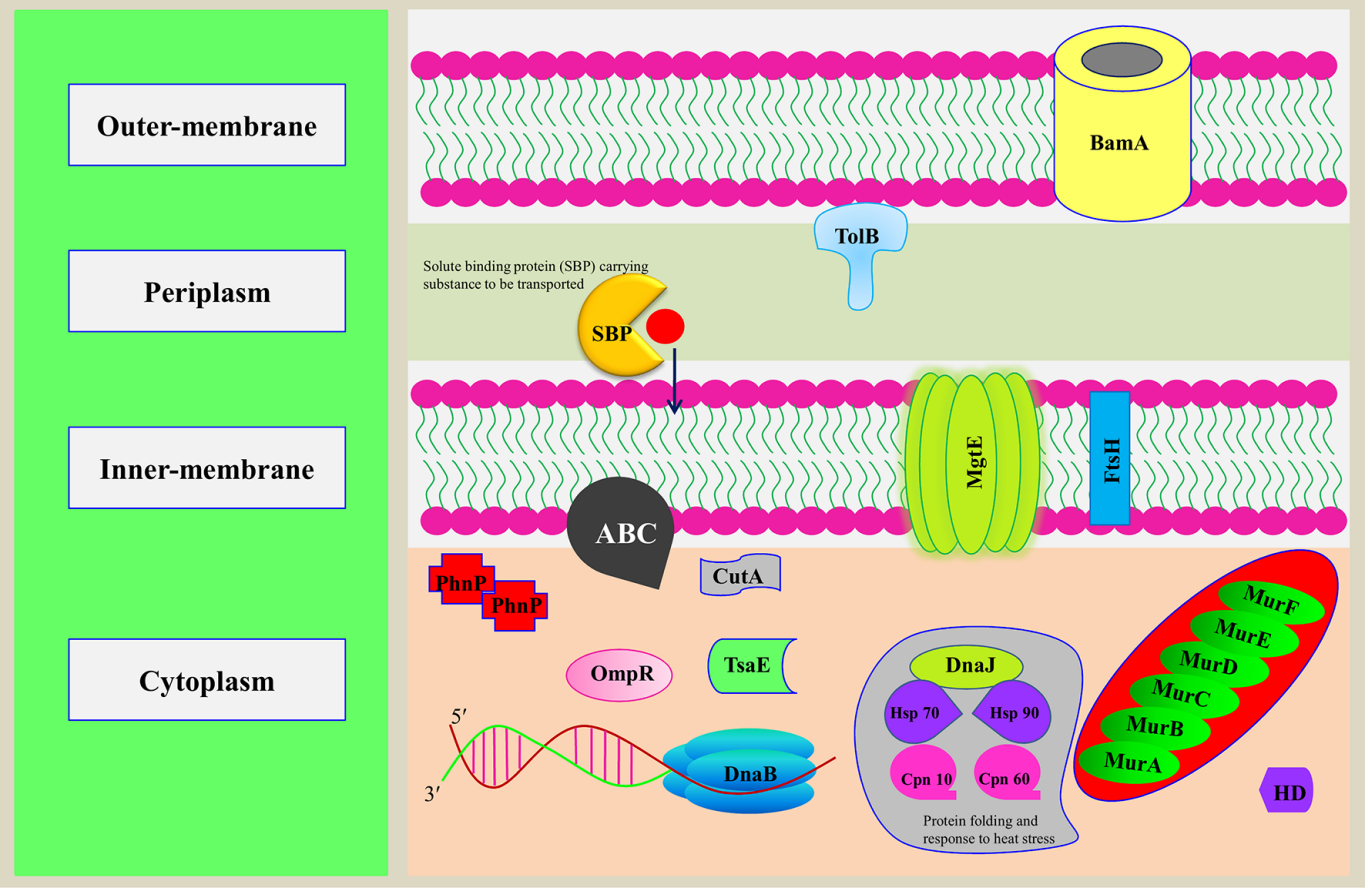
Representation of putative MBPs of *Orientia tsutsugamushi* in bacterial cell. The functional annotation of MBPs was performed after prediction of domains and family by computational tools. In addition to using computational tools, literature review was also performed to classify putative MBPs. Some of the putative MBPs were shown in the bacterial cell with their probable localization and functions they performed. Proteins from each functionally classified category, i.e., metabolism (MurA, MurB, MurC, MurD, MurE, and MurF), gene expression and regulation (DnaB and BamA), transport (ABC, MgtE, TolB, and SBP), cell signaling (HD and OmpR), antimicrobial resistance [metallo-β-lactamase (PhnP)], posttranslational modification (TsaE), protein folding (Hsp 90, HSP 70, Cpn 10, Cpn 60, and DnaJ), proteolysis (FtsH), and stress response regulator (CutA), were shown. MurA, B, C, and D are the major enzymes involved in the biogenesis of the peptidoglycan. DnaB is involved in the separation of DNA duplex into single strands, and BamA is an integral outer-membrane protein engaged in assembly and insertion of β-barrel proteins into the outer membrane. ABC, MgtE, TolB, and SBP are involved in the transport of ions, nutrients, and toxins. HD domain containing proteins involved in cooperative hydrolysis of c-di-AMP and OmpR act as two-component signal transduction transcriptional regulator. PhnP is metallo-β-lactamase which catalyzes the hydrolysis of all β-lactam antibacterials. TsaE helps in tRNA threonylcarbamoyl adenosine modification. FtsH is an ATP-dependent protease which degrades misfolded membrane proteins. CutA exhibit response to ion tolerance. Hsp 90, HSP 70, Cpn 10, and Cpn 60 stabilize and protect the disassembled polypeptides in response to heat shock conditions and are involved in protein folding. DnaJ regulates the activity of Hsp70.

The predicted Cd-binding proteins constitute up to 1.81% of the total proteome, with typically Zn-/Mg-/Ca-/Fe-/Mn-/Ni-binding motifs. The observation is in accordance with the earlier studies that nonessential heavy metal ion Cd^2+^ has the ability to replace native metal ions in metalloproteins which will distort the favored coordination geometry and further lead to functional loss of the protein through notable procedure, known as molecular mimicry (Dudev and Lim, 2003; Chmielowska-Bąk et al., 2013; Friedman, 2014). The Cd-binding proteins mainly have TPR, β-lactamase, and GTPase domain. Previously, it was known that Cd was involved in structural stability of these domains (Concha et al., 1997; Kajander et al., 2007). The overall functional diversity of predicted MBPs of *Ott* is shown in **Figure 5**.

### GO-Enriched Network Construction

**Figure 6:**
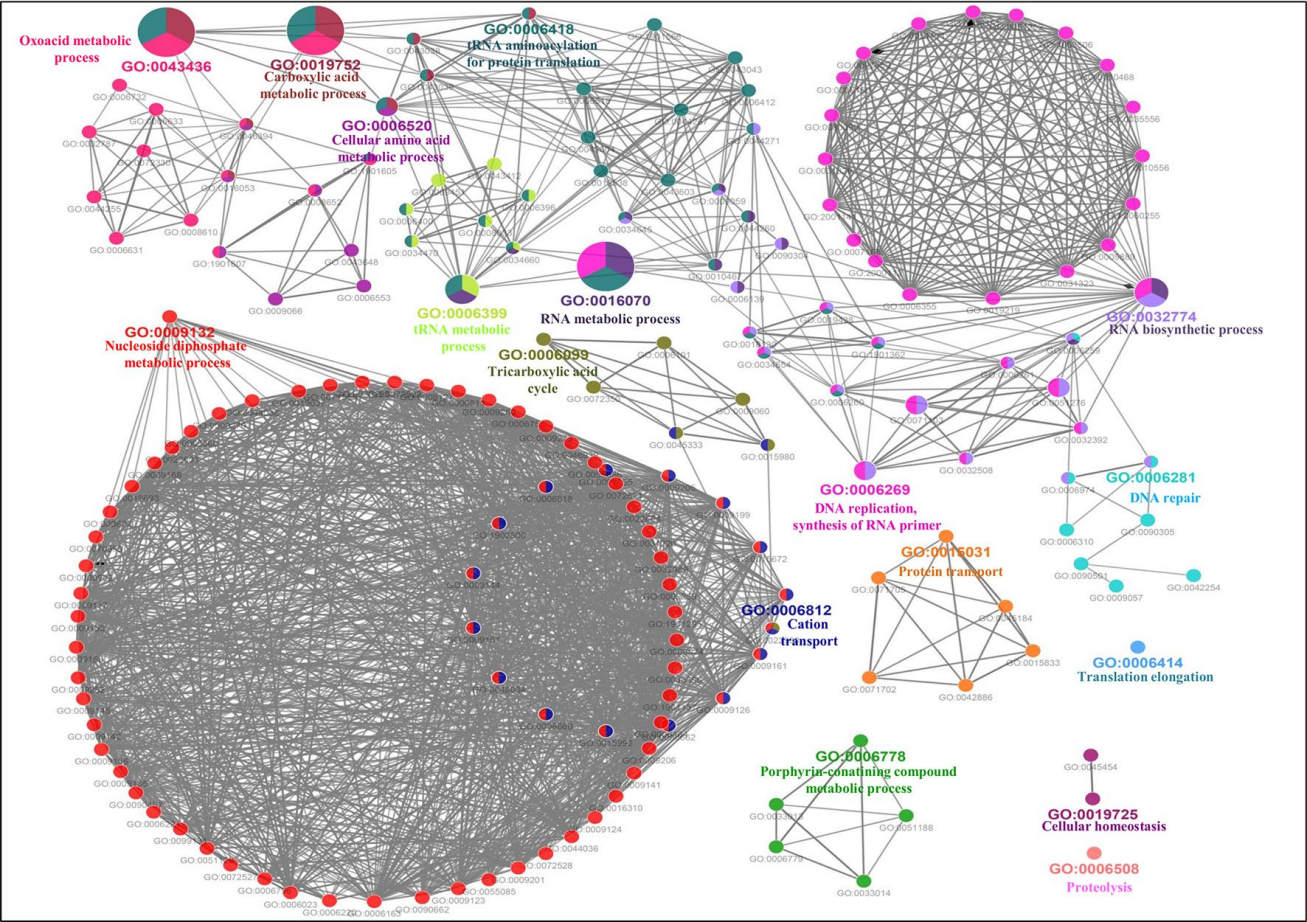
Gene Ontology biological process network of putative MBPs. Functionally enriched GO biological process network of predicted MBPs in *Ott* was computed by ClueGO (at kappa score ≥0.4). Each circle symbolizes a node, i.e., specific GO biological term. The node color designates a specific GO group. The mixed color of the node indicates that specific node is involved in multiple biological processes. A total of 17 GO biological groups were found in the network, and among them, seven were significant with GO biological terms [oxoacid metabolic process (GO:0043436), carboxylic acid metabolic process (GO:0019752), cellular amino acid metabolic process (GO:0006520), tRNA metabolic process (GO:0006399), RNA metabolic process (GO:0016070), RNA biosynthetic process (GO:0032774), and DNA replication, synthesis of RNA primer (GO:0006269)]. This network also supported our domain-based classification that most of the putative MBPs were functional in gene expression and regulation and metabolism.

The GO-enriched biological network of the predicted MBPs consist of 179 nodes and 979 edges which were structured on 17 final kappa score groups. **Figure 6** depicts the 17 groups of GO biological terms, out of which seven (oxoacid metabolic process, carboxylic acid metabolic process, cellular amino acid metabolic process, tRNA metabolic process, RNA metabolic process, RNA biosynthetic process and DNA replication, synthesis of RNA primer) were found significant. The biological node, cellular macromolecule metabolic process (GO:0044260) of groups 10 and 14, and nucleobase-containing compound metabolic process (GO:0006139) of groups 10 and 13 were the most connected GO terms with 112 and 101 links, respectively (**Table S3B**). There were some nodes which were associated with more than two groups (**Table S3B**), implying that multiple cellular processes were regulated by their associated genes.

**Figure 7:**
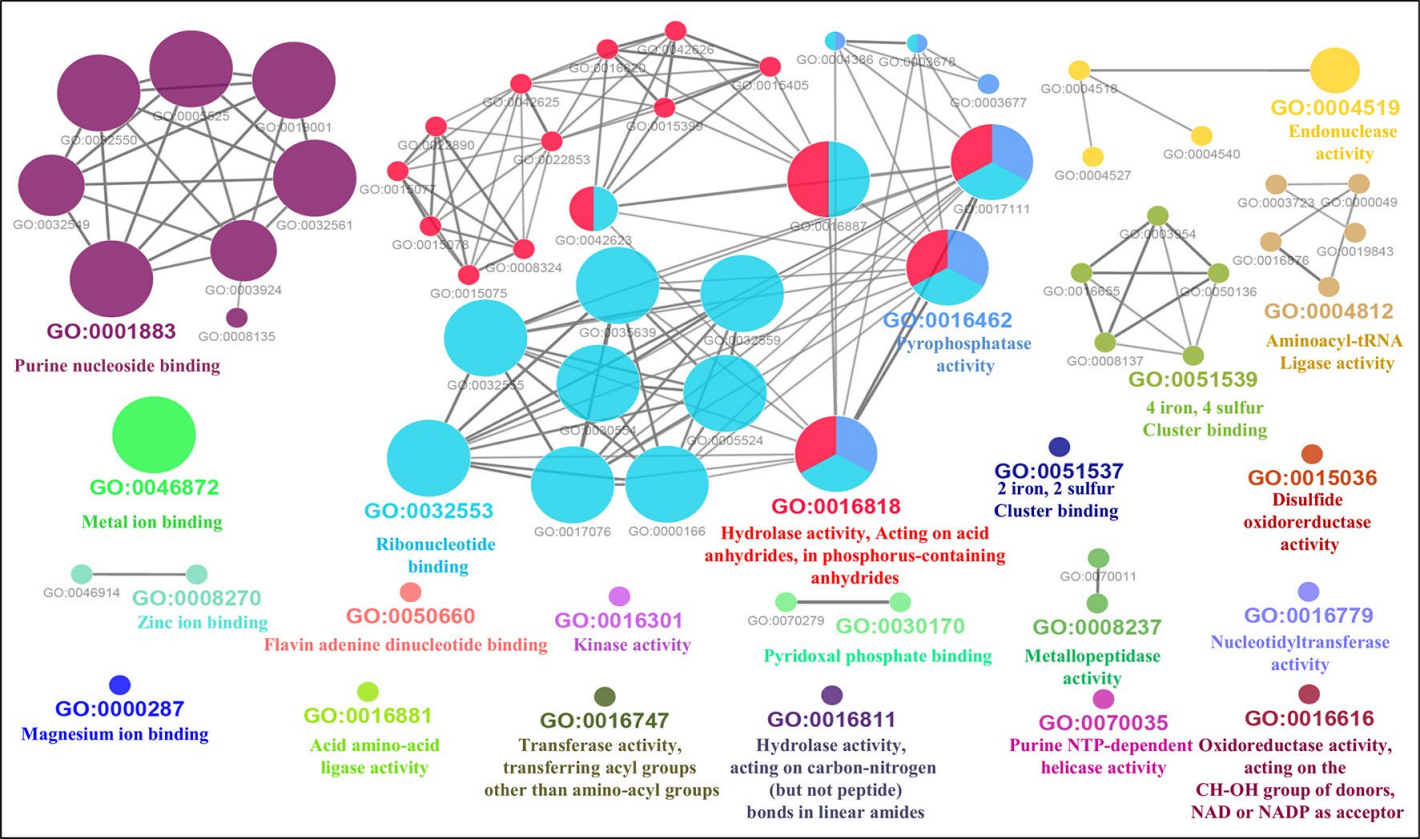
Gene Ontology ClueGO molecular function network of putative MBPs. Functionally enriched GO molecular function network of predicted MBPs was constructed by ClueGO (at kappa score ≥0.4). Each circle in the network represents a node, i.e., specific GO molecular function term and color of the node symbolizes a GO group. The mixed color of the node specifies their involvement in multiple molecular functions. There are 22 final kappa score groups in the GO molecular function network of Ott. Out of 22, 6 were significant groups of GO molecular terms [ribonucleotide binding (GO:0032553), endonuclease activity (GO:0004519), metal ion binding (GO:0046872), purine nucleoside binding (GO:0001883), pyrophosphatase activity (GO:0016462), and hydrolase activity, acting on acid anhydrides, in phosphorus-containing anhydrides (GO:0016818)]. The GO molecular function network also provide pillar for our domain-based and GO biological process classification. This is because molecular activities of most of the significant groups were involved in the processes of gene expression and regulation and metabolism.

Furthermore, the GO-enriched molecular network of predicted MBPs had 67 nodes and 101 edges with 22 kappa score groups. Among these 22, 6 groups with GO molecular terms (ribonucleotide binding, endonuclease activity, metal ion binding, purine nucleoside binding, pyrophosphatase activity and hydrolase activity, acting on acid anhydrides, in phosphorus-containing anhydrides) were found significant (**Figure 7**). The nucleotide binding (GO:0000166) node was the most linked GO term with 111 links (**Table S3C**). The results of GO annotation favored the domain and literature-based functional classification as large number of predicted MBPs was involved in metabolism and gene expression and regulation.

The higher number of MBPs with GO terms related to metabolism was expected. This is because by means of metabolism, microbial pathogen attains energy and nutrients which ultimately helps in their growth and survival. It is reported earlier that the MBPs involved in metabolism play important role(s) in various biosynthetic pathways, electron transport, and in signal transduction (Andreini et al., 2007a; Liu et al., 2014; Bridwell-Rabb et al., 2016). The GO network of putative MBPs was also enriched with the GO terms involved in gene expression and regulation. Previously, it was known that MBPs perform functions in DNA replication, repair, and recombination and RNA synthesis and processing (Anantharaman et al., 2002; Skaar, 2010; Porcheron et al., 2013). The functional classification and GO analysis of MBPs of *Ott* suggested that MBPs have diverse role(s) in its proliferation and endurance.

### Toxin Prediction and Potential Therapeutic Target Prioritization

The protein toxins (virulent proteins) are produced by pathogenic bacteria in order to endure in the host environment and to improve their survival. The development of high throughput techniques helps in better understanding the role of bacterial toxins (assist in pathogenicity) at various molecular and cellular levels. We have identified that a total of 1,114 proteins (∼84%) were putative virulent from the whole proteome of *Ott* strain Ikeda. Out of 321 putative MBPs in *Ott*, 245 proteins (comprised of ∼22% of the total virulent proteome of *Ott*) were predicted as prospective bacterial toxins that may confer to the pathogenicity. Furthermore, targeting homologous proteins may lead to cytotoxicity and cross-reactivity of drug compounds within the host (Singh et al., 2017; Sharma et al., 2018b). Therefore, pathogen-specific MBPs were identified by BLASTp search of selected 245 putative virulent MBPs against *Homo sapiens* proteins (human; taxid: 9906). We obtained 98 nonhomologous proteins which were specific to *Ott*. These 98 nonhomologous putative virulent MBPs were prioritized as potential therapeutic candidates (**Table S3D**). Rest 147 MBPs showed homology with the human proteins; therefore, these proteins were excluded from further analysis. It is a well-known fact that MBPs have vital role(s) in the pathogenesis of various bacterial pathogens of human (Shafeeq et al., 2013; Hameed et al., 2015). It is also documented that MBPs were a promising player in the process of drug discovery (Jamieson et al., 2003; Hameed et al., 2015; Mojica et al., 2016). Therefore, nonhomologous putative virulent MBPs of *Ott* may have the potential to act as favorable therapeutic targets.

Furthermore, to figure out most suitable drug targets being nonhomologous and being vital for virulence and survival are not only the parameters but also includes other important criteria like physiochemical properties (molecular weight, aliphatic index, theoretical pI, and GRAVY) and ability to interact with potential drugs, i.e., druggability analysis (Sharma and Kumar, 2016). The molecular weight of the proteins should be low (≤110 kDa) for acting as more significant target due to easy purification in wet lab examination (Parvege et al., 2014). We have found that molecular weight of 97 proteins range from 7.649 kDa (WP_012460740.1) to 105.999 kDa (WP_012461905.1), and a single protein (WP_012460745.1) has molecular weight 133.456 kDa. The thermostability of the proteins is positively connected with the aliphatic index, i.e., higher indices are observed in thermostable proteins than the other proteins (Ikai, 1980). We have observed that the aliphatic index of predicted virulent MBPs ranged from 55.88 (WP_012460740.1) to 128.85 (WP_012460966.1), and theoretical pI of 41 proteins was <7 and that of 57 proteins were >7. The GRAVY ranges from −1.165 for WP_012462272.1 to 0.39 for WP_012462319.1. For better interaction of proteins with water molecules, low value of GRAVY is vital. The physiochemical properties of all putative virulent nonhomologous MBPs of *Ott* are summarized in **Table S3E**.

The assumption behind the druggability analysis is that the druggable targets have the ability to interact with the drug or drug-like molecules (Sharma and Kumar, 2016). Therefore, to identify homologous drug targets, the shortlisted putative virulent nonhomologous 98 MBPs of *Ott* were subjected to BLASTp search at E-value of 0.00001 against the DrugBank database. Sixty proteins have satisfied the mentioned criteria and considered as significant homologues (potential druggable targets). The rest of the proteins (nonhit) were considered as novel possible drug targets which need experimental validation.

Furthermore, the functional annotation which we have done in the earlier step also supports the finding that the selected 98 putative virulent MBPs may act as probable drug targets. The shortlisted putative virulent 98 MBPs categorized into 8 broad functional classes (gene expression and regulation, metabolism, cell signaling, transport, stress response regulator, protein folding, antimicrobial resistance, and posttranslational modification). In the class of gene expression and regulation, we have found general enzymes involved in: (i) DNA replication, repair, and recombination (DnaB-like helicase, DNA polymerases, DNA gyrase, DNA topoisomerase, DnaG, Thymidylate kinase, AP endonuclease 1, UvrABC, UvrD, uracil-DNA glycosylase, RecA, RuvC, integrase, CinA), (ii) transcription, translation, and RNA processing (RNA polymerase sigma factor, ribonucleae E/G, YbeY, NsuB, RppH, GreA/GreB, Rho, Prisomal protein N, DksA, zinc-binding ribosomal protein, IF-1, phyenylalanine-tRNA ligase), and (iii) chromosome partition and condensation (ParA, ParB, IHF-like DNA-binding proteins, and tubulin/FtsZ). From the literature review, we found that these general regulatory proteins play important roles in growth, survival, adaptation, stress responses, and virulence in most of the bacterial pathogens (Nakayama et al., 2008; Cho et al., 2010). Earlier, it is stated that architecture and conservation of bacterial DNA replication and transcription are effective target for designing broad-spectrum antibacterial agents (Robinson et al., 2012; Ma et al., 2016). Karkare et al. (2012) proposed a model for the modulation of DNA gyrase activity of *M. tuberculosis* by Ca^2+^ binding (Karkare et al., 2012). The overexpression of GreA proteins of *E. coli* showed resistance to the toxic levels of Zn^2+^ and Mn^2+^ divalent metal ions, which indicate that GreA helps in their survival during harsh conditions (Stepanova et al., 2007). YbeY is a highly conserved, metal-dependent (bind to Ni^2+^) protein known to play a role in the processing of 3’ end of 16S rRNA, stress, and virulence regulation in bacteria (Vercruysse et al., 2014). DksA contains a zinc-finger motif and play a role in posttranscriptional regulation of quorum sensing dependent virulence genes in *P. aeruginosa* (Jude et al., 2003). TilS enzyme is involved in tRNA processing, as it catalyzes the formation of lysidine using lysine and ATP as substrate and Mg^2+^ as a cofactor (Suzuki and Miyauchi, 2010). It is also known that TilS is a highly conserved and essential enzyme in bacteria and therefore act as a suitable target for broad-spectrum antimicrobial agent (Suzuki and Miyauchi, 2010). Furthermore, an inhibitor against ParA protein of *M. tuberculosis* has been designed previously, and it is stated that Mg^2+^ ion significantly stimulates ATPase activity of ParA (Nisa et al., 2010). It is known that tubulin-like GTPase, FtsZ, is regulated by Ca^2+^ concentration, essential for cytokinesis in bacterial cell and recognized as an excellent drug target (Erickson et al., 2010). Recently, a report indicates that a novel alpha-pyrone compound “corallopyroninA” inhibit RNA polymerase switch region of *Orientia* and therefore can act as a potent therapeutic target for scrub typhus (Kock et al., 2018).

The class of metabolism list up the proteins involved in (i) biosynthesis of bacterial cell wall [MurA, MurB, MurC, MurD, D-alanine-D-alanine ligase (DDI), DapD_Trfase_Hexpep_rpt, and Aspartate kinase], (ii) amino acid metabolism [acetyglutamate kinase, glutamine synthetase (GS)], (iii) carbohydrate metabolism [glycoside hydrolase family 18 (GH), glycoside transferase family 2 (GT), N-acetylmuramoyl-L-alanine amidase], (iv) nucleotide metabolism (SAICAR synthetase, deoxycytidine triphosphate deaminase), (v) fatty acid metabolism [phosphopantetheinyl transferase (PPTase)], (vi) energy generation (citrate synthase, 7Fe-ferredoxin, pyruvate, phosphate kinase), and (vii) oxidation of alcohol (PQQ-ADH). Previously, an *in silico* study stated that metabolic pathway proteins of *Ott* may act as probable target for drug discovery process (Sharma et al., 2018b). It is a well-established fact that Mur enzymes are involved in biosynthesis of bacterial cell wall using divalent metal ions as cofactor and are best intracellular therapeutic targets (Munshi et al., 2013; Moraes et al., 2015; Jukič et al., 2019). DDI enzyme uses Mg^2+^ ion as cofactor and catalyzes early steps of peptidoglycan synthesis, i.e., formation of D-Ala-D-Ala and D-Ala-D-Ser dipeptides in bacteria (Tytgat et al., 2009). The enzyme DapD uses Mg^2+^ ion for its stability and provides L-lysine and its immediate precursor meso-diaminopimelate, which are critical for cell-wall synthesis in most of the bacteria (Schuldt et al., 2009). It is reported previously that acetylglutamate kinase is a key enzyme in the synthesis of arginine, and aspartate kinase is involved in the synthesis of various amino acids (L-aspartate, threonine, methionine, isoleucine, and lysine and its precursor diaminopimelate); in addition, both the enzymes have affinity for Mg^2+^ (Ramón-Maiques et al., 2002; Chaitanya et al., 2010). All these enzymes (Mur, DDI, DapD, aspartate kinase, and acetyglutamate kinase) are absent in human and, therefore, may serve as appropriate targets for inhibition (Ramón-Maiques et al., 2002; Schuldt et al., 2009; Tytgat et al., 2009; Chaitanya et al., 2010; Munshi et al., 2013; Moraes et al., 2015). Glutamine synthase catalyzes the condensation of L-glutamate and ammonia to form glutamine, requires Mg^2+^ or Mn^2+^ for reaction, plays a central role in nitrogen metabolism, and therefore can be a suitable chemotherapeutic target. Earlier, inhibition of GS is studied in *Bacillus subtilis* and *M. tuberculosis* (Murray et al., 2013). SAICAR synthetase catalyzes the seventh step of *de novo* purine-biosynthesis pathway using ATP and Mg^2+^ (Wolf et al., 2014). Wolf et al. (2014) also reported the structural differences in the active site of SAICAR synthetase (PurC) of *Streptococcus pneumonia* and human, which provide the basic framework for designing structure based inhibitors against bacterial PurC (Wolf et al., 2014). GH and GT enzymes are important for breakdown and synthesis of glycoside bonds, respectively. It is documented previously that these carbohydrate active enzymes are gaining importance as drug targets, and some members of these enzymes require divalent metal ions for their activity (Payne et al., 2012; Ardèvol and Rovira, 2015; Schmid et al., 2016). The cell-wall hydrolase N-acetylmuramoyl-L-alanine amidase has Zn^2+^-dependent activity and is earlier known to play a role in peptidoglycan catabolic process and bacterial cell division (Vermassen et al., 2019). Cellular dUTPase have affinity for Mg^2+^ and catalyzes hydrolysis of dUTP to dUMP, therefore is necessary for DNA metabolism and bacterial survival (Hizi and Herzig, 2015). Previously, 7Fe-ferredoxin (FdxA), which contains both 3Fe–4S and 4Fe–4S cluster, is used as a drug target in *M. tuberculosis* (Ugalde et al., 2019). A study on *P. aeruginosa* stated priorly that PPTases are involved in the synthesis of fatty acid and siderophores, require Mg^2+^ ion for the reaction, and are viable target for development of antibiotics (Finking et al., 2002).

The cell signaling class was found to be enriched in HD domain. The other domains found in this class were OmpR/PhoB-type DNA binding, GGDFF, EAL, and phopholipase D/transphosphatidylase. The role of HD domain has already discussed in the above section. OmpR/PhoB DNA-binding proteins act as two-component signal transduction transcriptional regulator and have the ability to bind Mg^2+^ ion, which act as cofactor for its catalytic activity (Sola et al., 2006). Previously, it is documented that the two-component systems serve as satisfactory target for drug designing because of their inference in virulence, resistance to the drug, their universality in bacteria, and their absence in humans (Blanco et al., 2002). It is a well-known fact that cyclic-GMP signaling in bacteria regulates wide range of functions like adhesion, biofilm formation, and virulence (Ryan et al., 2006). Both GGDFF and EAL protein domains are involved in cyclic Di-GMP signaling; GGDFF domain synthesizes cyclic di-GMP; on the other side, EAL domain catalyzes cyclic-GMP hydrolysis; and both domains require divalent metal ion as cofactor (Mg^2+^ or Mn^2+^) (Ryan et al., 2006). Earlier, it is reported that the bacterial phospholipase D is involved in the hydrolysis of phospholipids and generates metabolites that act as secondary messengers, which further play roles in host cell invasion, modulation of lipid content of host cell membrane, and pathogenesis (Flores-Díaz et al., 2016). The Zn^2+^-binding ability of phospholipase D has also been reported earlier in *Bacillus subtilis* G-22 (Garutskas et al., 1977).

The periplasmic bacterial solute-binding protein (SBP), type-IV secretion protein TraC, MgtE intracellular domain, Tol-Pal system/TolQ, and cation efflux protein were mentioned under transport category. A study on periplasmic SBP of *Paracoccus denitrificans* indicates that SBP binds to Zn ion, helps in its transport through ABC transporter, and therefore, can act as potent antibacterial target (Neupane et al., 2017). Earlier, it is known that *Ott* have massively proliferating conjugative transfer system (Cho et al., 2007), and TraC protein was probably involved in conjugal transfer, synthesis, and assembly of F conjugative pilus (Schandel et al., 1992). It is reported previously in *P. aeruginosa* that inner membrane MgtE protein helps in the transport of magnesium ion, stimulates rsmYZ transcription to inhibit gene expression of type-III secretion system, and therefore, act as virulence modulator (Chakravarty et al., 2017). Earlier, it is stated that the Tol-Pal system acts as ion potential-driven molecular motor in a wide range of Gram-negative bacteria, which help them to maintain the stability of their outer membrane and export of cell envelope component (Cascales et al., 2001). The cation efflux protein was reported earlier to raise the tolerance against Zn, Cd, Co, and Ni and help to remove the excess of these metal ions in order to maintain metal homeostasis (Stähler et al., 2006).

We have found Ppx/GppA phosphatase, thioredoxin reductase, and divalent ion tolerance protein (CutA) in the category of stress response regulator. Earlier, a study on Ppx/GppA phosphatase of *Aquifex aeolicus* indicated that these enzymes require metal ions (Ca^2+^) for their catalysis and play crucial roles in bacterial stringent response triggered by starvation (Kristensen et al., 2004). The roles of thioredoxin reductase in numerous cellular processes and regulation of oxidative stress are well known. Furthermore, earlier studies also reported that thioredoxin reductases are viable target for inhibition using antibiotics and metal binding (Rollin‐Genetet et al., 2004; Lu et al., 2013). The small CutA protein is universally distributed in a broad range of bacteria, and its role in divalent metal tolerance (uptake, storage, delivery, and efflux) has been documented earlier in *E. coli*. (Rensing and Grass, 2003). We found two metallo-β-lactamase in our study that putatively have binding site for Zn, Mn, and Ca and probably involved in antimicrobial resistance. It is documented formerly that metallo-β-lactamase has affinity for cations (Zn, Fe, Mn) which acts as cofactor for the enzyme (Palzkill, 2013). Earlier, a Ca-EDTA inhibitor was found efficient for metallo-β-lactamase in a mouse model of *P. aeruginosa* pneumonia (Aoki et al., 2010).

Protein folding category contains a copper chaperones PCu(A) having affinity for Cu^2+^ and a chaperonin 10 protein having affinity for Ca^2+^. The role of copper chaperones PCu(A) is earlier noticed in the biogenesis and assembly of respiratory enzyme complexes (Thompson et al., 2012). Roberts et al. (2003) reported that chaperonin 10 plays a role in folding of structural protein domains, transport of proteins, ATP, and peptide (Roberts et al., 2003). Furthermore, it is also stated that chaperonin 10 acts as a virulent factor and helps to maintain stress response in *M. tuberculosis* (Roberts et al., 2003). In the class of posttranslational modification, we have found tRNA threonylcarbamoyl adenosine modification protein (TsaE). It is reported previously that TsaE interferes in pre- or postcatalytic step of t^6^A tRNA modification and has affinity for Mg^2+^, and its mechanism of action and binding mode make it a suitable antimicrobial target (Missoury et al., 2018). These findings and facts supported our study that the predicted virulent nonhomologous MBPs may act as appropriate targets for developing drug against the pathogen.

## Conclusion

In the presented work, we have integrated multiple bioinformatics tools aimed to identify putative MBPs of zoonotic pathogen *Ott*. The analysis showed that about a quarter of pathogen proteome was enriched with metals and was pervasive with magnesium. The prediction of MBPs in different subcellular compartments of the bacterial cell has documented that most of the MBPs localized in the cytoplasm. The involvement of MBPs in diverse cellular and biological processes has been noted through their functional domain classification. Furthermore, the druggability analysis of putative MBPs indicate that these may act as druggable targets. In conclusion, this study provides the repository of putative MBPs which might serve as primer for experimental validation. These putative MBPs may have the ability to act as therapeutic targets for developing metal-based antimicrobial agent against the pathogenic *Ott*.

## Data Availability

Publicly available datasets were analyzed in this study. This data can be found here: https://www.ncbi.nlm.nih.gov/refseq/.

## Author Contributions

DS and SKV conceived the idea and designed the experiments. DS predicted the MBPs of *Ott*. AS performed functional classification and clustering. DS predicted the proteins 3D structure. AS and DS performed mining of MBPs of *Ott* and analyzed the data. DS wrote the manuscript. SKV and BS checked the manuscript. All authors read and approved the manuscript.

## Conflict of Interest Statement

The authors declare that the research was conducted in the absence of any commercial or financial relationships that could be construed as a potential conflict of interest.
