# Supplementary material for: Bioinformatic Exploration of Metal-Binding Proteome of Zoonotic Pathogen Orientia tsutsugamushi
Source: Front Genet. 2019 Sep 24;10:797. doi: 10.3389/fgene.2019.00797 (PMC6769048; doi:10.3389/fgene.2019.00797)
Supplement: Supplementary file 1 [file Table_1.docx]

Supplementary Material

Bioinformatic exploration of metal-binding proteome of zoonotic pathogen *Orientia tsutsugamushi*

Dixit Sharma^1^, Ankita Sharma^2*^, Birbal Singh^2^, Shailender Kumar Verma^1*#^

^1^Centre for Computational Biology and Bioinformatics, School of Life Sciences, Central University of Himachal Pradesh, Shahpur, Himachal Pradesh. INDIA-176206

^2^ICAR-Indian Veterinary Research Institute, Regional Station, Palampur, Himachal Pradesh. INDIA-176061

# Supplementary Tables

**Supplementary Table S1.List of putative metal-binding proteins (MBPs) from proteome of *O. tsutsugamushi*.**

| **S.No.** | **Metals** | **Author's created database search results (metal binding sequences downloaded from Uniprot) (No. of Proteins)** | **Metal PDB results (No. of Proteins)** |
| --- | --- | --- | --- |
| 1 | Magnesium | 371 | 210 |
| 2 | Calcium | 469 | 118 |
| 3 | Zinc | 678 | 83 |
| 4 | Iron | 622 | 52 |
| 5 | Manganese | 506 | 72 |
| 6 | Nickel | 396 | 17 |
| 7 | Cadmium | 381 | 32 |
| 8 | Cobalt | 92 | 13 |
| 9 | Copper | 556 | 8 |
| Putative metal binding proteins after two steps | | | 605 |
| Putative metal binding proteins after removing repeated sequences | | | 345 |

**Supplementary Table S2A.Details of interacting amino acid residues of with Mg^2+^.**

| **S.No.** | **Sequence ID** | **Binding Residues** | **Template** | **Score** |
| --- | --- | --- | --- | --- |
| 1 | WP_012461476.1 | 103E ,  107I | 1iruG2 | 1.704 |
| 2 | WP_012461492.1 | 288D ,  290D | 1ecbC0 | 1.814 |
| 3 | WP_012461546.1 | 70T ,  71S | 1ezwA0 | 1.77 |
| 4 | WP_012461508.1 | 58R ,  59F | 1iru11 | 1.349 |
| 5 | WP_012461532.1 | 40D ,  41E | 1dqnA0 | 1.529 |
| 6 | WP_012461533.1 | 294T ,  295S | 1ezwA0 | 1.927 |
| 7 | WP_012461493.1 | 263K ,  264S | 1jwyB0 | 1.867 |
| 8 | WP_012461549.1 | 133D ,  134E | 1bwvA0 | 1.771 |
| 9 | WP_012461551.1 | 353E ,  355E | 1a77_0 | 1.63 |
| 10 | WP_012461552.1 | 244K ,  246S | 1jqvA0 | 1.747 |
| 11 | WP_012461555.1 | 795E ,  796D | 1grv_0 | 1.878 |
| 12 | WP_012461596.1 | 105E ,  109I | 1iruG2 | 1.703 |
| 13 | WP_012461600.1 | 22K ,  23S | 1jwyB0 | 1.666 |
| 14 | WP_012461602.1 | 208N ,  211D | 1auk_0 | 1.444 |
| 15 | WP_012461603.1 | 238Q ,  239T ,  240K , 258D | 1f1zA1 | 2.006 |
| 16 | WP_012461605.1 | 20V ,  21D | 1k77A1 | 1.86 |
| 17 | WP_012461609.1 | 198I ,  199D | 1k77A1 | 1.279 |
| 18 | WP_012461626.1 | 209K ,  211S | 1jqvA0 | 1.731 |
| 19 | WP_012461632.1 | 295K ,  296S | 1jwyB0 | 1.845 |
| 20 | WP_012461635.1 | 89M ,  92N | 1ne9A2 | 1.409 |
| 21 | WP_012461641.1 | 225T ,  226S | 1ezwA0 | 1.469 |
| 22 | WP_012461643.1 | 460E ,  461D | 1grv_1 | 1.866 |
| 23 | WP_012461655.1 | 223D ,  227D | 1h1lB0 | 1.845 |
| 24 | WP_012461666.1 | 271V ,  275G | 1nr9A0 | 1.908 |
| 25 | WP_012461670.1 | 196K ,  197S | 1jwyB0 | 1.718 |
| 26 | WP_012461681.1 | 63E ,  67I | 1iruG2 | 1.477 |
| 27 | WP_012461713.1 | 588V ,  589D | 1k77A1 | 1.771 |
| 28 | WP_012461722.1 | 378H ,  379D | 1occA0 | 1.68 |
| 29 | WP_012461726.1 | 188K ,  189S | 1jwyB0 | 1.74 |
| 30 | WP_012461727.1 | 79E ,  80D | 1grv_1 | 1.715 |
| 31 | WP_012461746.1 | 38E ,  39D | 1grv_1 | 1.684 |
| 32 | WP_012461769.1 | 19D ,  57E ,  79D | 1hmv_2 | 1.949 |
| 33 | WP_012461775.1 | 223K ,  224S | 1jwyB0 | 1.704 |
| 34 | WP_012461805.1 | 243E ,  247I | 1iruG2 | 1.804 |
| 35 | WP_012461809.1 | 89L ,  92N | 1ne9A2 | 1.424 |
| 36 | WP_012461831.1 | 591D ,  592E | 1bwvA0 | 1.811 |
| 37 | WP_012461854.1 | 223K ,  224S | 1jwyB0 | 1.854 |
| 38 | WP_012461858.1 | 37K ,  38S | 1jwyB0 | 1.903 |
| 39 | WP_012461866.1 | 224K ,  225S | 1jwyB0 | 1.909 |
| 40 | WP_012461868.1 | 259Q ,  260L | 1frfL0 | 1.902 |
| 41 | WP_012461879.1 | 325T ,  326S | 1ezwA0 | 1.813 |
| 42 | WP_012461880.1 | 409H ,  413H | 1ka2A0 | 1.731 |
| 43 | WP_012461888.1 | 107G ,  108P | 1iru10 | 1.17 |
| 45 | WP_012461889.1 | 176T ,  177S | 1ezwA0 | 1.941 |
| 45 | WP_012461890.1 | 45K ,  46S | 1jwyB0 | 1.87 |
| 46 | WP_012461905.1 | 38K ,  40S | 1jqvA0 | 1.878 |
| 47 | WP_012461920.1 | 234K ,  236S | 1jqvA0 | 1.522 |
| 48 | WP_012461925.1 | 112K ,  113S | 1jwyB0 | 1.797 |
| 49 | WP_012461946.1 | 196K ,  197S | 1jwyB0 | 1.716 |
| 50 | WP_012461949.1 | 259K ,  260S | 1jwyB0 | 1.501 |
| 51 | WP_012461953.1 | 371K ,  372S | 1jwyB0 | 1.79 |
| 52 | WP_012461957.1 | 105E ,  109I | 1iruG2 | 1.702 |
| 53 | WP_012461967.1 | 147I ,  148D | 1k77A1 | 1.552 |
| 54 | WP_012461990.1 | 24L ,  27N | 1ne9A2 | 1.523 |
| 55 | WP_012462008.1 | 218D ,  219N | 1h1dA0 | 1.844 |
| 56 | WP_012462035.1 | 338D ,  339D ,  344S | 1gxbD0 | 1.768 |
| 57 | WP_012462041.1 | 125T ,  127N | 1q9sA0 | 1.69 |
| 58 | WP_012462043.1 | 523T ,  524S | 1ezwA0 | 1.873 |
| 59 | WP_012462044.1 | 188K ,  189S | 1jwyB0 | 1.74 |
| 60 | WP_012462053.1 | 23V ,  24D | 1k77A1 | 1.394 |
| 61 | WP_012462078.1 | 65I ,  68N | 1ne9A2 | 1.467 |
| 62 | WP_012462079.1 | 15K ,  16S | 1jwyB0 | 1.874 |
| 63 | WP_012462083.1 | 185T ,  188R ,  190D | 1rk2B0 | 1.237 |
| 64 | WP_012462089.1 | 132E ,  134H ,  141E | 1bglA0 | 1.596 |
| 65 | WP_012462109.1 | 193I ,  194D | 1k77A1 | 1.604 |
| 66 | WP_012462134.1 | 103E ,  107I | 1iruG2 | 1.703 |
| 67 | WP_012462153.1 | 103E ,  107I | 1iruG2 | 1.689 |
| 68 | WP_012462160.1 | 91S ,  92Y ,  93V | 1iruG0 | 1.31 |
| 69 | WP_012462162.1 | 135D ,  136E | 1bwvA0 | 1.676 |
| 70 | WP_012462208.1 | 17V ,  18G | 1nr9A0 | 1.653 |
| 71 | WP_012462223.1 | 354K ,  355S | 1jwyB0 | 1.791 |
| 72 | WP_012462224.1 | 43K ,  44S | 1jwyB0 | 1.86 |
| 73 | WP_012462244.1 | 31D ,  35D | 1h1lB0 | 1.532 |
| 74 | WP_012462254.1 | 131E ,  135I | 1iruG2 | 1.777 |
| 75 | WP_012462259.1 | 197K ,  198S | 1jwyB0 | 1.717 |
| 76 | WP_012462261.1 | 103E ,  107I | 1iruG2 | 1.705 |
| 77 | WP_012462271.1 | 98D ,  99E | 1bwvA0 | 1.5 |
| 78 | WP_012462273.1 | 170K ,  172S | 1jqvA0 | 1.523 |
| 79 | WP_012462274.1 | 295K ,  297S | 1jqvA0 | 1.781 |
| 80 | WP_012462277.1 | 228H ,  229H | 1nmpA0 | 1.46 |
| 81 | WP_012462279.1 | 101T ,  155S | 1f5nA0 | 1.468 |
| 82 | WP_012462280.1 | 429V ,  430D | 1k77A1 | 1.777 |
| 83 | WP_012462281.1 | 446D ,  447N | 1auk_0 | 1.747 |
| 84 | WP_012462282.1 | 523D ,  524E | 1dqnA0 | 1.534 |
| 85 | WP_012462286.1 | 245K ,  246S | 1jwyB0 | 1.816 |
| 86 | WP_012462316.1 | 167K ,  169S | 1jqvA0 | 1.848 |
| 87 | WP_012462317.1 | 40D ,  41N | 1h1dA0 | 1.904 |
| 88 | WP_012462322.1 | 3I ,  4D | 1k77A1 | 1.671 |
| 89 | WP_012462325.1 | 99V ,  100G | 1nr9A0 | 1.37 |
| 90 | WP_012462327.1 | 87K ,  88S | 1jwyB0 | 1.803 |
| 91 | WP_012462340.1 | 374K ,  375S | 1jwyB0 | 1.79 |
| 92 | WP_011944233.1 | 193E ,  194D | 1grv_1 | 1.496 |
| 93 | WP_011944565.1 | 17V ,  58V ,  104K , 105W ,  106F | 1iruG0 | 1.452 |
| 94 | WP_011945084.1 | 41L ,  42L | 1ewkB0 | 1.482 |
| 95 | WP_012460728.1 | 363K ,  365S | 1jqvA0 | 1.692 |
| 96 | WP_012460731.1 | 178D ,  179E | 1bwvA0 | 1.715 |
| 97 | WP_012460738.1 | 185D ,  186E | 1bwvA0 | 1.767 |
| 98 | WP_012460745.1 | 634D ,  635D | 1ecbC0 | 1.829 |
| 99 | WP_012460747.1 | 378L ,  381N | 1ne9A2 | 1.821 |
| 100 | WP_012460754.1 | 130L ,  133N | 1ne9A2 | 1.888 |
| 101 | WP_012460755.1 | 10D ,  12E | 1bwvA0 | 1.529 |
| 102 | WP_012460772.1 | 192L ,  195N | 1ne9A2 | 1.738 |
| 103 | WP_012460797.1 | 183V ,  184I | 1iruG0 | 1.67 |
| 104 | WP_012460803.1 | 198V ,  200D | 1k77A1 | 1.534 |
| 105 | WP_012460805.1 | 174A ,  176L | 1nrjB1 | 1.471 |
| 106 | WP_012460810.1 | 137M ,  138I ,  140F , 142W ,  143I | 1iq8A0 | 1.893 |
| 107 | WP_012460871.1 | 174I ,  175D | 1k77A1 | 1.63 |
| 108 | WP_012460873.1 | 34D ,  36D | 1mxgA0 | 1.209 |
| 109 | WP_012460882.1 | 115K ,  117S | 1jwyB0 | 1.856 |
| 110 | WP_012460884.1 | 103E ,  107I | 1iruG2 | 1.704 |
| 111 | WP_012460897.1 | 137K ,  138S | 1jwyB0 | 1.892 |
| 112 | WP_012460901.1 | 137K ,  138S | 1jwyB0 | 1.892 |
| 113 | WP_012460919.1 | 171L ,  172L | 1ewkB0 | 1.848 |
| 114 | WP_012460923.1 | 77A ,  78V ,  83R ,  84I , 86F | 1iq8A0 | 1.487 |
| 115 | WP_012460938.1 | 416V ,  417D | 1k77A1 | 1.882 |
| 116 | WP_012460949.1 | 263H ,  267H ,  293E | 1ka2A0 | 2.513 |
| 117 | WP_012460950.1 | 116D ,  117D | 1j9jA0 | 1.781 |
| 118 | WP_012460952.1 | 612K ,  613S | 1jwyB0 | 1.82 |
| 119 | WP_012460958.1 | 72V ,  73G | 1nr9A0 | 1.752 |
| 120 | WP_012460959.1 | 103E ,  107I | 1iruG2 | 1.702 |
| 121 | WP_012460962.1 | 105E ,  109I | 1iruG2 | 1.704 |
| 122 | WP_012460963.1 | 259N ,  261Q | 1l8a_0 | 1.891 |
| 123 | WP_012460965.1 | 132K ,  134S | 1jqvA0 | 1.799 |
| 124 | WP_012460966.1 | 116D ,  117D | 1ecbC0 | 1.744 |
| 125 | WP_012460972.1 | 5D ,  6E | 1dqnA0 | 1.537 |
| 126 | WP_012460979.1 | 185T ,  186S | 1ezwA0 | 1.706 |
| 127 | WP_012460984.1 | 32V ,  33D | 1k77A1 | 1.786 |
| 128 | WP_012460990.1 | 196K ,  197S | 1jwyB0 | 1.716 |
| 129 | WP_012460992.1 | 103E ,  107I | 1iruG2 | 1.705 |
| 130 | WP_012460994.1 | 339K ,  340S | 1jwyB0 | 1.65 |
| 131 | WP_012461000.1 | 74L ,  77N | 1ne9A2 | 1.456 |
| 132 | WP_012461003.1 | 8D ,  9D | 1ecbC0 | 1.775 |
| 133 | WP_012461013.1 | 103E ,  107I | 1iruG2 | 1.703 |
| 134 | WP_012461015.1 | 164K ,  165S | 1jwyB0 | 1.716 |
| 135 | WP_012461055.1 | 104D ,  106D | 1mc3A0 | 1.508 |
| 136 | WP_012461063.1 | 45K ,  46S | 1jwyB0 | 1.869 |
| 137 | WP_012461072.1 | 593K ,  595S | 1jqvA0 | 1.653 |
| 138 | WP_012461076.1 | 185T ,  186S | 1ezwA0 | 1.706 |
| 139 | WP_012461080.1 | 175N ,  176P | 1iru10 | 1.422 |
| 140 | WP_012461099.1 | 116K ,  118S | 1jqvA0 | 1.435 |
| 141 | WP_012461101.1 | 54K ,  55S | 1jwyB0 | 1.819 |
| 142 | WP_012461104.1 | 424D ,  428D | 1h1lB0 | 1.712 |
| 143 | WP_012461125.1 | 103E ,  107I | 1iruG2 | 1.705 |
| 144 | WP_012461155.1 | 350S ,  352S | 1pt6B0 | 1.82 |
| 145 | WP_012461161.1 | 331Q ,  332I ,  333K | 1f1zA1 | 1.876 |
| 146 | WP_012461164.1 | 198D ,  202E | 1yveI0 | 1.502 |
| 147 | WP_012461165.1 | 309T ,  310S | 1ezwA0 | 1.854 |
| 148 | WP_012461168.1 | 429D ,  430R | 1iruX3 | 1.669 |
| 149 | WP_012461170.1 | 57K ,  58S | 1jwyB0 | 1.849 |
| 150 | WP_012461184.1 | 143I ,  144D | 1k77A1 | 1.619 |
| 151 | WP_012461185.1 | 101D ,  102D | 1ecbC0 | 1.833 |
| 152 | WP_012461219.1 | 158D ,  159D | 1j9jA0 | 1.742 |
| 153 | WP_012461229.1 | 402E ,  403D | 1grv_1 | 1.744 |
| 154 | WP_012461232.1 | 123D ,  126E | 1dxeA0 | 1.723 |
| 155 | WP_012461239.1 | 92K ,  93S | 1jwyB0 | 1.533 |
| 156 | WP_012461246.1 | 10K ,  11E | 1h1lB1 | 1.536 |
| 157 | WP_012461247.1 | 428K ,  429S | 1jwyB0 | 1.658 |
| 158 | WP_012461251.1 | 307D ,  384D ,  386E | 1bpm_0 | 2.176 |
| 159 | WP_012461252.1 | 282H ,  283D | 1occA0 | 1.74 |
| 160 | WP_012461260.1 | 671D ,  672E ,  673V ,  674K | 1f1zA1 | 1.813 |
| 161 | WP_012461270.1 | 194D ,  197D | 1mxgA0 | 1.472 |
| 162 | WP_012461272.1 | 67D ,  71T | 1n1zA0 | 1.464 |
| 163 | WP_012461273.1 | 576L ,  579N | 1ne9A2 | 1.886 |
| 164 | WP_012461274.1 | 412K ,  413S | 1jwyB0 | 1.857 |
| 165 | WP_012461304.1 | 464K ,  466S | 1jqvA0 | 1.859 |
| 166 | WP_012461310.1 | 272D ,  273D | 1auk_0 | 1.808 |
| 167 | WP_012461313.1 | 26K ,  27S | 1jwyB0 | 1.89 |
| 168 | WP_012461341.1 | 163L ,  166N | 1ne9A2 | 1.825 |
| 169 | WP_012461349.1 | 462K ,  463S | 1jwyB0 | 1.899 |
| 170 | WP_012461354.1 | 158K ,  160S | 1jqvA0 | 1.823 |
| 171 | WP_012461358.1 | 111H ,  115H ,  311D | 1ka2A0 | 1.751 |
| 172 | WP_012461365.1 | 205K ,  206S | 1jwyB0 | 1.513 |
| 173 | WP_012461378.1 | 71V ,  72D | 1k77A1 | 1.603 |
| 174 | WP_012461402.1 | 111D ,  115D | 1h1lB0 | 1.672 |
| 175 | WP_012461405.1 | 335V ,  336V | 1iruG0 | 1.942 |
| 176 | WP_012461406.1 | 107Q ,  110D | 1mfrA1 | 1.713 |
| 177 | WP_012461431.1 | 82T ,  83S | 1ezwA0 | 1.723 |
| 178 | WP_012461432.1 | 428K ,  429S | 1jwyB0 | 1.743 |
| 179 | WP_012461437.1 | 49K ,  50S | 1jwyB0 | 1.893 |
| 180 | WP_012461438.1 | 44V ,  45D | 1k77A1 | 1.897 |
| 181 | WP_012461442.1 | 22K ,  23S | 1jwyB0 | 1.87 |
| 182 | WP_012461452.1 | 73I ,  74D | 1k77A1 | 1.251 |
| 183 | WP_041621579.1 | 578D ,  579E | 1bwvA0 | 1.92 |
| 184 | WP_041621635.1 | 101T ,  102S | 1ezwA0 | 1.673 |
| 185 | WP_041621667.1 | 31T ,  32S | 1ezwA0 | 1.827 |
| 186 | WP_041621687.1 | 97K ,  101S | 1jqvA0 | 1.499 |
| 187 | WP_041621803.1 | 268D ,  284Q ,  285V ,  286T | 1f1zA1 | 2.011 |
| 188 | WP_045916150.1 | 16E ,  17D | 1grv_0 | 1.9 |
| 189 | WP_050731348.1 | 582K ,  583S | 1jwyB0 | 1.73 |
| 190 | WP_050731357.1 | 182K ,  183S | 1jwyB0 | 1.716 |
| 191 | WP_050731368.1 | 182K ,  183S | 1jwyB0 | 1.716 |
| 192 | WP_080503901.1 | 120E ,  122N | 1ehiA0 | 1.703 |
| 193 | WP_012462217.1 | 194D ,  198D | 1h1lB0 | 1.484 |

**Supplementary Table S2B. Details of interacting amino acid residues of with Ca^2+^.**

| **S.No.** | **Sequence ID** | **Binding Residues** | **Template** | **Score** |
| --- | --- | --- | --- | --- |
| 1 | WP_011945048.1 | 6V ,  50E | 1i40A3 | 1.286 |
| 2 | WP_012460728.1 | 394E ,  396L | 1mu5A1 | 1.863 |
| 3 | WP_012460755.1 | 45E ,  46Q ,  49E | 5stdA0 | 1.647 |
| 4 | WP_012460803.1 | 646D ,  649D ,  650E | 1je5B0 | 1.886 |
| 5 | WP_012460805.1 | 25D ,  26F | 1nyaA0 | 1.877 |
| 6 | WP_012460843.1 | 67D ,  69D | 1tnq_0 | 1.687 |
| 7 | WP_012460873.1 | 23S ,  24D ,  27E | 5stdA0 | 1.709 |
| 8 | WP_012460882.1 | 202D ,  203I | 1tnq_0 | 1.889 |
| 9 | WP_012460884.1 | 257E ,  307D ,  308D | 1h1vG3 | 1.891 |
| 10 | WP_012460897.1 | 84N ,  85D | 1e5nA0 | 1.453 |
| 11 | WP_012460901.1 | 33Q ,  34D | 1akl_4 | 1.486 |
| 12 | WP_012460938.1 | 462E ,  463L | 1nyaA2 | 1.899 |
| 13 | WP_012460950.1 | 268D ,  271Q | 1g42A2 | 1.791 |
| 14 | WP_012460952.1 | 287S ,  289V ,  291I | 1e8uB0 | 2.275 |
| 15 | WP_012460954.1 | 165S ,  167D ,  168F | 1n48A1 | 1.52 |
| 16 | WP_012460958.1 | 45E ,  48E | 1esl_1 | 1.878 |
| 17 | WP_012460959.1 | 257E ,  307D ,  308D | 1h1vG3 | 1.89 |
| 18 | WP_012460962.1 | 259E ,  309D ,  310D | 1h1vG3 | 1.892 |
| 19 | WP_012460964.1 | 18D ,  19E | 1je5B0 | 1.671 |
| 20 | WP_012460966.1 | 8D ,  59E | 1f7lA0 | 1.815 |
| 21 | WP_012460969.1 | 122E ,  124L | 1mu5A1 | 1.415 |
| 22 | WP_012460992.1 | 257E ,  307D ,  308D | 1h1vG3 | 1.89 |
| 23 | WP_012460998.1 | 198D ,  201D ,  202E | 1je5B0 | 1.531 |
| 24 | WP_012461008.1 | 119E ,  122E | 1de4C0 | 1.531 |
| 25 | WP_012461013.1 | 253E ,  303D ,  304D | 1h1vG3 | 1.892 |
| 26 | WP_012461022.1 | 67D ,  69D | 1tnq_0 | 1.689 |
| 27 | WP_012461051.1 | 276D ,  277Y | 1pk6B0 | 1.841 |
| 28 | WP_012461072.1 | 866N ,  869E | 1lw5D0 | 1.808 |
| 29 | WP_012461109.1 | 184Q ,  185D | 1akl_4 | 1.902 |
| 30 | WP_012461125.1 | 253E ,  303D ,  304D | 1h1vG3 | 1.89 |
| 31 | WP_012461164.1 | 51E ,  55D | 1otnA0 | 1.741 |
| 32 | WP_012461165.1 | 399D ,  402D | 4sbvC0 | 1.894 |
| 33 | WP_012461168.1 | 358N ,  359T ,  373D | 1lmjA0 | 1.809 |
| 34 | WP_012461219.1 | 425A ,  426N | 1nl2A3 | 1.691 |
| 35 | WP_012461223.1 | 35D ,  36T | 1sta_0 | 1.883 |
| 36 | WP_012461224.1 | 154T ,  155D | 1su4A1 | 1.844 |
| 37 | WP_012461243.1 | 232A ,  233N | 1nl2A3 | 1.788 |
| 38 | WP_012461248.1 | 136E ,  138L | 1mu5A1 | 1.757 |
| 39 | WP_012461251.1 | 543E ,  544D ,  547E | 5stdA0 | 1.954 |
| 40 | WP_012461273.1 | 876E ,  878L | 1mu5A1 | 1.872 |
| 41 | WP_012461274.1 | 197E ,  198R | 1j24A0 | 1.903 |
| 42 | WP_012461312.1 | 349E ,  353E | 1mj2B0 | 1.508 |
| 43 | WP_012461346.1 | 61N ,  62D | 1h3gA0 | 1.972 |
| 45 | WP_012461349.1 | 69D ,  70D | 1h1vG3 | 1.664 |
| 45 | WP_012461355.1 | 126E ,  128L | 1mu5A1 | 1.835 |
| 46 | WP_012461360.1 | 67S ,  68D | 1lwsA1 | 1.536 |
| 47 | WP_012461393.1 | 325G ,  326N | 1h0hA0 | 1.846 |
| 48 | WP_012461406.1 | 17D ,  19I | 1imeA0 | 1.926 |
| 49 | WP_012461445.1 | 373N ,  374N | 1cdg_0 | 1.538 |
| 50 | WP_012461450.1 | 98T ,  100S | 1clc_1 | 1.696 |
| 51 | WP_012461476.1 | 257E ,  307D ,  308D | 1h1vG3 | 1.894 |
| 52 | WP_012461492.1 | 335D ,  336D | 1jv2B0 | 1.747 |
| 53 | WP_012461493.1 | 346A ,  347N | 1nl2A3 | 1.857 |
| 54 | WP_012461512.1 | 87D ,  88K | 1n7uA0 | 1.692 |
| 55 | WP_012461525.1 | 154E ,  155D ,  158K | 5stdA0 | 1.548 |
| 56 | WP_012461532.1 | 273E ,  277E | 1mj2B0 | 1.995 |
| 57 | WP_012461533.1 | 277D ,  279D | 1h1vG3 | 1.78 |
| 58 | WP_012461596.1 | 405Q ,  406D | 1akl_4 | 1.688 |
| 59 | WP_012461600.1 | 144P ,  146I | 1sra_2 | 1.765 |
| 60 | WP_012461602.1 | 72E ,  74I | 1mu5A1 | 1.462 |
| 61 | WP_012461603.1 | 63G ,  64P | 1c9uB2 | 1.87 |
| 62 | WP_012461605.1 | 197D ,  198D | 1h1vG3 | 1.667 |
| 63 | WP_012461626.1 | 163D ,  164E | 1gr3A1 | 1.738 |
| 64 | WP_012461643.1 | 294E ,  296L | 1mu5A1 | 1.784 |
| 65 | WP_012461655.1 | 241D ,  242D ,  245E | 5stdA0 | 1.753 |
| 66 | WP_012461681.1 | 94E ,  97E | 1esl_1 | 1.796 |
| 67 | WP_012461713.1 | 379D ,  380I | 1tnq_0 | 1.874 |
| 68 | WP_012461727.1 | 244D ,  245Y | 1cb8A0 | 1.942 |
| 69 | WP_012461746.1 | 247D ,  248M | 1hj7A0 | 1.464 |
| 70 | WP_012461765.1 | 79D ,  80N | 1b09A1 | 1.74 |
| 71 | WP_012461769.1 | 27G ,  28P | 1c9uB2 | 1.827 |
| 72 | WP_012461828.1 | 149D ,  150K ,  151N | 1fi5A1 | 1.929 |
| 73 | WP_012461831.1 | 639D ,  640N | 1n2lA0 | 1.737 |
| 74 | WP_012461832.1 | 35D ,  36K | 1n7uA0 | 1.651 |
| 75 | WP_012461844.1 | 177D ,  178D | 1ia7A0 | 1.926 |
| 76 | WP_012461879.1 | 819A ,  820N | 1nl2A3 | 1.864 |
| 77 | WP_012461890.1 | 466D ,  468D ,  469F | 1n48A1 | 2.156 |
| 78 | WP_012461904.1 | 55L ,  56G ,  57Q | 1h0hA0 | 1.614 |
| 79 | WP_012461920.1 | 91T ,  92H | 1c7iA0 | 1.96 |
| 80 | WP_012461957.1 | 259E ,  309D ,  310D | 1h1vG3 | 1.889 |
| 81 | WP_012462008.1 | 218D ,  219N | 1alvA3 | 1.826 |
| 82 | WP_012462010.1 | 335N ,  336D | 1egiB0 | 1.734 |
| 83 | WP_012462032.1 | 23D ,  24T ,  106D | 1sta_0 | 2.243 |
| 84 | WP_012462050.1 | 627I ,  628G ,  629N | 1h0hA0 | 2.103 |
| 85 | WP_012462074.1 | 84L ,  85G ,  86Q | 1h0hA0 | 1.562 |
| 86 | WP_012462078.1 | 20D ,  21E | 1ayoB0 | 1.379 |
| 87 | WP_012462079.1 | 2N ,  3D | 1l6rA0 | 1.798 |
| 88 | WP_012462083.1 | 246D ,  247D | 1h1vG3 | 1.789 |
| 89 | WP_012462109.1 | 259E ,  262E | 1esl_1 | 1.778 |
| 90 | WP_012462134.1 | 253E ,  303D ,  304D | 1h1vG3 | 1.894 |
| 91 | WP_012462153.1 | 253E ,  303D ,  304D | 1h1vG3 | 1.891 |
| 92 | WP_012462217.1 | 143D ,  145E | 1q5cC4 | 1.74 |
| 93 | WP_012462223.1 | 209D ,  210A | 1oacB0 | 1.735 |
| 94 | WP_012462244.1 | 31D ,  35D | 1ika_0 | 1.535 |
| 95 | WP_012462252.1 | 151N ,  162N | 1e5jA1 | 1.792 |
| 96 | WP_012462261.1 | 253E ,  303D ,  304D | 1h1vG3 | 1.893 |
| 97 | WP_012462269.1 | 98I ,  99A | 1su4A0 | 1.585 |
| 98 | WP_012462271.1 | 115D ,  116S | 1nrwA0 | 1.864 |
| 99 | WP_012462286.1 | 150N ,  151D | 1izjA2 | 1.867 |
| 100 | WP_012462316.1 | 554E ,  557E | 1de4C0 | 1.795 |
| 101 | WP_012462318.1 | 101D ,  102E | 1obr_0 | 1.812 |
| 102 | WP_012462322.1 | 44D ,  45N | 1n2lA0 | 1.804 |
| 103 | WP_012462325.1 | 72E ,  74E | 1fbl_0 | 1.485 |
| 104 | WP_012462327.1 | 165E ,  166L | 1nyaA2 | 1.836 |
| 105 | WP_012462330.1 | 214E ,  216L | 1mu5A1 | 1.533 |
| 106 | WP_041621635.1 | 357A ,  358N | 1nl2A3 | 1.884 |
| 107 | WP_041621643.1 | 38E ,  41E | 1de4C0 | 1.801 |
| 108 | WP_041621700.1 | 61L ,  62G ,  63N | 1h0hA0 | 2.262 |
| 109 | WP_050731342.1 | 295D ,  296I | 1f2oA0 | 1.71 |
| 110 | WP_050731348.1 | 136D ,  137D ,  269Q | 1h1vG3 | 1.934 |
| 111 | WP_080503942.1 | 26D ,  27A | 1i82A2 | 1.843 |

**Supplementary Table S2C. Details of interacting amino acid residues of with Zn^2+^.**

| **S.No.** | **Sequence ID** | **Binding Residues** | **Template** | **Score** |
| --- | --- | --- | --- | --- |
| 1 | WP_012460730.1 | 98D ,  100H ,  102N | 1gl4A0 | 1.61 |
| 2 | WP_012460738.1 | 200E ,  204E | 1no5B2 | 1.659 |
| 3 | WP_012460740.1 | 45T ,  46H | 1qe3A0 | 1.359 |
| 4 | WP_012460742.1 | 120D ,  124E | 1f30A0 | 1.053 |
| 5 | WP_012460755.1 | 46Q ,  50E | 1jk0A0 | 1.812 |
| 6 | WP_012460797.1 | 161D ,  162H | 1qh3A1 | 1.687 |
| 7 | WP_012460949.1 | 263H ,  267H ,  293E | 1i1iP0 | 2.069 |
| 8 | WP_012460952.1 | 433C ,  436C ,  475C ,  478C | 1h3nA0 | 3.005 |
| 9 | WP_012460983.1 | 38C ,  41H ,  59C ,  62C | 1d0qA0 | 2.388 |
| 10 | WP_012460994.1 | 483C ,  485H ,  486C | 1tbn_1 | 1.865 |
| 11 | WP_012460998.1 | 64H ,  65E | 1k9zA6 | 1.791 |
| 12 | WP_012461051.1 | 18E ,  20H ,  203D | 1l0yA0 | 1.511 |
| 13 | WP_012461161.1 | 169I ,  179C | 1chc_0 | 1.24 |
| 14 | WP_012461220.1 | 19E ,  22E | 1r4vA0 | 1.503 |
| 15 | WP_012461222.1 | 47A ,  49A ,  50C ,  118H ,  119C | 1jm7A1 | 1.516 |
| 16 | WP_012461232.1 | 145D ,  170D | 1k9zA2 | 1.465 |
| 17 | WP_012461235.1 | 87D ,  88H | 1qh3A1 | 1.663 |
| 18 | WP_012461243.1 | 33H ,  34E | 1k9zA6 | 1.799 |
| 19 | WP_012461246.1 | 222H ,  225H | 1qr2A0 | 1.328 |
| 20 | WP_012461251.1 | 302K ,  307D ,  325D ,  386E | 1gytA0 | 3.174 |
| 21 | WP_012461260.1 | 357D ,  358H | 1smlA0 | 1.527 |
| 22 | WP_012461312.1 | 15A ,  19C | 1eucB0 | 1.804 |
| 23 | WP_012461354.1 | 182K ,  185R | 1l9hA1 | 1.757 |
| 24 | WP_012461358.1 | 111H ,  115H ,  311D | 1pwuA0 | 1.831 |
| 25 | WP_012461377.1 | 39E ,  42E | 1r4vA0 | 1.798 |
| 26 | WP_012461378.1 | 178H ,  179D | 1f0jA0 | 1.5 |
| 27 | WP_012461379.1 | 366C ,  369C ,  371S ,  384C ,  387C | 1m2gA0 | 3.263 |
| 28 | WP_012461388.1 | 206D ,  207H | 1qh3A1 | 1.852 |
| 29 | WP_012461389.1 | 29C ,  216C ,  241H ,  245E | 1li7A0 | 2.646 |
| 30 | WP_012461403.1 | 271E ,  275E | 1no5B2 | 1.734 |
| 31 | WP_012461437.1 | 64R ,  70G | 1l9hA1 | 1.162 |
| 32 | WP_012461451.1 | 200H ,  204D | 1hfeS0 | 1.529 |
| 33 | WP_012461601.1 | 49E ,  53E | 1q9uB4 | 1.385 |
| 34 | WP_012461626.1 | 128C ,  131C ,  148C ,  151C | 1jzqA0 | 2.196 |
| 35 | WP_012461643.1 | 9D ,  10D | 1bawA0 | 1.685 |
| 36 | WP_012461719.1 | 47T ,  48H | 1qe3A0 | 1.655 |
| 37 | WP_012461774.1 | 107D ,  109D | 1f35A3 | 1.299 |
| 38 | WP_012461804.1 | 17E ,  20H | 1b71A0 | 1.728 |
| 39 | WP_012461822.1 | 300H ,  301E | 1k9zA6 | 1.535 |
| 40 | WP_012461831.1 | 598C ,  601C ,  619C ,  625C | 1j8fA0 | 3.031 |
| 41 | WP_012461836.1 | 51Q ,  103C ,  145H ,  149H | 1ix1A0 | 3.364 |
| 42 | WP_012461846.1 | 351D ,  352D | 1bawA0 | 1.914 |
| 43 | WP_012461854.1 | 58D ,  62Q | 1f30A0 | 1.369 |
| 45 | WP_012461858.1 | 391D ,  394D | 1mxdA1 | 1.534 |
| 45 | WP_012461868.1 | 100D ,  103L | 1taq_0 | 1.704 |
| 46 | WP_012461876.1 | 70T ,  71H | 1qe3A0 | 1.365 |
| 47 | WP_012461879.1 | 280E ,  284D | 1a7w_0 | 1.762 |
| 48 | WP_012461890.1 | 447E ,  451D | 1a7w_0 | 1.686 |
| 49 | WP_012461905.1 | 755C ,  757A ,  758C ,  778C ,  781C | 1gaxA1 | 2.904 |
| 50 | WP_012461920.1 | 365M ,  366H | 1fioA0 | 1.803 |
| 51 | WP_012461928.1 | 50S ,  52H | 1iqbA0 | 1.787 |
| 52 | WP_012461981.1 | 252H ,  253E | 1k9zA6 | 1.512 |
| 53 | WP_012462043.1 | 418H ,  419E | 1k9zA6 | 1.811 |
| 54 | WP_012462079.1 | 269D ,  270H | 1smlA0 | 1.485 |
| 55 | WP_012462083.1 | 106H ,  109N | 1cvrA1 | 1.479 |
| 56 | WP_012462089.1 | 18E ,  21E | 3caoA3 | 1.466 |
| 57 | WP_012462162.1 | 69C ,  82C ,  85C ,  88C | 1njgA0 | 2.685 |
| 58 | WP_012462166.1 | 130D ,  131D | 1bawA0 | 1.818 |
| 59 | WP_012462219.1 | 58C ,  61C ,  63N ,  70C ,  73C | 4gatA0 | 2.772 |
| 60 | WP_012462271.1 | 32E ,  43D | 1f30A0 | 1.389 |
| 61 | WP_012462272.1 | 64S ,  65R | 1hbmD0 | 1.891 |
| 62 | WP_012462273.1 | 6Q ,  8E | 1a6f_0 | 1.398 |
| 63 | WP_012462274.1 | 212E ,  216H | 1lbcB1 | 1.681 |
| 64 | WP_012462285.1 | 35D ,  36H | 1qh3A1 | 1.809 |
| 65 | WP_012462316.1 | 396D ,  407D ,  409T ,  517E | 1pv9A1 | 3.312 |
| 66 | WP_012462317.1 | 422C ,  425C ,  427Q ,  440C ,  446C | 1irn_0 | 2.032 |
| 67 | WP_012462318.1 | 50H ,  80C ,  83C | 1rb7A0 | 2.411 |
| 68 | WP_012462319.1 | 267H ,  268E | 1k9zA6 | 1.79 |
| 69 | WP_012462322.1 | 194C ,  201N ,  203C ,  208S | 1eucB0 | 1.476 |
| 70 | WP_012462327.1 | 147H ,  151D | 1ah7_0 | 1.654 |
| 71 | WP_041621607.1 | 160E ,  163D | 1r4vA0 | 1.242 |
| 72 | WP_041621635.1 | 284H ,  285E | 1k9zA6 | 1.712 |
| 73 | WP_041621781.1 | 96T ,  97H | 1qe3A0 | 1.872 |
| 74 | WP_045916150.1 | 437H ,  438E | 1k9zA6 | 1.702 |
| 75 | WP_050731342.1 | 91D ,  92H ,  177D ,  404H | 1qh3A1 | 2.152 |
| 76 | WP_050731348.1 | 34D ,  136D ,  139I ,  159D | 1taq_0 | 2.294 |

**Supplementary Table S2D. Details of interacting amino acid residues of with Mn^2+^.**

| **S.No**. | **Sequence ID** | **Binding Residues** | **Template** | **Score** |
| --- | --- | --- | --- | --- |
| 1 | WP_012460866.1 | 165E ,  169E | 1mqwA1 | 1.531 |
| 2 | WP_012461445.1 | 146R ,  147E | 1mqwA2 | 1.808 |
| 3 | WP_012461890.1 | 104E ,  108E | 1hx3B0 | 1.504 |
| 4 | WP_012461942.1 | 27D ,  30E | 1g15A0 | 1.65 |
| 5 | WP_050731388.1 | 67D ,  98E ,  101H ,  192E | 1jprB0 | 3.131 |
| 6 | WP_012460730.1 | 153R ,  154E | 1mqwA2 | 1.716 |
| 7 | WP_012460848.1 | 165E ,  169E | 1mqwA1 | 1.532 |
| 8 | WP_012460872.1 | 172E ,  176E | 1mnp_0 | 1.79 |
| 9 | WP_012460882.1 | 202D ,  203I | 1n1hA0 | 1.786 |
| 10 | WP_012460897.1 | 85D ,  121H | 1gv3A0 | 1.401 |
| 11 | WP_012461036.1 | 21D ,  22T | 1gx6A0 | 1.529 |
| 12 | WP_012461051.1 | 330D ,  332D ,  333I | 1n1hA0 | 2.018 |
| 13 | WP_012461119.1 | 83D ,  87D ,  88T | 1gx6A0 | 1.258 |
| 14 | WP_012461377.1 | 4D ,  201E ,  202T | 1m0dA0 | 2.039 |
| 15 | WP_012461498.1 | 52K ,  53E | 1mqwA2 | 1.24 |
| 16 | WP_012461546.1 | 219D ,  234H | 1g5bB0 | 1.5 |
| 17 | WP_012461589.1 | 161E ,  165E | 1mqwA1 | 1.532 |
| 18 | WP_012461643.1 | 291D ,  294E | 1f3wA0 | 1.35 |
| 19 | WP_012461761.1 | 22D ,  25E | 1g15A0 | 1.651 |
| 20 | WP_012461775.1 | 42E ,  66E ,  73E | 1f1hA0 | 2.38 |
| 21 | WP_012461854.1 | 198D ,  199G | 1a6q_1 | 1.523 |
| 22 | WP_012461879.1 | 488D ,  492E | 1qmgA0 | 1.513 |
| 23 | WP_012461924.1 | 98D ,  151D ,  175H , 176H ,  248D | 1ir6A0 | 2.345 |
| 24 | WP_012462037.1 | 404D ,  406D ,  407I | 1n1hA0 | 1.684 |
| 25 | WP_012462083.1 | 24D ,  28H | 1nhxA0 | 1.674 |
| 26 | WP_012462089.1 | 296E ,  297H | 1ipsA1 | 1.728 |
| 27 | WP_012462180.1 | 165E ,  169E | 1mqwA1 | 1.532 |
| 28 | WP_012462223.1 | 346D ,  349D | 1didA0 | 1.506 |
| 29 | WP_012462327.1 | 18K ,  20D ,  21T | 1gx6A0 | 1.206 |
| 30 | WP_050731342.1 | 295D ,  296I | 1n1hA0 | 1.675 |
| 31 | WP_012460749.1 | 44E ,  48E | 1hx3B0 | 1.386 |
| 32 | WP_012460805.1 | 118E ,  121H | 1kgpA1 | 1.826 |
| 33 | WP_012460950.1 | 46D ,  49E | 1g15A0 | 1.486 |
| 34 | WP_012461247.1 | 33E ,  161Q | 1m6vA1 | 1.668 |
| 35 | WP_012461272.1 | 38H ,  91H ,  173D , 177H | 1gv3A0 | 2.957 |
| 36 | WP_012461298.1 | 180E ,  184E | 1mnp_0 | 1.79 |
| 37 | WP_012461304.1 | 141K ,  142F ,  143D | 1ksiA0 | 1.642 |
| 38 | WP_012461360.1 | 8D ,  10E ,  157D | 1j53A0 | 2.034 |
| 39 | WP_012461371.1 | 27D ,  30E | 1g15A0 | 1.652 |
| 40 | WP_012461403.1 | 312E ,  315H | 1bfrA0 | 1.286 |
| 41 | WP_012461472.1 | 31D ,  34E | 1g15A0 | 1.651 |
| 42 | WP_012461499.1 | 248D ,  249F ,  250D | 1ksiA0 | 2.114 |
| 43 | WP_012461501.1 | 374D ,  376D ,  377M | 1n1hA0 | 1.637 |
| 45 | WP_012461549.1 | 86D ,  88D | 1g8oA0 | 1.309 |
| 45 | WP_012461612.1 | 79T ,  81V | 1zqlA1 | 1.387 |
| 46 | WP_012461616.1 | 75D ,  76S | 1gx6A0 | 1.159 |
| 47 | WP_012461727.1 | 28D ,  29T | 1gx6A0 | 1.734 |
| 48 | WP_012461744.1 | 27D ,  30E | 1g15A0 | 1.653 |
| 49 | WP_012461746.1 | 148D ,  150N ,  223H | 1ncy_1 | 1.359 |
| 50 | WP_012461769.1 | 19D ,  57E ,  79D | 1g15A0 | 1.623 |
| 51 | WP_012462050.1 | 641D ,  642I | 1n1hA0 | 1.836 |
| 52 | WP_012462073.1 | 215E ,  219E | 1mqwA1 | 1.531 |
| 53 | WP_012462078.1 | 58R ,  59E | 1mqwA2 | 1.849 |
| 54 | WP_012462097.1 | 29Q ,  30H | 1ipsA1 | 1.492 |
| 55 | WP_012462166.1 | 243D ,  244G | 1a6q_1 | 1.212 |
| 56 | WP_012462208.1 | 349K ,  351L | 1zqlA2 | 1.444 |
| 57 | WP_012462260.1 | 180E ,  184E | 1mnp_0 | 1.791 |
| 58 | WP_012462271.1 | 140E ,  141H | 1ipsA1 | 1.731 |
| 59 | WP_012462292.1 | 105E ,  109E | 1mqwA1 | 1.431 |
| 60 | WP_012462309.1 | 27D ,  30E | 1g15A0 | 1.65 |
| 61 | WP_012462316.1 | 407D ,  470H ,  503E ,  517E | 1n51A0 | 2.611 |
| 62 | WP_041621594.1 | 180E ,  184E | 1mnp_0 | 1.791 |
| 63 | WP_041621747.1 | 106D ,  169H ,  203E ,  234E | 1n51A0 | 2.322 |
| 64 | WP_041621803.1 | 226D ,  227I | 1ksiA0 | 1.66 |
| 65 | WP_050731348.1 | 476E ,  477H | 1ipsA1 | 1.97 |

**Supplementary Table S2E. Details of interacting amino acid residues of with Fe^2+^.**

| **S.No**. | **Sequence ID** | **Binding Residues** | **Template** | **Score** |
| --- | --- | --- | --- | --- |
| 1 | WP_011944977.1 | 48C ,  49C ,  85T ,  113C | 1vziA1 | 1.1 |
| 2 | WP_012460738.1 | 185D ,  187D ,  188D | 4b2oA1 | 1.012 |
| 3 | WP_012460772.1 | 144S ,  147H ,  180E | 1fyzA2 | 0.96 |
| 4 | WP_012460808.1 | 191N ,  194H | 1kgoA2 | 1.17 |
| 5 | WP_012460882.1 | 142D ,  178E | 3ak9A1 | 1.354 |
| 6 | WP_012460919.1 | 8H ,  12H | 3aqjA1 | 1.132 |
| 7 | WP_012460923.1 | 136T ,  137H ,  143D | 2vw8A1 | 1.087 |
| 8 | WP_012460963.1 | 8D ,  12Q | 2z90A1 | 1.119 |
| 9 | WP_012461000.1 | 61H ,  65E | 3e1mA3 | 1.075 |
| 10 | WP_012461063.1 | 147D ,  151E | 3ak9A1 | 1.81 |
| 11 | WP_012461076.1 | 353H ,  357D | 3e1mA3 | 1.235 |
| 12 | WP_012461170.1 | 33D ,  35H ,  143E | 1yuxA1 | 1.437 |
| 13 | WP_012461218.1 | 30E ,  33H | 1biqA1 | 0.923 |
| 14 | WP_012461222.1 | 97D ,  99E ,  100Q | 4b2oA1 | 0.978 |
| 15 | WP_012461223.1 | 151D ,  153N ,  154N | 4b2oA1 | 1.212 |
| 16 | WP_012461224.1 | 78H ,  79Y | 2pt2A1 | 1.388 |
| 17 | WP_012461272.1 | 38H ,  43H | 1jnqA1 | 1.243 |
| 18 | WP_012461278.1 | 138N ,  193E ,  196H | 3e1mA2 | 1.388 |
| 19 | WP_012461332.1 | 76H ,  80D | 2bq8X2 | 0.894 |
| 20 | WP_012461337.1 | 40Y ,  41F | 2pt2A1 | 1.313 |
| 21 | WP_012461346.1 | 32F ,  55H ,  101E | 2ehzA1 | 1.131 |
| 22 | WP_012461358.1 | 111H ,  115H ,  135S , 311D | 3venA1 | 1.518 |
| 23 | WP_012461452.1 | 17E ,  21E ,  24H ,  50D | 3e1mA1 | 1.258 |
| 24 | WP_012461483.1 | 113D ,  115D ,  116N | 4b2oA1 | 1.304 |
| 25 | WP_012461499.1 | 80Q ,  82H ,  107F , 334E | 1fyzA2 | 0.842 |
| 26 | WP_012461500.1 | 156D ,  160E | 3ak9A1 | 1.325 |
| 27 | WP_012461501.1 | 410H ,  414D | 3e1mA3 | 1.347 |
| 28 | WP_012461557.1 | 19H ,  23E ,  27E ,  29Q | 4b2oA1 | 1.041 |
| 29 | WP_012461569.1 | 252H ,  254H | 2zylA1 | 1.27 |
| 30 | WP_012461701.1 | 326D ,  330E | 3ak9A1 | 1.523 |
| 31 | WP_012461772.1 | 36D ,  38E ,  39K | 4b2oA1 | 1.115 |
| 32 | WP_012461786.1 | 168D ,  172E | 2z90A1 | 1.474 |
| 33 | WP_012461818.1 | 178E ,  181N | 1oqbA1 | 0.749 |
| 34 | WP_012461836.1 | 126Y ,  127Y | 2pt2A1 | 1.712 |
| 35 | WP_012461883.1 | 257D ,  259H | 2bl4A1 | 0.984 |
| 36 | WP_012461889.1 | 171D ,  194E | 3ak9A1 | 1.229 |
| 37 | WP_012461905.1 | 755C ,  758C ,  778C ,  781C | 1fhmA1 | 2.141 |
| 38 | WP_012461990.1 | 37D ,  112D ,  114K | 3mz6A1 | 0.955 |
| 39 | WP_012462008.1 | 36H ,  82D | 4hr4A1 | 0.993 |
| 40 | WP_012462035.1 | 273E ,  277H ,  282N , 306E | 1fyzA2 | 1.158 |
| 41 | WP_012462050.1 | 297D ,  301E | 2z90A1 | 1.498 |
| 42 | WP_012462160.1 | 80D ,  81N | 4b2oA1 | 1.376 |
| 43 | WP_012462195.1 | 321D ,  325E | 2z90A1 | 1.419 |
| 45 | WP_012462274.1 | 434D ,  438E | 3ak9A1 | 1.298 |
| 45 | WP_012462321.1 | 92D ,  96E | 3ak9A1 | 1.719 |
| 46 | WP_012462322.1 | 205Y ,  206F | 2pt2A1 | 1.229 |
| 47 | WP_012462330.1 | 51D ,  53E ,  54N | 4b2oA1 | 1.449 |
| 48 | WP_041621568.1 | 107E ,  155D | 3e1mA1 | 0.881 |
| 49 | WP_041621787.1 | 126C ,  130G ,  145C , 147C | 1h79A1 | 1.308 |
| 50 | WP_045914353.1 | 20H ,  24G ,  73H ,  80H | 4g32A1 | 1.125 |
| 51 | WP_050731388.1 | 98E ,  158E ,  192E ,  195H | 1w69A2 | 2.589 |
| 52 | WP_012461967.1 | 139D ,  143E | 3ak9A1 | 1.322 |

**Supplementary Table S2F. Details of interacting amino acid residues of with Fe^3+^.**

| **S.No.** | **Sequence ID** | **Binding Residues** | **Template** | **Score** |
| --- | --- | --- | --- | --- |
| 1 | WP_011944977.1 | 48C ,  49C ,  85T ,  113C | 1dfx_0 | 1.134 |
| 2 | WP_012460738.1 | 109E ,  112Q | 1oquC4 | 1.195 |
| 3 | WP_012460772.1 | 144S ,  147H ,  180E | 1mmoD0 | 1.306 |
| 4 | WP_012460808.1 | 288E ,  291E | 1oquC4 | 1.938 |
| 5 | WP_012460882.1 | 260D ,  264E | 1qgh_2 | 1.917 |
| 6 | WP_012460919.1 | 237E ,  240E | 1oquC4 | 1.347 |
| 7 | WP_012460923.1 | 109H ,  113H ,  143D | 1lnbE0 | 0.959 |
| 8 | WP_012460963.1 | 159E ,  162D | 1oquC4 | 1.27 |
| 9 | WP_012461000.1 | 27H ,  61H ,  62C | 1do6A0 | 1.291 |
| 10 | WP_012461063.1 | 260E ,  263E | 1oquC4 | 1.822 |
| 11 | WP_012461076.1 | 339E ,  342E | 1oquC4 | 1.49 |
| 12 | WP_012461170.1 | 114Q ,  117E | 1oquC4 | 1.259 |
| 13 | WP_012461218.1 | 30E ,  33H ,  44E | 1oq4A1 | 0.983 |
| 14 | WP_012461222.1 | 50C ,  119C | 1frfL0 | 1.258 |
| 15 | WP_012461223.1 | 66E ,  69E | 1oquC4 | 1.665 |
| 16 | WP_012461224.1 | 59E ,  62E | 1oquC4 | 1.922 |
| 17 | WP_012461272.1 | 38H ,  43H | 1lnbE0 | 1.428 |
| 18 | WP_012461278.1 | 138N ,  193E ,  196H | 1nf6F0 | 1.184 |
| 19 | WP_012461332.1 | 132E ,  135K | 1oquC4 | 1.091 |
| 20 | WP_012461337.1 | 40Y ,  41F | 1d9yA0 | 1.217 |
| 21 | WP_012461346.1 | 32F ,  55H ,  101E | 1q0cA0 | 0.916 |
| 22 | WP_012461358.1 | 111H ,  115H ,  311D | 1lnbE0 | 1.563 |
| 23 | WP_012461452.1 | 11Y ,  12F | 1d9yA0 | 1.286 |
| 24 | WP_012461483.1 | 80Q ,  83H | 1xsm_0 | 1.459 |
| 25 | WP_012461499.1 | 145E ,  148E | 1oquC4 | 1.424 |
| 26 | WP_012461500.1 | 156D ,  160E | 1qgh_9 | 1.523 |
| 27 | WP_012461501.1 | 348C ,  351C | 1ohvA0 | 0.984 |
| 28 | WP_012461557.1 | 20E ,  23E | 1oquC4 | 1.366 |
| 29 | WP_012461569.1 | 346H ,  350K | 1nmoA0 | 1.179 |
| 30 | WP_012461701.1 | 345D ,  349E | 1qgh_2 | 1.538 |
| 31 | WP_012461772.1 | 20V ,  21C ,  43C ,  46C | 1dfx_0 | 1.035 |
| 32 | WP_012461786.1 | 236D ,  240D | 1qgh_9 | 1.297 |
| 33 | WP_012461818.1 | 290C ,  293C ,  419C , 422C | 1b71A0 | 1.991 |
| 34 | WP_012461836.1 | 33D ,  37E | 1qgh_9 | 1.535 |
| 35 | WP_012461883.1 | 244F ,  245Y | 1d9yA0 | 1.355 |
| 36 | WP_012461889.1 | 111E ,  114E | 1oquC4 | 1.396 |
| 37 | WP_012461905.1 | 757A ,  758C ,  778C ,  781C | 1dfx_0 | 1.87 |
| 38 | WP_012461990.1 | 126Y ,  147Y | 1d9yA0 | 1.234 |
| 39 | WP_012462008.1 | 160E ,  164H | 1gy9A1 | 1.516 |
| 40 | WP_012462035.1 | 405H ,  409E | 1nmoA0 | 1.376 |
| 41 | WP_012462050.1 | 297D ,  301E | 1qgh_7 | 1.524 |
| 42 | WP_012462160.1 | 161D ,  165K | 1qgh_8 | 1.235 |
| 43 | WP_012462195.1 | 322E ,  325E | 1oquC4 | 1.505 |
| 45 | WP_012462274.1 | 132D ,  136S ,  216H | 1qgh_1 | 1.388 |
| 45 | WP_012462321.1 | 92D ,  96E | 1qgh_1 | 1.501 |
| 46 | WP_012462322.1 | 57Y ,  58Y ,  71N | 1d9yA0 | 1.428 |
| 47 | WP_012462330.1 | 176C ,  177C ,  237T , 238C | 1dfx_0 | 1.101 |
| 48 | WP_041621568.1 | 23Y ,  24F | 1d9yA0 | 1.251 |
| 49 | WP_041621787.1 | 126C ,  145C | 1ndoA2 | 1.521 |
| 50 | WP_045914353.1 | 20H ,  24G ,  73H ,  80H | 1hu9A0 | 1.328 |
| 51 | WP_050731388.1 | 98E ,  158E ,  192E ,  195H | 1xsm_0 | 2.632 |
| 52 | WP_012461967.1 | 23N ,  26E | 1qgh_8 | 0.988 |

**Supplementary Table S2G. Details of interacting amino acid residues of with Cd^2+^.**

| **S.No.** | **Sequence ID** | **Binding Residues** | **Template** | **Score** |
| --- | --- | --- | --- | --- |
| 1 | WP_012461022.1 | 54Q ,  55E ,  58E | 4evdA8 | 1.907 |
| 2 | WP_012461052.1 | 346N ,  347E ,  350E | 4evdA8 | 1.499 |
| 3 | WP_012461358.1 | 111H ,  115H ,  311D | 1kufA1 | 1.467 |
| 4 | WP_012462008.1 | 160E ,  161E ,  164H | 4evdA8 | 1.697 |
| 5 | WP_012462109.1 | 258Q ,  259E ,  262E | 4evdA8 | 1.943 |
| 6 | WP_012460749.1 | 52D ,  62C | 2gj2A1 | 1.369 |
| 7 | WP_012460843.1 | 54Q ,  55E ,  58E | 4evdA8 | 1.907 |
| 8 | WP_012460938.1 | 365Q ,  366E ,  369E | 4evdA8 | 1.962 |
| 9 | WP_012461080.1 | 57Q ,  58E ,  61E | 4evdA8 | 1.999 |
| 10 | WP_012461170.1 | 142E ,  143E ,  146N | 4evdA8 | 1.515 |
| 11 | WP_012461290.1 | 161Q ,  162E ,  165E | 4evdA8 | 1.948 |
| 12 | WP_012461346.1 | 121D ,  126H | 2ev0A1 | 1.388 |
| 13 | WP_012461402.1 | 234D ,  235E ,  238E | 4evdA8 | 1.946 |
| 14 | WP_012461445.1 | 307E ,  311E | 1h96A4 | 1.496 |
| 15 | WP_012461681.1 | 161E ,  162E ,  165E | 4evdA8 | 2.644 |
| 16 | WP_012461832.1 | 295E ,  299H | 4evdA4 | 1.426 |
| 17 | WP_012461884.1 | 215E ,  216Q ,  219Q | 4evdA8 | 1.451 |
| 18 | WP_012462038.1 | 110E ,  113E | 4evdAf | 1.741 |
| 19 | WP_012462074.1 | 54Q ,  55E ,  58K | 4evdA8 | 1.422 |
| 20 | WP_012462214.1 | 139E ,  144E | 1gynA2 | 1.113 |
| 21 | WP_012462244.1 | 159E ,  160E ,  163E | 4evdA8 | 2.272 |
| 22 | WP_012462282.1 | 482G ,  507G ,  509Y , 546A | 1m8rA1 | 1.292 |
| 23 | WP_012462327.1 | 130E ,  131N ,  134E | 4evdA8 | 1.6 |
| 24 | WP_041621643.1 | 38E ,  39Q ,  42E | 4evdA8 | 2.131 |

**Supplementary Table S2H. Details of interacting amino acid residues of with Ni^2+^.**

| **S.No.** | **Sequence ID** | **Binding Residues** | **Template** | **Score** |
| --- | --- | --- | --- | --- |
| 1 | WP_012460749.1 | 72H ,  76T | 3gw7A1 | 0.702 |
| 2 | WP_012460966.1 | 77D ,  96A | 3de9A3 | 1.139 |
| 3 | WP_012461080.1 | 282Y ,  286M ,  317E | 2y39A1 | 1.02 |
| 4 | WP_012461184.1 | 460H ,  461E | 3skdA1 | 1.193 |
| 5 | WP_012461304.1 | 296K ,  297H | 2qq9A2 | 1.25 |
| 6 | WP_012461341.1 | 77I ,  79H ,  171H | 3gw7A3 | 0.667 |
| 7 | WP_012461377.1 | 130D ,  132E ,  133H | 2qq9A2 | 1.585 |
| 8 | WP_012461438.1 | 140V ,  141G ,  142C | 1ru3A2 | 1.103 |
| 9 | WP_012461600.1 | 252E ,  255H | 3kbnA1 | 1.349 |
| 10 | WP_012461640.1 | 127H ,  131H | 3de9A2 | 1.497 |
| 11 | WP_012461666.1 | 465H ,  469L | 2y39A1 | 1.108 |
| 12 | WP_012461726.1 | 211E ,  212H | 2qq9A2 | 1.414 |
| 13 | WP_012461836.1 | 103C ,  145H ,  149H | 2w3tA1 | 2.549 |
| 14 | WP_012462074.1 | 72E ,  76N | 3mgqD1 | 0.687 |
| 15 | WP_012462109.1 | 290Q ,  291H | 4m58A1 | 0.881 |
| 16 | WP_012462277.1 | 566H ,  567D | 3skdA1 | 1.419 |
| 17 | WP_041621803.1 | 84H ,  85N | 3skdA1 | 1.252 |

**Supplementary Table S2I. Details of interacting amino acid residues of with Co^2+^.**

| **S.No.** | **Sequence ID** | **Binding Residues** | **Template** | **Score** |
| --- | --- | --- | --- | --- |
| 1 | WP_012461098.1 | 56D ,  60E | 2xjmA1 | 1.452 |
| 2 | WP_012461260.1 | 499D ,  503E | 2xjmA1 | 1.493 |
| 3 | WP_012461272.1 | 38H ,  43H | 4ekdA1 | 1.378 |
| 4 | WP_012461349.1 | 356E ,  359E | 3ka4A5 | 1.433 |
| 5 | WP_012461403.1 | 72H ,  105D ,  168E | 2f7vA1 | 1.88 |
| 6 | WP_012461546.1 | 164H ,  166E | 3pjlA1 | 1.377 |
| 7 | WP_012461626.1 | 128C ,  131C ,  148C ,  151C | 1r0hA1 | 1.428 |
| 8 | WP_012461836.1 | 103C ,  145H ,  149H | 1rl4A1 | 2.401 |
| 9 | WP_012461949.1 | 348M ,  349H | 2uzpA1 | 1.495 |
| 10 | WP_012462078.1 | 59E ,  63D | 4b7bA4 | 1.366 |
| 11 | WP_012462316.1 | 396D ,  407D ,  408I ,  409T ,  517E | 1wn1A2 | 3.508 |
| 12 | WP_041621747.1 | 106D ,  169H ,  203E ,  234E | 4iu6A1 | 2.659 |
| 13 | WP_050731388.1 | 98E ,  158E ,  192E ,  195H | 1h0nA2 | 2.771 |

**Supplementary Table S2J. Details of interacting amino acid residues of with Cu^+^.**

| **S.No.** | **Sequence ID** | **Binding Residues** | **Template** | **Score** |
| --- | --- | --- | --- | --- |
| 1 | WP_012461170.1 | 329C ,  333C | 5fjeAB | 1.715 |
| 2 | WP_012461278.1 | 51V ,  55C | 5fjeA1 | 1.434 |
| 3 | WP_012461444.1 | 42C ,  44Q | 3fyeB0 | 1.083 |
| 4 | WP_012461525.1 | 84Y ,  87F ,  90C ,  94C,  181H | 2gqmA0 | 1.659 |
| 5 | WP_012461719.1 | 39H ,  48H | 4bz4A0 | 1.219 |
| 6 | WP_012461722.1 | 251H ,  300H ,  301H | 3fyeA0 | 2.126 |
| 7 | WP_012461806.1 | 110H ,  144H ,  146M | 3t0uA0 | 0.982 |

**Supplementary Table S2K. Details of interacting amino acid residues of with Cu^2+^.**

| **S.No.** | **Sequence ID** | **Binding Residues** | **Template** | **Score** |
| --- | --- | --- | --- | --- |
| 1 | WP_012461170.1 | 33D ,  34K | 1ukuA0 | 1.423 |
| 2 | WP_012461278.1 | 238G ,  239H | 1a8vB0 | 1.685 |
| 3 | WP_012461444.1 | 46D ,  47K | 1ukuA0 | 1.746 |
| 4 | WP_012461525.1 | 170S ,  171D | 1tho_0 | 1.525 |
| 5 | WP_012461719.1 | 98H ,  111H ,  113H | 1fsrA0 | 1.405 |
| 6 | WP_012461722.1 | 251H ,  300H ,  301H | 1m56A0 | 2.544 |
| 7 | WP_012461806.1 | 110H ,  144H ,  146M | 1n9eA0 | 1.196 |
| 8 | WP_012462325.1 | 18S ,  19K | 1ukuA0 | 0.777 |

**Supplementary Table S3A. Functional classification of 321 predicted MBPs. The MBPs are classified in to nine broad classes metabolism, gene expressiona and regulation, transport, post translational modification, cell signaling, protein folding, proteolysis, stress response regulator and antimicrobial resistance.**

| **S. No.** | **Sequence ID** | **Metal Bound** | **Subcellular localization** | **Function domain** | **Probable function in bacteria** | **Broad class** | **Reference** |
| --- | --- | --- | --- | --- | --- | --- | --- |
| 1 | WP_012461036.1 | Mn | Cytoplasm | HD domain | Nucleotide metabolism and signal transduction | Cell signaling | (Huynh et al., 2015) |
| 2 | WP_012462309.1 | Mn | Cytoplasm | HD domain | Nucleotide metabolism and signal transduction | Cell signaling | (Huynh et al., 2015) |
| 3 | WP_012461744.1 | Mn | Cytoplasm | HD domain | Nucleotide metabolism and signal transduction | Cell signaling | (Huynh et al., 2015) |
| 4 | WP_012461298.1 | Mn | Cytoplasm | HD domain | Nucleotide metabolism and signal transduction | Cell signaling | (Huynh et al., 2015) |
| 5 | WP_012461589.1 | Mn | Cytoplasm | HD domain | Nucleotide metabolism and signal transduction | Cell signaling | (Huynh et al., 2015) |
| 6 | WP_012462254.1 | Mg | Cytoplasm | HD domain | Nucleotide metabolism and signal transduction | Cell signaling | (Huynh et al., 2015) |
| 7 | WP_012461942.1 | Mn | Cytoplasm | HD domain | Nucleotide metabolism and signal transduction | Cell signaling | (Huynh et al., 2015) |
| 8 | WP_012461761.1 | Mn | Cytoplasm | HD domain | Nucleotide metabolism and signal transduction | Cell signaling | (Huynh et al., 2015) |
| 9 | WP_012462292.1 | Mn | Cytoplasm | HD domain | Nucleotide metabolism and signal transduction | Cell signaling | (Huynh et al., 2015) |
| 10 | WP_012461371.1 | Mn | Cytoplasm | HD domain | Nucleotide metabolism and signal transduction | Cell signaling | (Huynh et al., 2015) |
| 11 | WP_012462260.1 | Mn | Cytoplasm | HD domain | Nucleotide metabolism and signal transduction | Cell signaling | (Huynh et al., 2015) |
| 12 | WP_012460872.1 | Mn | Cytoplasm | HD domain | Nucleotide metabolism and signal transduction | Cell signaling | (Huynh et al., 2015) |
| 13 | WP_041621594.1 | Mn | Cytoplasm | HD domain | Nucleotide metabolism and signal transduction | Cell signaling | (Huynh et al., 2015) |
| 14 | WP_012460866.1 | Mn | Cytoplasm | HD domain | Nucleotide metabolism and signal transduction | Cell signaling | (Huynh et al., 2015) |
| 15 | WP_012460848.1 | Mn | Cytoplasm | HD domain | Nucleotide metabolism and signal transduction | Cell signaling | (Huynh et al., 2015) |
| 16 | WP_012462180.1 | Mn | Cytoplasm | HD domain | Nucleotide metabolism and signal transduction | Cell signaling | (Huynh et al., 2015) |
| 17 | WP_012461119.1 | Mn | Cytoplasm | HD domain | Nucleotide metabolism and signal transduction | Cell signaling | (Huynh et al., 2015) |
| 18 | WP_012461472.1 | Mn | Cytoplasm | HD domain | Nucleotide metabolism and signal transduction | Cell signaling | (Huynh et al., 2015) |
| 19 | WP_012462073.1 | Mn | Cytoplasm | HD domain | Nucleotide metabolism and signal transduction | Cell signaling | (Huynh et al., 2015) |
| 20 | WP_012462097.1 | Mn | Cytoplasm | HD domain | Nucleotide metabolism and signal transduction | Cell signaling | (Huynh et al., 2015) |
| 21 | WP_012461879.1 | Mg, Ca, Zn, Mn | Cytoplasm | DNA gyrase A | DNA metabolism, topological change and chromosome condensation | Gene expression and regulation | (Sissi and Palumbo, 2009) |
| 22 | WP_012461232.1 | Mg, Zn | Cytoplasm | Malic enzyme, NAD-binding | Oxidative decarboxylation of malate | Metabolism | (Bologna et al., 2007) |
| 23 | WP_012461251.1 | Mg, Ca, Zn | Cytoplasm | Peptidase M17, leucine aminopeptidase/peptidase B | Processing, catalysis and degradation of intracellular protein | Proteolysis | (Liew et al., 2013) |
| 24 | WP_012462083.1 | Mg, Ca, Zn, Mn | Cytoplasm | Tryptophan synthase beta subunit-like PLP-dependent enzyme | Amino acid metabolic process | Metabolism | (Ito et al., 2008) |
| 25 | WP_012461451.1 | Zn | Inner Membrane | Proton-dependent oligopeptide transporter family | Zinc transport | Transport | (Eide, 2006) |
| 26 | WP_012461603.1 | Mg, Ca | Cytoplasm | DNA recombination and repair protein RecA | DNA repair and recombination | Gene expression and regulation | (Lusetti et al., 2003) |
| 27 | WP_012461888.1 | Mg | Inner Membrane | Phosphatidate cytidylyltransferase | Synthesis of CDP-diacylglycerol from CTP and phosphatidate | Metabolism | (Sturton and Brindley, 1977) |
| 28 | WP_012461358.1 | Mg, Zn, Fe, Cd | Extracellular | Glycoprotease family | Role in cell viabilty, modification of cell wall peptidoglycan synthesis and cell division | Metabolism | (Zheng et al., 2005) |
| 29 | WP_012461161.1 | Mg, Zn | Inner Membrane | GTP-binding protein EngA | Ribosome biogenesis | Gene expression and regulation | (Lamb et al., 2007) |
| 30 | WP_011945084.1 | Mg | Cytoplasm | Nucleoside diphosphate kinase | nucleoside diphosphate phosphorylation | Metabolism | (Biondi et al., 1998) |
| 31 | WP_012461640.1 | Ni | Cytoplasm | Endoribonuclease YbeY | rRNA processing | Gene expression and regulation | (Vercruysse et al., 2014) |
| 32 | WP_012461831.1 | Mg, Ca, Zn | Cytoplasm | DNA topoisomerase, type IA | DNA metabolism, topological change and chromosome condensation | Gene expression and regulation | (Domanico and Tse-Dinh, 1991) |
| 33 | WP_041621700.1 | Ca | Cytoplasm | Succinate dehydrogenase/fumarate reductase, flavoprotein subunit | ETC: oxidation of succinate to fumarate in the cytoplasm and reduction of quinone to quinol in the membrane | Metabolism | (Cecchini et al., 2002) |
| 34 | WP_012460952.1 | Mg, Ca, Zn | Cytoplasm | Leucine-tRNA ligase | tRNA aminoacylation | Gene expression and regulation | (Tu et al., 2000) |
| 35 | WP_012460747.1 | Mg | Cytoplasm | Poly A polymerase, head domain | RNA polyadenylation | Gene expression and regulation | (Yehudai-Resheff and Schuster, 2000) |
| 36 | WP_012461403.1 | Zn, Mn, Co | Cytoplasm | Peptidase family M20/M25/M40 | Metal based metabolic process | Metabolism | (Tanimoto et al., 2008) |
| 37 | WP_012460979.1 | Mg | Cytoplasm | Peroxiredoxin, AhpC-type | Antioxidant defence | Cell signaling | (Wood et al., 2003) |
| 38 | WP_012461168.1 | Mg, Ca | Cytoplasm | Rho termination factor, RNA-binding | Transcription termination | Gene expression and regulation | (Groisman et al., 2013) |
| 39 | WP_012462318.1 | Ca, Zn | Cytoplasm | tRNA-specific adenosine deaminase | RNA editing and antimicrobial resistance | Post translational modification | (Wolf et al., 2002) |
| 40 | WP_012461219.1 | Mg, Ca | Cytoplasm | Heat shock protein 70 family | Response to heat induced stress | Protein folding | (Fang et al., 1997) |
| 41 | WP_012462286.1 | Mg, Ca | Cytoplasm | Heat shock protein 70 family | Response to heat induced stress | Protein folding | (Fang et al., 1997) |
| 42 | WP_045914353.1 | Fe | Inner Membrane | Succinate dehydrogenase/Fumarate reductase transmembrane subunit | ETC: oxidation of succinate to fumarate in the cytoplasm and reduction of quinone to quinol in the membrane | Metabolism | (Cecchini et al., 2002) |
| 43 | WP_012462281.1 | Mg | Cytoplasm | UDP-N-acetylmuramoylalanyl-D-glutamate-2,6-diaminopimelate ligase (murE) | Regulation of cell division and cell shape of bacterial cell wall | Metabolism | (Munshi et al., 2013) |
| 44 | WP_050731348.1 | Mg, Ca, Zn, Mn | Cytoplasm | DNA polymerase A | DNA replication | Gene expression and regulation | (Vashishtha et al., 2016) |
| 45 | WP_012462038.1 | Cd | Inner Membrane | Beta-lactamase/transpeptidase-like | Peptidoglycan biosynthesis for cellwall formation | Antimicrobial resistance | (Yoon et al., 1991) |
| 46 | WP_012462282.1 | Mg, Cd | Inner Membrane | Beta-lactamase/transpeptidase-like | Peptidoglycan biosynthesis for cellwall formation | Antimicrobial resistance | (Davies and Abraham, 1974) |
| 47 | WP_012461377.1 | Zn, Mn, Ni | Cytoplasm | TatD family | DNA repair | Gene expression and regulation | (Chen et al., 2014) |
| 48 | WP_012461546.1 | Mg, Mn, Co | Cytoplasm | Folylpolyglutamate synthetase | Synthesis of folic acid containing compounds | Metabolism | (Young et al., 2008) |
| 49 | WP_012461437.1 | Mg, Zn | Cytoplasm | GTP-binding protein, ribosome biogenesis, YsxC | Ribosome assembly | Gene expression and regulation | (KONG et al., 2016) |
| 50 | WP_012462280.1 | Mg | Cytoplasm | UDP-N-acetylmuramoyl-tripeptide--D-alanyl-D-alanine ligase (murF) | Final step of cellwall peptidoglycan synthesis and cellwall organization | Metabolism | (Munshi et al., 2013) |
| 51 | WP_012461990.1 | Mg, Fe | Inner Membrane | Ankyrin repeat-containing domain | Insert traffic to different subcellular localization or show a tropism for secretory pathways of host cell | Cell Signaling | (Al-Khodor et al., 2010; VieBrock et al., 2015) |
| 52 | WP_012461967.1 | Mg, Fe | Cytoplasm | Ankyrin repeat-containing domain | Insert traffic to different subcellular localization or show a tropism for secretory pathways of host cell | Cell Signaling | (Al-Khodor et al., 2010; VieBrock et al., 2015) |
| 53 | WP_012461000.1 | Mg, Fe | Cytoplasm | Ankyrin repeat-containing domain | Insert traffic to different subcellular localization or show a tropism for secretory pathways of host cell | Cell Signaling | (Al-Khodor et al., 2010; VieBrock et al., 2015) |
| 54 | WP_012461452.1 | Mg, Fe | Inner Membrane | Ankyrin repeat-containing domain | Insert traffic to different subcellular localization or show a tropism for secretory pathways of host cell | Cell Signaling | (Al-Khodor et al., 2010; VieBrock et al., 2015) |
| 55 | WP_012460963.1 | Mg, Fe | Cytoplasm | Ankyrin repeat-containing domain | Insert traffic to different subcellular localization or show a tropism for secretory pathways of host cell | Cell Signaling | (Al-Khodor et al., 2010; VieBrock et al., 2015) |
| 56 | WP_012461365.1 | Mg | Cytoplasm | Ankyrin repeat-containing domain | Insert traffic to different subcellular localization or show a tropism for secretory pathways of host cell | Cell Signaling | (Al-Khodor et al., 2010; VieBrock et al., 2015) |
| 57 | WP_012460923.1 | Mg, Fe | Cytoplasm | Ankyrin repeat-containing domain | Insert traffic to different subcellular localization or show a tropism for secretory pathways of host cell | Cell Signaling | (Al-Khodor et al., 2010; VieBrock et al., 2015) |
| 58 | WP_012460772.1 | Mg, Fe | Cytoplasm | Ankyrin repeat-containing domain | Insert traffic to different subcellular localization or show a tropism for secretory pathways of host cell | Cell Signaling | (Al-Khodor et al., 2010; VieBrock et al., 2015) |
| 59 | WP_012461252.1 | Mg | Extracellular | Ankyrin repeat-containing domain | Insert traffic to different subcellular localization or show a tropism for secretory pathways of host cell | Cell Signaling | (Al-Khodor et al., 2010; VieBrock et al., 2015) |
| 60 | WP_012461809.1 | Mg | Inner Membrane | Ankyrin repeat-containing domain | Insert traffic to different subcellular localization or show a tropism for secretory pathways of host cell | Cell Signaling | (Al-Khodor et al., 2010; VieBrock et al., 2015) |
| 61 | WP_012460919.1 | Mg, Fe | Cytoplasm | Ankyrin repeat-containing domain | Insert traffic to different subcellular localization or show a tropism for secretory pathways of host cell | Cell Signaling | (Al-Khodor et al., 2010; VieBrock et al., 2015) |
| 62 | WP_012461786.1 | Fe | Cytoplasm | Ankyrin repeat-containing domain | Insert traffic to different subcellular localization or show a tropism for secretory pathways of host cell | Cell Signaling | (Al-Khodor et al., 2010; VieBrock et al., 2015) |
| 63 | WP_012462035.1 | Mg, Fe | Cytoplasm | Ankyrin repeat-containing domain | Insert traffic to different subcellular localization or show a tropism for secretory pathways of host cell | Cell Signaling | (Al-Khodor et al., 2010; VieBrock et al., 2015) |
| 64 | WP_012461076.1 | Mg, Fe | Cytoplasm | Ankyrin repeat-containing domain | Insert traffic to different subcellular localization or show a tropism for secretory pathways of host cell | Cell Signaling | (Al-Khodor et al., 2010; VieBrock et al., 2015) |
| 65 | WP_080503901.1 | Mg | Cytoplasm | Ankyrin repeat-containing domain | Insert traffic to different subcellular localization or show a tropism for secretory pathways of host cell | Cell Signaling | (Al-Khodor et al., 2010; VieBrock et al., 2015) |
| 66 | WP_012462259.1 | Mg | Cytoplasm | Ankyrin repeat-containing domain | Insert traffic to different subcellular localization or show a tropism for secretory pathways of host cell | Cell Signaling | (Al-Khodor et al., 2010; VieBrock et al., 2015) |
| 67 | WP_012461015.1 | Mg | Cytoplasm | Ankyrin repeat-containing domain | Insert traffic to different subcellular localization or show a tropism for secretory pathways of host cell | Cell Signaling | (Al-Khodor et al., 2010; VieBrock et al., 2015) |
| 68 | WP_050731357.1 | Mg | Cytoplasm | Ankyrin repeat-containing domain | Insert traffic to different subcellular localization or show a tropism for secretory pathways of host cell | Cell Signaling | (Al-Khodor et al., 2010; VieBrock et al., 2015) |
| 69 | WP_050731368.1 | Mg | Cytoplasm | Ankyrin repeat-containing domain | Insert traffic to different subcellular localization or show a tropism for secretory pathways of host cell | Cell Signaling | (Al-Khodor et al., 2010; VieBrock et al., 2015) |
| 70 | WP_012460871.1 | Mg | Cytoplasm | Ankyrin repeat-containing domain | Insert traffic to different subcellular localization or show a tropism for secretory pathways of host cell | Cell Signaling | (Al-Khodor et al., 2010; VieBrock et al., 2015) |
| 71 | WP_012460990.1 | Mg | Cytoplasm | Ankyrin repeat-containing domain | Insert traffic to different subcellular localization or show a tropism for secretory pathways of host cell | Cell Signaling | (Al-Khodor et al., 2010; VieBrock et al., 2015) |
| 72 | WP_012461670.1 | Mg | Cytoplasm | Ankyrin repeat-containing domain | Insert traffic to different subcellular localization or show a tropism for secretory pathways of host cell | Cell Signaling | (Al-Khodor et al., 2010; VieBrock et al., 2015) |
| 73 | WP_012461946.1 | Mg | Cytoplasm | Ankyrin repeat-containing domain | Insert traffic to different subcellular localization or show a tropism for secretory pathways of host cell | Cell Signaling | (Al-Khodor et al., 2010; VieBrock et al., 2015) |
| 74 | WP_012461220.1 | Zn | Cytoplasm | DnaJ domain | Regulate the activity of HSP70 | Protein folding | (Banecki et al., 1996) |
| 75 | WP_012462285.1 | Zn | Cytoplasm | DnaJ domain | Regulate the activity of HSP70 | Protein folding | (Banecki et al., 1996) |
| 76 | WP_012460950.1 | Mg, Ca, Mn | Inner Membrane | Magnesium transporter, MgtE intracellular domain | Metal ion transport | Transport | (Chakravarty et al., 2017) |
| 77 | WP_012461389.1 | Zn | Cytoplasm | Cysteinyl-tRNA synthetase/mycothiol ligase | tRNA aminoacylation | Gene expression and regulation | (Zhang et al., 2003) |
| 78 | WP_012460797.1 | Mg, Zn | Cytoplasm | Integrase/recombinase | Site specific recombination | Gene expression and regulation | (McEwan et al., 2011) |
| 79 | WP_012462273.1 | Mg, Zn | Cytoplasm | Integrase/recombinase | Site specific recombination | Gene expression and regulation | (McEwan et al., 2011) |
| 80 | WP_012461858.1 | Mg, Zn | Cytoplasm | Isoleucine-tRNA ligase | tRNA aminoacylation | Gene expression and regulation | (CARR et al., 1975) |
| 81 | WP_012461806.1 | Cu | Inner Membrane | Copper chaperone PCu(A)C | Biogenesis and assembly of respiratory complex | Protein folding | (Thompson et al., 2012) |
| 82 | WP_012461609.1 | Mg | Cytoplasm | Ribosomal protein L2 | Translation | Gene expression and regulation | (Petrov et al., 2012) |
| 83 | WP_012461444.1 | Cu | Cytoplasm | Divalent ion tolerance protein, CutA | Response to ion tolerance | Stress response regulator | (Rensing and Grass, 2003) |
| 84 | WP_012461432.1 | Mg | Cytoplasm | DNA gyrase B | DNA metabolism, topological change and chromosome condensation | Gene expression and regulation | (Sissi and Palumbo, 2009) |
| 85 | WP_041621667.1 | Mg | Cytoplasm | Formyl transferase | Initiation of protein synthesis | Gene expression and regulation | (KAHN et al., 1980) |
| 86 | WP_012462053.1 | Mg | Cytoplasm | Deoxycytidine triphosphate deaminase/ dUTPase-like | 2'-deoxyribonucleotide metabolic process | Metabolism | (Hizi and Herzig, 2015) |
| 87 | WP_012461337.1 | Fe | Inner Membrane | Cytochrome b/b6/petB | Electron transport | Metabolism | (Trumpower, 1990) |
| 88 | WP_012461616.1 | Mn | Cytoplasm | Ribosomal protein L24 | Translation:RNA folding during early assembly | Gene expression and regulation | (Sloof et al., 1978) |
| 89 | WP_012461310.1 | Mg | Cytoplasm | Aspartate kinase domain | Biosynthesis of bacterial cellwall consituents peptidoglycan | Metabolism | (Chaitanya et al., 2010) |
| 90 | WP_012462322.1 | Mg, Ca, Zn, Fe | Cytoplasm | Endonuclease III | DNA Base-exision repair | Gene expression and regulation | (Kow, 1989) |
| 91 | WP_041621687.1 | Mg | Cytoplasm | Tetrahydrodipicolinate N-succinyltransferase, transferase hexapeptide repeat family/DapD | Lysine biosynthesis via diaminopimelate | Metabolism | (Schuldt et al., 2009) |
| 92 | WP_012460810.1 | Mg | Cytoplasm | SAICAR synthetase | Purine biosynthesis pathway | Metabolism | (Wolf et al., 2014) |
| 93 | WP_012461883.1 | Fe | Cytoplasm | Cytochrome C1 family | Electron transport | Metabolism | (Trumpower, 1990) |
| 94 | WP_012461155.1 | Mg | Cytoplasm | UvrD-like DNA helicase | DNA repair, replication and recombination | Gene expression and regulation | (Curti et al., 2007) |
| 95 | WP_012461312.1 | Ca, Zn | Inner Membrane | Cation/H+ exchanger | Transfer of cations across the membrane | Transport | (Marquis et al., 1976) |
| 96 | WP_012461270.1 | Mg | Inner Membrane | Transporter associated domain/CorC_HlyC | Transport of ions | Transport | (Gibson et al., 1991) |
| 97 | WP_012461641.1 | Mg | Inner Membrane | Transporter associated domain/CorC_HlyC | Transport of ions | Transport | (Gibson et al., 1991) |
| 98 | WP_012461818.1 | Fe | Periplasm | Cytochrome c | Electron transport | Metabolism | (Trumpower, 1990) |
| 99 | WP_012460983.1 | Zn | Cytoplasm | Zinc finger, CHC2-type, DNA primase, DnaG | DNA replication: RNA primer synthesis | Gene expression and regulation | (Kuron et al., 2014) |
| 100 | WP_012461022.1 | Ca, Cd | Inner Membrane | Tetratricopeptide repeat | Involved in different cellular processes by protein-protein interactioion module | Gene expression and regulation | (Bang et al., 2016; Cerveny et al., 2013) |
| 101 | WP_012462074.1 | Ca, Cd, Ni | Outer Membrane | Tetratricopeptide repeat | Involved in different cellular processes by protein-protein interactioion module | Gene expression and regulation | (Bang et al., 2016; Cerveny et al., 2013) |
| 102 | WP_012462109.1 | Mg, Ca, Cd, Ni | Inner Membrane | Tetratricopeptide repeat | Involved in different cellular processes by protein-protein interactioion module | Gene expression and regulation | (Bang et al., 2016; Cerveny et al., 2013) |
| 103 | WP_012460843.1 | Ca, Cd | Inner Membrane | Tetratricopeptide repeat | Involved in different cellular processes by protein-protein interactioion module | Gene expression and regulation | (Bang et al., 2016; Cerveny et al., 2013) |
| 104 | WP_080503942.1 | Ca | Outer Membrane | Tetratricopeptide repeat | Involved in different cellular processes by protein-protein interactioion module | Gene expression and regulation | (Bang et al., 2016; Cerveny et al., 2013) |
| 105 | WP_012461290.1 | Cd | Inner Membrane | Tetratricopeptide repeat | Involved in different cellular processes by protein-protein interactioion module | Gene expression and regulation | (Bang et al., 2016; Cerveny et al., 2013) |
| 106 | WP_012461904.1 | Ca | Inner Membrane | Tetratricopeptide repeat | Involved in different cellular processes by protein-protein interactioion module | Gene expression and regulation | (Bang et al., 2016; Cerveny et al., 2013) |
| 107 | WP_012460938.1 | Mg, Ca, Cd | Outer Membrane | Tetratricopeptide repeat | Involved in different cellular processes by protein-protein interactioion module | Gene expression and regulation | (Bang et al., 2016; Cerveny et al., 2013) |
| 108 | WP_012461681.1 | Mg, Ca, Cd | Inner Membrane | Tetratricopeptide repeat | Involved in different cellular processes by protein-protein interactioion module | Gene expression and regulation | (Bang et al., 2016; Cerveny et al., 2013) |
| 109 | WP_012461080.1 | Mg, Cd, Ni | Inner Membrane | Tetratricopeptide repeat | Involved in different cellular processes by protein-protein interactioion module | Gene expression and regulation | (Bang et al., 2016; Cerveny et al., 2013) |
| 110 | WP_012462244.1 | Mg, Ca, Cd | Inner Membrane | Tetratricopeptide repeat | Involved in different cellular processes by protein-protein interactioion module | Gene expression and regulation | (Bang et al., 2016; Cerveny et al., 2013) |
| 111 | WP_012462274.1 | Mg, Zn, Fe | Cytoplasm | tRNA modification GTPase MnmE | tRNA modification | Post translational modification | (Prado et al., 2013) |
| 112 | WP_012462050.1 | Ca, Mn, Fe | Cytoplasm | NADH:ubiquinone oxidoreductase, subunit G | folic acid-containing compound biosynthetic process | Metabolism | (Brandt et al., 2003) |
| 113 | WP_041621579.1 | Mg | Inner Membrane | Type-IV secretion system protein TraC/Conjugative transfer ATPase | Assembly of F conjugative pilus | Transport | (Schandel et al., 1992) |
| 114 | WP_012461164.1 | Mg, Ca | Cytoplasm | Thymidylate kinase | DNA replication: Phosphorylation of dTMP to form dTDP | Gene expression and regulation | (Fioravanti et al., 2005) |
| 115 | WP_012460972.1 | Mg | Cytoplasm | GTP-binding protein Obg/CgtA | Chromosome partition, DNA replication, stress response | Gene expression and regulation | (Buglino et al., 2002) |
| 116 | WP_012461635.1 | Mg | Outer Membrane | Competence-damaged protein, CinA | Genetic recombination | Gene expression and regulation | (Zhu et al., 2015) |
| 117 | WP_012461260.1 | Mg, Zn, Co | Cytoplasm | Translation initiation factor aIF-2, bacterial-like | Translational initiation | Gene expression and regulation | (Nikonov et al., 2007) |
| 118 | WP_012460994.1 | Mg, Zn | Cytoplasm | Ribonuclease E/G family | mRNA degradation | Gene expression and regulation | (Callaghan et al., 2005; Lawal et al., 2011) |
| 119 | WP_012461727.1 | Mg, Ca, Mn | Cytoplasm | AP endonuclease 1 | DNA Base-exision repair | Gene expression and regulation | (Tsutakawa et al., 2013) |
| 120 | WP_012460897.1 | Mg, Ca, Mn | Cytoplasm | AP endonuclease 1 | DNA Base-exision repair | Gene expression and regulation | (Tsutakawa et al., 2013) |
| 121 | WP_012461746.1 | Mg, Ca, Mn | Cytoplasm | AP endonuclease 1 | DNA Base-exision repair | Gene expression and regulation | (Tsutakawa et al., 2013) |
| 122 | WP_012461889.1 | Mg, Fe | Cytoplasm | Decaprenyl diphosphate synthase-like | Biosynthesis of cellwall | Metabolism | (Chan et al., 2014) |
| 123 | WP_012461354.1 | Mg, Zn | Cytoplasm | Delta-aminolevulinic acid dehydratase | Biosynthesis of porphobilinogen | Metabolism | (Spencer and Jordan, 1993) |
| 124 | WP_012461846.1 | Zn | Cytoplasm | Lysine-tRNA ligase | Lysyl-tRNA aminoacylation | Gene expression and regulation | (Chen et al., 2013) |
| 125 | WP_012461981.1 | Zn | Cytoplasm | N-acetylmuramoyl-L-alanine amidase domain | Peptidoglycal catabolic process | Metabolism | (Kerff et al., 2010; Yang et al., 2013) |
| 126 | WP_012460745.1 | Mg | Cytoplasm | UvrD-like DNA helicase | DNA repair, replication and recombination | Gene expression and regulation | (Curti et al., 2007) |
| 127 | WP_012462208.1 | Mg, Mn | Cytoplasm | Aspartyl/Asparaginyl-tRNA synthetase, class IIb | tRNA aminoacylation | Gene expression and regulation | (Thompson and Simonson, 2006) |
| 128 | WP_012460949.1 | Mg, Zn | Cytoplasm | Peptidase M32, carboxypeptidase Taq | Proteolysis | Proteolysis | (Lee et al., 1994) |
| 129 | WP_012461393.1 | Ca | Inner Membrane | Quinoprotein alcohol dehydrogenase-like domain | Oxidation of alcohol | Metabolism | (Rozeboom et al., 2015) |
| 130 | WP_012461223.1 | Ca, Fe | Cytoplasm | Cysteine desulfurase | Biosynthesis of Fe-S cluster | Metabolism | (Mihara and Esaki, 2002) |
| 131 | WP_012461224.1 | Ca, Fe | Cytoplasm | Cysteine desulfurase | Biosynthesis of Fe-S cluster | Metabolism | (Mihara and Esaki, 2002) |
| 132 | WP_012460803.1 | Mg, Ca | Cytoplasm | DNA helicase, RecD-like | Separation of DNA duplexes into single strands in an ATP-dependent manner | Gene expression and regulation | (Wang and Julin, 2004) |
| 133 | WP_012461832.1 | Ca, Cd | Cytoplasm | L-lactate/malate dehydrogenase | Carbohydrate metabolic process | Metabolism | (Gordon and Doelle, 1976) |
| 134 | WP_012461360.1 | Ca, Mn | Cytoplasm | DNA polymerase 3, epsilon subunit | DNA replication, nucleotide and base excision repair | Gene expression and regulation | (Cisneros et al., 2009; Robinson et al., 2012) |
| 135 | WP_012461055.1 | Mg | Cytoplasm | OmpR/PhoB-type DNA-binding domain | Stress sensor response regulator | Cell signaling | (Sola et al., 2006) |
| 136 | WP_012461406.1 | Mg, Ca | Cytoplasm | OmpR/PhoB-type DNA-binding domain | Stress sensor response regulator | Cell signaling | (Sola et al., 2006) |
| 137 | WP_012460965.1 | Mg | Cytoplasm | Chaperonin Cpn60 | Stabilise and protect disassembled polypeptides in response to heat-shock conditions | Protein folding | (Schmidt et al., 1992) |
| 138 | WP_012461388.1 | Zn | Cytoplasm | Glutamyl/glutaminyl-tRNA synthetase | tRNA aminoacylation | Gene expression and regulation | (Banerjee et al., 2004) |
| 139 | WP_041621635.1 | Mg, Ca, Zn | Cytoplasm | Glutamyl/glutaminyl-tRNA synthetase | tRNA aminoacylation | Gene expression and regulation | (Banerjee et al., 2004) |
| 140 | WP_012462272.1 | Zn | Cytoplasm | Zinc finger, DksA/TraR C4-type | Regulation of rRNA promoters and virulence factor formation | Gene expression and regulation | (Jude et al., 2003) |
| 141 | WP_012461431.1 | Mg | Cytoplasm | HAD-hyrolase-like | Detoxification of metabolic by-products | Metabolism | (Kuznetsova et al., 2015) |
| 142 | WP_012461501.1 | Mn, Fe | Cytoplasm | Respiratory chain NADH ubiquinone oxidoreductase, F subunit | Electron transport | Metabolism | (Kaur et al., 2017) |
| 143 | WP_012461493.1 | Mg, Ca | Cytoplasm | UDP-N-acetylglucosamine 1-carboxyvinyltransferase (murA) | UDP-N-acetylgalactosamine biosynthetic process | Metabolism | (Munshi et al., 2013) |
| 144 | WP_012461052.1 | Cd | Cytoplasm | Glutamyl-tRNA(Gln) amidotransferase, subunit B, conserved site | Formation of Gln-tRNA (Gln) by tramsamidation of misacylated Glu-tRNA (Gln) | Gene expression and regulation | (Kwak et al., 2002) |
| 145 | WP_012461051.1 | Ca, Zn, Mn | Cytoplasm | Glutamyl-tRNA(Gln) amidotransferase, subunit B, conserved site | tRNA recongnition | Gene expression and regulation | (Nakamura et al., 2009) |
| 146 | WP_012460962.1 | Mg, Ca | Cytoplasm | DnaB-like helicase | DNA replication: Separation of DNA duplexes into single strands in an ATP-dependent manner | Gene expression and regulation | (Soni et al., 2003) |
| 147 | WP_012460873.1 | Mg, Ca | Cytoplasm | DnaB-like helicase | DNA replication: Separation of DNA duplexes into single strands in an ATP-dependent manner | Gene expression and regulation | (Soni et al., 2003) |
| 148 | WP_012460958.1 | Mg, Ca | Cytoplasm | DnaB-like helicase | DNA replication: Separation of DNA duplexes into single strands in an ATP-dependent manner | Gene expression and regulation | (Soni et al., 2003) |
| 149 | WP_012460901.1 | Mg, Ca | Cytoplasm | DnaB-like helicase | DNA replication: Separation of DNA duplexes into single strands in an ATP-dependent manner | Gene expression and regulation | (Soni et al., 2003) |
| 150 | WP_012460884.1 | Mg, Ca | Cytoplasm | DnaB-like helicase | DNA replication: Separation of DNA duplexes into single strands in an ATP-dependent manner | Gene expression and regulation | (Soni et al., 2003) |
| 151 | WP_012461125.1 | Mg, Ca | Cytoplasm | DnaB-like helicase | DNA replication: Separation of DNA duplexes into single strands in an ATP-dependent manner | Gene expression and regulation | (Soni et al., 2003) |
| 152 | WP_012462153.1 | Mg, Ca | Cytoplasm | DnaB-like helicase | DNA replication: Separation of DNA duplexes into single strands in an ATP-dependent manner | Gene expression and regulation | (Soni et al., 2003) |
| 153 | WP_012462134.1 | Mg, Ca | Cytoplasm | DnaB-like helicase | DNA replication: Separation of DNA duplexes into single strands in an ATP-dependent manner | Gene expression and regulation | (Soni et al., 2003) |
| 154 | WP_012461013.1 | Mg, Ca | Cytoplasm | DnaB-like helicase | DNA replication: Separation of DNA duplexes into single strands in an ATP-dependent manner | Gene expression and regulation | (Soni et al., 2003) |
| 155 | WP_012462261.1 | Mg, Ca | Cytoplasm | DnaB-like helicase | DNA replication: Separation of DNA duplexes into single strands in an ATP-dependent manner | Gene expression and regulation | (Soni et al., 2003) |
| 156 | WP_012460992.1 | Mg, Ca | Cytoplasm | DnaB-like helicase | DNA replication: Separation of DNA duplexes into single strands in an ATP-dependent manner | Gene expression and regulation | (Soni et al., 2003) |
| 157 | WP_012462325.1 | Mg, Ca, Cu | Cytoplasm | DnaB-like helicase | DNA replication: Separation of DNA duplexes into single strands in an ATP-dependent manner | Gene expression and regulation | (Soni et al., 2003) |
| 158 | WP_012461596.1 | Mg, Ca | Cytoplasm | DnaB-like helicase | DNA replication: Separation of DNA duplexes into single strands in an ATP-dependent manner | Gene expression and regulation | (Soni et al., 2003) |
| 159 | WP_012461957.1 | Mg, Ca | Cytoplasm | DnaB-like helicase | DNA replication: Separation of DNA duplexes into single strands in an ATP-dependent manner | Gene expression and regulation | (Soni et al., 2003) |
| 160 | WP_012461476.1 | Mg, Ca | Cytoplasm | DnaB-like helicase | DNA replication: Separation of DNA duplexes into single strands in an ATP-dependent manner | Gene expression and regulation | (Soni et al., 2003) |
| 161 | WP_012460959.1 | Mg, Ca | Cytoplasm | DnaB-like helicase | DNA replication: Separation of DNA duplexes into single strands in an ATP-dependent manner | Gene expression and regulation | (Soni et al., 2003) |
| 162 | WP_012461008.1 | Ca | Cytoplasm | DnaB-like helicase | DNA replication: Separation of DNA duplexes into single strands in an ATP-dependent manner | Gene expression and regulation | (Soni et al., 2003) |
| 163 | WP_012460969.1 | Ca | Inner Membrane | Inhibitor of apoptosis-promoting Bax1 | Calcium homeostasis | Cell signaling | (Chang et al., 2014) |
| 164 | WP_012461235.1 | Zn | Cytoplasm | Citrate synthase | Energy generation and carbon assimilation | Metabolism | (Suksomtip et al., 2005) |
| 165 | WP_012461239.1 | Mg | Cytoplasm | ATPase, F1/V1 complex, beta subunit | ATP synthesis coupled proton transport | Transport | (Maher et al., 2009) |
| 166 | WP_012461551.1 | Mg | Cytoplasm | ATPase, F1/V1 complex, beta subunit | ATP synthesis coupled proton transport | Transport | (Maher et al., 2009) |
| 167 | WP_012461552.1 | Mg | Cytoplasm | ATP synthase, F1 complex | ATP synthesis coupled proton transport | Transport | (Schobert, 1998) |
| 168 | WP_041621568.1 | Fe | Cytoplasm | 4Fe-4S ferredoxin-type, iron-sulphur binding domain | Electron transport | Metabolism | (Brzóska et al., 2006) |
| 169 | WP_041621803.1 | Mg, Mn, Ni | Cytoplasm | UDP-N-acetylmuramate--L-alanine ligase (murC) | Biogenesis of cellwall peptidoglycan | Metabolism | (Moraes et al., 2015; Munshi et al., 2013) |
| 170 | WP_012461601.1 | Zn | Cytoplasm | Phosphopantetheine binding ACP domain | Fatty acid biosynthesis | Metabolism | (Roujeinikova et al., 2002) |
| 171 | WP_012462279.1 | Mg | Inner Membrane | Phospho-N-acetylmuramoyl-pentapeptide transferase | Assembly of peptidoglycan layer of bacterial cellwall | Metabolism | (Lloyd et al., 2004) |
| 172 | WP_012460966.1 | Mg, Ca, Ni | Cytoplasm | Phosphopantetheine-protein transferase domain | Biosynthesis of fatty acid, polyketides and antibiotic | Metabolism | (Finking et al., 2002) |
| 173 | WP_012461924.1 | Mn | Cytoplasm | DHHA1 domain | c-di-AMP degradation and regulate di-c-GMP signaling pathway | Cell signaling | (He et al., 2016) |
| 174 | WP_012462162.1 | Mg, Zn | Cytoplasm | DNA polymerase III, subunit gamma/ tau | DNA replication | Gene expression and regulation | (Robinson et al., 2012; Walker et al., 2000) |
| 175 | WP_012462078.1 | Mg, Ca, Mn, Co | Cytoplasm | Ribonuclease H domain | Degradation of RNA in RNA.DNA hybrids | Gene expression and regulation | (Goedken and Marqusee, 1999) |
| 176 | WP_012461533.1 | Mg, Ca | Cytoplasm | D-alanine--D-alanine ligase, C-terminal | Biosynthesis of cellwall peptidoglycan | Metabolism | (Tytgat et al., 2009) |
| 177 | WP_012460742.1 | Zn | Periplasm | Bacterial solute-binding proteins, family 3 | Transport of solute ions and virulence | Transport | (Neupane et al., 2017) |
| 178 | WP_012461229.1 | Mg | Inner Membrane | Glycosyl transferase family group 2 | Biosynthesis of carbohydrates | Metabolism | (El Qaidi et al., 2018; Schmid et al., 2016) |
| 179 | WP_012461928.1 | Zn | Cytoplasm | RNA-binding S4 domain | Translation: Small subunit ribosomal assembly | Gene expression and regulation | (Chen et al., 2009) |
| 180 | WP_012460731.1 | Mg | Cytoplasm | AAA+ ATPase domain | To form ring shaped oligomers crucial for ATPase activities | Proteolysis | (Ogura and Wilkinson, 2001) |
| 181 | WP_011945048.1 | Ca | Cytoplasm | 'Cold-shock' DNA-binding domain | Response to temperature downshift | Stress response regulator | (Keto-Timonen et al., 2016) |
| 182 | WP_012460998.1 | Ca, Zn | Cytoplasm | Cell shape determining protein MreB | Cell shape maintenance and cell morphogenesis | Gene expression and regulation | (Soufo et al., 2010) |
| 183 | WP_012461445.1 | Ca, Mn, Cd | Inner Membrane | Signal recognition particle, SRP54 subunit, GTPase domain | SRP-dependent protein targeting to membrane | Cell signaling | (Focia et al., 2004) |
| 184 | WP_041621643.1 | Ca, Cd | Inner Membrane | Signal recognition particle, SRP54 subunit, GTPase domain | SRP-dependent protein targeting to membrane | Cell signaling | (Focia et al., 2004) |
| 185 | WP_012461499.1 | Mn, Fe | Cytoplasm | Respiratory chain NADH dehydrogenase 49 Kd subunit signature. | Electron transport | Metabolism | (Kaur et al., 2017) |
| 186 | WP_012462223.1 | Mg, Ca, Mn | Inner Membrane | EAL domain | Hydrolysis of c-di-GMP | Cell signaling | (Ryan et al., 2006) |
| 187 | WP_041621787.1 | Fe | Inner Membrane | Ubiquitinol-cytochrome C reductase, Fe-S subunit, TAT signal | ETC: oxidation of ubiquinol and cytochrome c | Metabolism | (Trumpower, 1990) |
| 188 | WP_012461772.1 | Fe | Cytoplasm | 7Fe ferredoxin | Electron transport | Metabolism | (Ricagno et al., 2007; Ugalde et al., 2019) |
| 189 | WP_012461555.1 | Mg | Cytoplasm | Alanine-tRNA ligase, class IIc | tRNA aminoacylation | Gene expression and regulation | (DIGNAM et al., 1991) |
| 190 | WP_012461508.1 | Mg | Cytoplasm | Crossover junction endodeoxyribonuclease RuvC | DNA recombination and repair | Gene expression and regulation | (Bennett and West, 1995) |
| 191 | WP_012460964.1 | Ca | Cytoplasm | Chaperonin 10 | Heat stress response and protien folding | Protein folding | (Roberts et al., 2003) |
| 192 | WP_012462160.1 | Mg, Fe | Inner Membrane | ABC transporter | Import and export of nutrients, toxic substances and metal ions | Transport | (Garmory and Titball, 2004; Ma et al., 2009) |
| 193 | WP_012461925.1 | Mg | Inner Membrane | ABC transporter | Import and export of nutrients, toxic substances and metal ions | Transport | (Garmory and Titball, 2004; Ma et al., 2009) |
| 194 | WP_012462224.1 | Mg | Inner Membrane | ABC transporter | Import and export of nutrients, toxic substances and metal ions | Transport | (Garmory and Titball, 2004; Ma et al., 2009) |
| 195 | WP_012461341.1 | Mg, Ni | Inner Membrane | ABC transporter | Import and export of nutrients, toxic substances and metal ions | Transport | (Garmory and Titball, 2004; Ma et al., 2009) |
| 196 | WP_012461666.1 | Mg, Ni | Inner Membrane | ABC transporter | Import and export of nutrients, toxic substances and metal ions | Transport | (Garmory and Titball, 2004; Ma et al., 2009) |
| 197 | WP_012462340.1 | Mg | Inner Membrane | ABC transporter | Import and export of nutrients, toxic substances and metal ions | Transport | (Garmory and Titball, 2004; Ma et al., 2009) |
| 198 | WP_012461405.1 | Mg | Inner Membrane | ABC transporter | Import and export of nutrients, toxic substances and metal ions | Transport | (Garmory and Titball, 2004; Ma et al., 2009) |
| 199 | WP_012461953.1 | Mg | Inner Membrane | ABC transporter | Import and export of nutrients, toxic substances and metal ions | Transport | (Garmory and Titball, 2004; Ma et al., 2009) |
| 200 | WP_012461184.1 | Mg, Ni | Inner Membrane | ABC transporter | Import and export of nutrients, toxic substances and metal ions | Transport | (Garmory and Titball, 2004; Ma et al., 2009) |
| 201 | WP_012462277.1 | Mg, Ni | Inner Membrane | ABC transporter | Import and export of nutrients, toxic substances and metal ions | Transport | (Garmory and Titball, 2004; Ma et al., 2009) |
| 202 | WP_012461063.1 | Mg, Fe | Cytoplasm | ABC transporter | Import and export of nutrients, toxic substances and metal ions | Transport | (Garmory and Titball, 2004; Ma et al., 2009) |
| 203 | WP_012461890.1 | Mg, Ca, Zn, Mn | Inner Membrane | ABC transporter | Import and export of nutrients, toxic substances and metal ions | Transport | (Garmory and Titball, 2004; Ma et al., 2009) |
| 204 | WP_012461905.1 | Mg, Zn, Fe | Cytoplasm | UvrABC system subunit A | DNA repair | Gene expression and regulation | (Van Houten, 1990) |
| 205 | WP_012461774.1 | Zn | Outer Membrane | Cytotoxic necrotizing factor-like, catalytic | Alteration in host cell actin cytoskeleton and promotes bacterial invasion | Metabolism | (El-Housseiny et al., 2010) |
| 206 | WP_012461626.1 | Mg, Ca, Zn, Co | Cytoplasm | Adenylate kinase | Phosphoryl transfer reaction | Metabolism | (Lacher and Schafer, 1993) |
| 207 | WP_012461104.1 | Mg | Cytoplasm | DNA mismatch repair protein MutS | DNA mismatch repair | Gene expression and regulation | (Lebbink et al., 2010) |
| 208 | WP_012461313.1 | Mg | Inner Membrane | 5-formyltetrahydrofolate cyclo-ligase | Thiamine metabolism | Metabolism | (Meier et al., 2007) |
| 209 | WP_012461218.1 | Fe | Cytoplasm | 2Fe-2S iron-sulfur cluster binding domain | Electron transport | Metabolism | (Brzóska et al., 2006) |
| 210 | WP_012461500.1 | Fe | Inner Membrane | Thioredoxin-like [2Fe-2S] ferredoxin | Electron transport | Metabolism | (Yeh et al., 2000) |
| 211 | WP_012462269.1 | Ca | Cytoplasm | Pseudouridine synthase, RsuA/RluB/C/D/E/F | RNA modification | Post translational modification | (Mizutani et al., 2004) |
| 212 | WP_012461247.1 | Mg, Mn | Cytoplasm | Phenylalanine-tRNA ligase, class IIc, beta subunit | Phenyalanyl-tRNA aminoacylation | Gene expression and regulation | (Beyer et al., 2004) |
| 213 | WP_011944233.1 | Mg | Cytoplasm | Type IV secretion system protein VirB11 | Protein transport | Transport | (Waksman and Orlova, 2014) |
| 214 | WP_012461165.1 | Mg, Ca | Cytoplasm | GidA associated domain 3 | tRNA modification | Post translational modification | (Meyer et al., 2008) |
| 215 | WP_012461775.1 | Mg, Mn | Cytoplasm | Glutamine synthetase, catalytic domain | Nitrogen compound metabolic process | Metabolism | (Mowbray et al., 2014) |
| 216 | WP_012460728.1 | Mg, Ca | Cytoplasm | Phosphoglycerate kinase | Glycolytic process | Metabolism | (Reddy and Wendisch, 2014) |
| 217 | WP_012461498.1 | Mn | Cytoplasm | Respiratory chain NADH dehydrogenase 30 Kd subunit signature. | Electron transport | Metabolism | (Kaur et al., 2017) |
| 218 | WP_012461525.1 | Ca, Cu | Cytoplasm | Copper chaperone SCO1/SenC, Thioredoxin domain | Biogenesis and assembly of respiratory complex | Protein folding | (Trasnea et al., 2016) |
| 219 | WP_050731388.1 | Mn, Fe, Co | Cytoplasm | Ribonucleotide reductase small subunit | Provide precursor for DNA replication | Gene expression and regulation | (Griepenburg et al., 1998) |
| 220 | WP_012461836.1 | Zn, Fe, Ni, Co | Cytoplasm | Peptide deformylase | Formyl group removal of nascent polypeptide chains at N-terminal in bacteria | Gene expression and regulation | (Becker et al., 1998) |
| 221 | WP_012461185.1 | Mg | Cytoplasm | Short-chain dehydrogenase/reductase SDR | NAD and NADH oxidoreductase activity | Metabolism | (Niefind et al., 2003) |
| 222 | WP_012461602.1 | Mg, Ca | Cytoplasm | Short-chain dehydrogenase/reductase SDR | NAD and NADH oxidoreductase activity | Metabolism | (Niefind et al., 2003) |
| 223 | WP_012461632.1 | Mg | Cytoplasm | Tetrapyrrole biosynthesis, 5-aminolevulinic acid synthase | Tetrapyyrole biosynthesis | Metabolism | (Choi et al., 2004) |
| 224 | WP_012461248.1 | Ca | Cytoplasm | Translation elongation factor EFTs/EF1B, dimerisation | Translational elongation | Gene expression and regulation | (Wittinghofer et al., 1983) |
| 225 | WP_041621607.1 | Zn | Inner Membrane | Phospholipase D/Transphosphatidylase | Secondary messenger formation, host invasion and colonization | Cell signaling | (Selvy et al., 2011) |
| 226 | WP_012461844.1 | Ca | Cytoplasm | Triosephosphate isomerase | Glycolytic process | Metabolism | (Mathur et al., 2006) |
| 227 | WP_012461273.1 | Mg, Ca | Cytoplasm | 2-oxoglutarate dehydrogenase E1 component | Role in TCA cycle | Metabolism | (Rodríguez-Zavala et al., 2000) |
| 228 | WP_041621781.1 | Zn | Cytoplasm | Ribosomal protein S2 | Translation intiation complex formation | Gene expression and regulation | (Hensley et al., 2011) |
| 229 | WP_012462089.1 | Mg, Zn, Mn | Cytoplasm | Isopropylmalate dehydrogenase-like domain | Leucine biosynthesis pathway | Metabolism | (Palló et al., 2014) |
| 230 | WP_012461655.1 | Mg, Ca | Cytoplasm | Polyprenyl synthetase | Isoprenoid biosynthesis | Metabolism | (Wallrapp et al., 2013) |
| 231 | WP_012462008.1 | Mg, Ca, Fe, Cd | Cytoplasm | Thioredoxin reductase | Response to superoxide radical | Stress response regulator | (Lu et al., 2013; Rollin‐Genetet et al., 2004) |
| 232 | WP_012460984.1 | Mg | Cytoplasm | RNA polymerase sigma-70 | Regulate expression of prokaryotic transcription genes | Gene expression and regulation | (Kazmierczak et al., 2005) |
| 233 | WP_012461805.1 | Mg | Cytoplasm | RNA polymerase sigma-70 | Regulate expression of prokaryotic transcription genes | Gene expression and regulation | (Kazmierczak et al., 2005) |
| 234 | WP_012461098.1 | Co | Cytoplasm | ParB-like nuclease domain | Chromosome partition | Gene expression and regulation | (Nisa et al., 2010) |
| 235 | WP_012461512.1 | Ca | Cytoplasm | DNA methylase, N-6 adenine-specific, conserved site | DNA methylation | Gene expression and regulation | (Julio et al., 2001) |
| 236 | WP_012461600.1 | Mg, Ca, Ni | Cytoplasm | Beta-ketoacyl synthase | Bacterial fatty acid synthesis | Metabolism | (Lai and Cronan, 2003) |
| 237 | WP_012461243.1 | Ca, Zn | Cytoplasm | Acyl transferase domain | Biosynthesis of fatty acid, polyketides and toxins | Metabolism | (Kutchma et al., 1999) |
| 238 | WP_012461722.1 | Mg, Cu | Inner Membrane | Cytochrome C and Quinol oxidase polypeptide I | Coupling between dioxygen reduction and proton pumping | Metabolism | (Belevich et al., 2006) |
| 239 | WP_012461272.1 | Mg, Mn, Fe, Co | Cytoplasm | Iron/manganese superoxide dismutases, C-terminal domain | Response to oxidative stress | Stress response regulator | (Aguirre and Culotta, 2012) |
| 240 | WP_012462319.1 | Zn | Inner Membrane | Cation efflux protein | Zinc efflux | Transport | (Villa and Vinas, 2016) |
| 241 | WP_012462214.1 | Cd | Cytoplasm | Ribosomal protein L11/L12 | Translation and stringent response to harsh condition | Gene expression and regulation | (Jenvert and Schiavone, 2007) |
| 242 | WP_012462166.1 | Zn, Mn | Cytoplasm | Metallo-beta-lactamase | Catalyse the hydrolysis of almost all beta-lactam antibacterials | Antimicrobial resistance | (Aoki et al., 2010; Palzkill, 2013) |
| 243 | WP_050731342.1 | Ca, Zn, Mn | Cytoplasm | Metallo-beta-lactamase | Catalyse the hydrolysis of almost all beta-lactam antibacterials | Antimicrobial resistance | (Aoki et al., 2010; Palzkill, 2013) |
| 244 | WP_012461304.1 | Mg, Mn, Ni | Cytoplasm | UDP-N-acetylmuramoylalanine--D-glutamate ligase [murD]. | Regulation of cell division and cell shape of bacterial cell wall | Metabolism | (Jukič et al., 2019; Moraes et al., 2015; Munshi et al., 2013) |
| 245 | WP_012461828.1 | Ca | Cytoplasm | Pyridine nucleotide-disulphide oxidoreductase, class-II | Oxidative metabolic process | Metabolism | (Holmgren, 1980) |
| 246 | WP_012461557.1 | Fe | Cytoplasm | Iron-sulphur cluster biosynthesis | Iron-sulphur cluster biosynthesis | Gene expression and regulation | (Roche et al., 2013) |
| 247 | WP_012461355.1 | Ca | Inner Membrane | MotA/TolQ/ExbB proton channel family | Ion potential-driven molecular motors | Transport | (Cascales et al., 2001) |
| 248 | WP_012462316.1 | Mg, Ca, Zn, Mn, Co | Cytoplasm | Metallopeptidase family M24 | Removal of starting methionine of many proteins and act co-translationally in association with the ribosomes | Gene expression and regulation | (Rawlings and Barrett, 1995) |
| 249 | WP_041621747.1 | Mn, Co | Cytoplasm | Metallopeptidase family M24 | Removal of starting methionine of many proteins and act co-translationally in association with the ribosomes | Gene expression and regulation | (Rawlings and Barrett, 1995) |
| 250 | WP_011944977.1 | Fe | Inner Membrane | NADH ubiquinone oxidoreductase, 20 Kd subunit | Electron transport | Metabolism | (Friedrich and Scheide, 2000) |
| 251 | WP_012462043.1 | Mg, Zn | Inner Membrane | Peptidase, FtsH | ATP-dependent protease degrades misfolded membrane proteins and processing of pre-proteins | Proteolysis | (Langklotz et al., 2012) |
| 252 | WP_012460754.1 | Mg | Outer Membrane | Outer membrane protein assembly factor BamA, Surface antigen | Outer membrane protein biogenesis and assembly | Gene expression and regulation | (Albrecht et al., 2014) |
| 253 | WP_012461866.1 | Mg | Cytoplasm | Polyribonucleotide nucleotidyltransferase | RNA degradation | Gene expression and regulation | (Nurmohamed et al., 2009) |
| 254 | WP_012461884.1 | Cd | Cytoplasm | Ribonuclease PH, bacterial-type | tRNA processing | Gene expression and regulation | (Harlow et al., 2004) |
| 255 | WP_012461854.1 | Mg, Zn, Mn | Cytoplasm | Succinyl-CoA ligase, alpha subunit | Substrate level phosphorylation | Metabolism | (GRUNAU et al.) |
| 256 | WP_012461402.1 | Mg, Cd | Cytoplasm | Heat shock protein Hsp90, N-terminal | Response to heat induced stress | Protein folding | (Choi et al., 2008) |
| 257 | WP_012461349.1 | Mg, Ca, Co | Cytoplasm | Elongation factor Tu GTP binding domain | Translational elongation | Gene expression and regulation | (JENSEN et al., 1989) |
| 258 | WP_012461713.1 | Mg, Ca | Cytoplasm | Elongation factor Tu GTP binding domain | Translational elongation | Gene expression and regulation | (JENSEN et al., 1989) |
| 259 | WP_012461605.1 | Mg, Ca | Cytoplasm | Elongation factor Tu GTP binding domain | Translational elongation | Gene expression and regulation | (JENSEN et al., 1989) |
| 260 | WP_012462217.1 | Mg, Ca | Cytoplasm | Elongation factor Tu GTP binding domain | Translational elongation | Gene expression and regulation | (JENSEN et al., 1989) |
| 261 | WP_012461822.1 | Zn | Inner Membrane | Mrp/NBP35 ATP-binding protein/P-loop_NTPase | Assembly of iron sulphur cluster | Gene expression and regulation | (Camire et al., 2015) |
| 262 | WP_012461379.1 | Zn | Cytoplasm | Prisomal protein N/DEAD/DEAH box helicase domain | Resumption of DNA replication | Gene expression and regulation | (Windgassen et al., 2017) |
| 263 | WP_012460805.1 | Mg, Ca, Mn | Cytoplasm | Dihydrodipicolinate synthetase, DapA | Lysine biosynthesis via diaminopimelate | Metabolism | (Hoganson and Stahly, 1975; Pearce et al., 2017) |
| 264 | WP_012462327.1 | Mg, Ca, Zn, Mn, Cd | Cytoplasm | Ribonuclease III domain | RNA processing | Gene expression and regulation | (Sun et al., 2005) |
| 265 | WP_012461949.1 | Mg, Co | Cytoplasm | Ribonucleotide reductase large subunit, N-terminal | Provide precursor for DNA replication | Gene expression and regulation | (Jonna et al., 2015) |
| 266 | WP_011944565.1 | Mg | Cytoplasm | RNA pyrophosphohydrolase RppH | 5'-end-dependent mRNA decay | Gene expression and regulation | (Vasilyev and Serganov, 2015) |
| 267 | WP_012461483.1 | Fe | Cytoplasm | Glutaredoxin | Heme and iron cluster assembly biosynthesis | Metabolism | (Rouhier et al., 2010) |
| 268 | WP_012462321.1 | Fe | Cytoplasm | Glutaredoxin | Heme and iron cluster assembly biosynthesis | Metabolism | (Rouhier et al., 2010) |
| 269 | WP_012461099.1 | Mg | Inner Membrane | AAA domain/ParA | Chromosome partition | Gene expression and regulation | (Ogura and Wilkinson, 2001) |
| 270 | WP_012460749.1 | Mn, Ni, Cd | Cytoplasm | Integration host factor (IHF)-like DNA-binding domain | Candensation of bacterial chromosome | Gene expression and regulation | (Stonehouse et al., 2008) |
| 271 | WP_012461492.1 | Mg, Ca | Cytoplasm | Tubulin/FtsZ family, GTPase domain | Bacterial cell division | Gene expression and regulation | (Erickson et al., 2010) |
| 272 | WP_012460738.1 | Zn, Mg, Fe | Inner Membrane | Era-type guanine nucleotide-binding (G) domain | rRNA processing, cell division and metabolism | Metabolism | (Sullivan et al., 2000) |
| 273 | WP_012461532.1 | Mg, Ca | Cytoplasm | UDP-N-acetylenolpyruvoylglucosamine reductase (murB) | Biogenesis of cellwall peptidoglycan | Metabolism | (Jukič et al., 2019; Moraes et al., 2015; Munshi et al., 2013) |
| 274 | WP_012462032.1 | Ca | Cytoplasm | DNA polymerase III, beta chain | DNA replication | Gene expression and regulation | (Robinson et al., 2012; Vashishtha et al., 2016; Walker et al., 2000) |
| 275 | WP_012461643.1 | Mg, Ca, Zn, Mn | Cytoplasm | Diguanylate cyclase, GGDEF domain | Biosynthesis of c-di-GMP | Cell signaling | (Ryan et al., 2006) |
| 276 | WP_012462010.1 | Ca | Periplasm | TolB, N-terminal | Protein transport | Transport | (Loftus et al., 2006) |
| 277 | WP_012461701.1 | Fe | Cytoplasm | Radical SAM | Redical species generation by reductive cleavage of SAM using Fe-S cluster | Gene expression and regulation | (Broderick et al., 2014) |
| 278 | WP_012460808.1 | Fe | Cytoplasm | Radical SAM | Redical species generation by reductive cleavage of SAM using Fe-S cluster | Gene expression and regulation | (Broderick et al., 2014) |
| 279 | WP_012462252.1 | Ca | Inner Membrane | Glycoside hydrolase family 18, catalytic domain | Carbohydrate metabolic process | Metabolism | (Ardèvol and Rovira, 2015; Payne et al., 2012) |
| 280 | WP_012462219.1 | Zn | Cytoplasm | DNA recombination protein RecR | DNA repair and recombination | Gene expression and regulation | (Tang et al., 2014) |
| 281 | WP_012461246.1 | Mg, Zn | Cytoplasm | Phenylalanyl-tRNA synthetase | tRNA aminoacylation | Gene expression and regulation | (Biryukov et al., 1991) |
| 282 | WP_012461170.1 | Mg, Fe, Cd, Cu | Cytoplasm | Ferrochelatase | heme biosynthetic process | Metabolism | (Al-Karadaghi et al., 1997) |
| 283 | WP_012461920.1 | Mg, Ca, Zn | Cytoplasm | Alcohol dehydrogenase | Reversible oxidation of alcohol | Metabolism | (De Bolle et al., 1997; JÖRNVALL et al., 1987) |
| 284 | WP_012461450.1 | Ca | Cytoplasm | Fumarate lyase family | TCA: reversible hydration of fumaric acid to malate | Metabolism | (Puchegger et al., 1990) |
| 285 | WP_012460755.1 | Mg, Ca, Zn | Cytoplasm | Thioredoxin domain | Cell redox homeostasis | Metabolism | (Holmgren, 1985) |
| 286 | WP_012462041.1 | Mg | Cytoplasm | tRNA threonylcarbamoyl adenosine modification protein TsaE | tRNA threonylcarbamoyladenosine modification | Post translational modification | (Missoury et al., 2018) |
| 287 | WP_012461719.1 | Zn, Cu | Cytoplasm | Histidine triad (HIT) protein | Catalytic regulator of D-alanine dehydrogense | Metabolism | (Bardaweel et al., 2011) |
| 288 | WP_012462079.1 | Mg, Ca, Zn | Cytoplasm | tRNA dimethylallyltransferase | tRNA modification | Post translational modification | (Xie et al., 2007) |
| 289 | WP_012462317.1 | Mg, Zn | Cytoplasm | Zinc-finger, NAD-dependent DNA ligase C4-type | DNA repair, replication and recombination | Gene expression and regulation | (Lee et al., 2000) |
| 290 | WP_012461332.1 | Fe | Cytoplasm | Uracil-DNA glycosylase family 4 | Base excision repair | Gene expression and regulation | (Hinks et al., 2002) |
| 291 | WP_012462330.1 | Ca, Fe | Inner Membrane | Succinate dehydrogenase/fumarate reductase iron-sulphur protein | ETC: oxidation of succinate to fumarate in the cytoplasm and reduction of quinone to quinol in the membrane | Metabolism | (Cecchini et al., 2002) |
| 292 | WP_012461569.1 | Fe | Cytoplasm | Methylthiotransferase | catalyses a C-H to C-S bond conversion in the methylthiolation of tRNA | Post translational modification | (Broderick et al., 2014) |
| 293 | WP_012462195.1 | Fe | Cytoplasm | Methylthiotransferase | catalyses a C-H to C-S bond conversion in the methylthiolation of tRNA | Post translational modification | (Broderick et al., 2014) |
| 294 | WP_012461378.1 | Mg, Zn | Cytoplasm | Methioninyl-tRNA synthetase core domain | Methionyl-tRNA aminoacylation | Gene expression and regulation | (Schmitt et al., 1997) |
| 295 | WP_012462044.1 | Mg | Cytoplasm | tRNA(Ile)-lysidine synthase, N-terminal | tRNA modification | Gene expression and regulation | (Suzuki and Miyauchi, 2010) |
| 296 | WP_012461278.1 | Fe, Cu | Inner Membrane | Cytochrome c oxidase subunit III | Coupling between dioxygen reduction and proton pumping | Metabolism | (Belevich et al., 2006) |
| 297 | WP_012460730.1 | Mn, Zn | Cytoplasm | Prolyl-tRNA synthetase, class IIa, bacterial-type | tRNA aminoacylation | Gene expression and regulation | (Crepin et al., 2006) |
| 298 | WP_012460954.1 | Ca | Inner Membrane | Ppx/GppA phosphatase family | Stress response regulator and virulence | Stress response regulator | (Kristensen et al., 2004) |
| 299 | WP_012461726.1 | Mg, Ni | Cytoplasm | Ribosome-binding ATPase YchF/Obg-like ATPase 1 | Ribosome assembly | Gene expression and regulation | (KONG et al., 2016) |
| 300 | WP_012460882.1 | Mg, Ca, Mn, Fe | Cytoplasm | Tryptophan-tRNA ligase | tRNA aminoacylation | Gene expression and regulation | (Buddha and Crane, 2005) |
| 301 | WP_012461769.1 | Mg, Ca, Mn | Inner Membrane | Ribonuclease HI | Degradation of RNA in RNA.DNA hybrids | Gene expression and regulation | (Goedken and Marqusee, 1999) |
| 302 | WP_012460740.1 | Zn | Cytoplasm | Zinc-binding ribosomal protein | Translation and response to stress | Gene expression and regulation | (Owen et al., 2007) |
| 303 | WP_012461765.1 | Ca | Cytoplasm | NusB antitermination factor | Transcription antitermination | Gene expression and regulation | (Bonin et al., 2004) |
| 304 | WP_012461876.1 | Zn | Cytoplasm | Translation initiation factor IF-1 | Translational initiation | Gene expression and regulation | (Laursen et al., 2005) |
| 305 | WP_012462271.1 | Mg, Ca, Zn, Mn | Cytoplasm | Inorganic pyrophosphatase | Lipid metabolism | Metabolism | (Kurilova et al., 1984) |
| 306 | WP_012461003.1 | Mg | Cytoplasm | RNA polymerase sigma factor 54 interaction domain | Regulate expression of prokaryotic transcription genes | Gene expression and regulation | (Kazmierczak et al., 2005) |
| 307 | WP_045916150.1 | Mg, Zn | Cytoplasm | Cryptochrome/DNA photolyase class 1 | Light dependent DNA repair | Gene expression and regulation | (Kiontke et al., 2011) |
| 308 | WP_012461101.1 | Mg | Cytoplasm | Serine-tRNA synthetase | tRNA aminoacylation | Gene expression and regulation | (RIGLER et al., 1976) |
| 309 | WP_012461109.1 | Ca | Cytoplasm | Alpha/beta hydrolase family | Catalytic activity | Metabolism | (Holmquist, 2000) |
| 310 | WP_012461072.1 | Mg, Ca | Cytoplasm | Pyruvate, phosphate dikinase | Pyruvate metabolic process | Metabolism | (Zúñiga-Ripa et al., 2014) |
| 311 | WP_012461880.1 | Mg | Cytoplasm | Serine hydroxymethyltransferase | Glycine biosynthetic process | Metabolism | (Tanaka et al., 1981) |
| 312 | WP_012461549.1 | Mg, Mn | Cytoplasm | Deoxyuridine triphosphate nucleotidohydrolase | dUTP metabolic process | Metabolism | (Nguyen et al., 2005) |
| 313 | WP_012461442.1 | Mg | Cytoplasm | Guanylate kinase | Purine nucleotide metabolic process | Metabolism | (Liu et al., 2015) |
| 314 | WP_012461274.1 | Mg, Ca | Cytoplasm | 2-oxoacid dehydrogenase acyltransferase, catalytic domain | Conversion of alpha-keto acids to acyl-CoA | Metabolism | (Kumaran et al., 2013) |
| 315 | WP_012461868.1 | Mg, Zn | Cytoplasm | tRNA pseudouridine synthase II, TruB | RNA modification | Post translational modification | (Friedt et al., 2013) |
| 316 | WP_012462037.1 | Mn | Inner Membrane | UbiD decarboxylyase family | Ubiquinone biosynthesis | Metabolism | (Marshall et al., 2017) |
| 317 | WP_012461222.1 | Zn, Fe | Cytoplasm | NifU/ISC system FeS cluster assembly | Iron-sulphur cluster biosynthesis | Gene expression and regulation | (Roche et al., 2013) |
| 318 | WP_012461346.1 | Ca, Fe, Cd | Cytoplasm | Single-strand binding protein family | DNA repair, replication and recombination | Gene expression and regulation | (Meyer and Laine, 1990) |
| 319 | WP_012461612.1 | Mn | Cytoplasm | Ribosomal protein L16p/L10e | Translation and antimicrobial resistance | Gene expression and regulation | (Aarestrup and Jensen, 2000) |
| 320 | WP_012461804.1 | Zn | Cytoplasm | Transcription elongation factor, GreA/GreB, N-terminal | Induce nucleolytic activity of RNAP and response to stress | Gene expression and regulation | (Stepanova et al., 2007) |
| 321 | WP_012461438.1 | Mg, Ni | Cytoplasm | Acetylglutamate kinase | Arginine biosynthesis | Metabolism | (Ramón-Maiques et al., 2002) |

*** List of references used in Supplementary Table S3A**

Aarestrup, F. M., and Jensen, L. B. (2000). Presence of variations in ribosomal protein L16 corresponding to susceptibility of enterococci to oligosaccharides (avilamycin and evernimicin). *Antimicrobial agents and chemotherapy* 44, 3425–3427.

Aguirre, J. D., and Culotta, V. C. (2012). Battles with iron: manganese in oxidative stress protection. *Journal of Biological Chemistry* 287, 13541–13548.

Al-Karadaghi, S., Hansson, M., Nikonov, S., Jönsson, B., and Hederstedt, L. (1997). Crystal structure of ferrochelatase: the terminal enzyme in heme biosynthesis. *Structure* 5, 1501–1510.

Al-Khodor, S., Price, C. T., Kalia, A., and Kwaik, Y. A. (2010). Ankyrin-repeat containing proteins of microbes: a conserved structure with functional diversity. *Trends in microbiology* 18, 132.

Albrecht, R., Schütz, M., Oberhettinger, P., Faulstich, M., Bermejo, I., Rudel, T., et al. (2014). Structure of BamA, an essential factor in outer membrane protein biogenesis. *Acta Crystallographica Section D: Biological Crystallography* 70, 1779–1789.

Aoki, N., Ishii, Y., Tateda, K., Saga, T., Kimura, S., Kikuchi, Y., et al. (2010). Efficacy of calcium-EDTA as an inhibitor for metallo-β-lactamase in a mouse model of Pseudomonas aeruginosa pneumonia. *Antimicrobial agents and chemotherapy* 54, 4582–4588.

Ardèvol, A., and Rovira, C. (2015). Reaction mechanisms in carbohydrate-active enzymes: glycoside hydrolases and glycosyltransferases. Insights from ab initio quantum mechanics/molecular mechanics dynamic simulations. *Journal of the American Chemical Society* 137, 7528–7547.

Banecki, B., Liberek, K., Wall, D., Wawrzynów, A., Georgopoulos, C., Bertoli, E., et al. (1996). Structure-function analysis of the zinc finger region of the DnaJ molecular chaperone. *Journal of Biological Chemistry* 271, 14840–14848.

Banerjee, R., Dubois, D. Y., Gauthier, J., Lin, S. X., Roy, S., and Lapointe, J. (2004). The zinc-binding site of a class I aminoacyl-tRNA synthetase is a SWIM domain that modulates amino acid binding via the tRNA acceptor arm. *European Journal of Biochemistry* 271, 724–733. doi:10.1111/j.1432-1033.2003.03976.x.

Bang, S., Min, C.-K., Ha, N.-Y., Choi, M.-S., Kim, I.-S., Kim, Y.-S., et al. (2016). Inhibition of eukaryotic translation by tetratricopeptide-repeat proteins of Orientia tsutsugamushi. *Journal of Microbiology* 54, 136–144.

Bardaweel, S., Ghosh, B., Chou, T.-F., Sadowsky, M. J., and Wagner, C. R. (2011). E. coli histidine triad nucleotide binding protein 1 (ecHinT) is a catalytic regulator of D-alanine dehydrogenase (DadA) activity in vivo. *PloS one* 6, e20897.

Becker, A., Schlichting, I., Kabsch, W., Groche, D., Schultz, S., and Wagner, A. F. V. (1998). Iron center, substrate recognition and mechanism of peptide deformylase. *Nature Structural & Molecular Biology* 5, 1053.

Belevich, I., Verkhovsky, M. I., and Wikström, M. (2006). Proton-coupled electron transfer drives the proton pump of cytochrome c oxidase. *Nature* 440, 829.

Bennett, R. J., and West, S. C. (1995). Structural Analysis of the RuvC-Holliday Junction Complex Reveals and Unfolded Junction. *Journal of molecular biology* 252, 213–226.

Beyer, D., Kroll, H.-P., Endermann, R., Schiffer, G., Siegel, S., Bauser, M., et al. (2004). New class of bacterial phenylalanyl-tRNA synthetase inhibitors with high potency and broad-spectrum activity. *Antimicrobial agents and chemotherapy* 48, 525–532.

Biondi, R. M., Schneider, B., Passeron, E., and Passeron, S. (1998). Role of Mg2+ in nucleoside diphosphate kinase autophosphorylation. *Archives of biochemistry and biophysics* 353, 85–92.

Biryukov, A. I., Ankilova, V. N., and Lavrik, O. I. (1991). Diadenosine oligophosphates: peculiarities of synthesis by phenylalanyl-tRNA synthetases from E. coli MRE-600 and Thermus thermophilus HB8. in *Nucleic acids symposium series*, 19–20.

Bologna, F. P., Andreo, C. S., and Drincovich, M. F. (2007). Escherichia coli malic enzymes: two isoforms with substantial differences in kinetic properties, metabolic regulation, and structure. *Journal of bacteriology* 189, 5937–5946.

Bonin, I., Robelek, R., Benecke, H., Urlaub, H., Bacher, A., and Richter, G. (2004). Crystal structures of the antitermination factor NusB from Thermotoga maritima and implications for RNA binding. *Biochemical Journal* 383, 419–428.

Brandt, U., Kerscher, S., Dröse, S., Zwicker, K., and Zickermann, V. (2003). Proton pumping by NADH: ubiquinone oxidoreductase. A redox driven conformational change mechanism? *FEBS letters* 545, 9–17.

Broderick, J. B., Duffus, B. R., Duschene, K. S., and Shepard, E. M. (2014). Radical S-adenosylmethionine enzymes. *Chemical reviews* 114, 4229–4317.

Brzóska, K., Meczynska, S., and Kruszewski, M. (2006). Iron-sulfur cluster proteins: electron transfer and beyond. *ACTA BIOCHIMICA POLONICA-ENGLISH EDITION-* 53, 685.

Buddha, M. R., and Crane, B. R. (2005). Structures of Tryptophanyl-tRNA Synthetase II from Deinococcus radiodurans Bound to ATP and Tryptophan INSIGHT INTO SUBUNIT COOPERATIVITY AND DOMAIN MOTIONS LINKED TO CATALYSIS. *Journal of Biological Chemistry* 280, 31965–31973.

Buglino, J., Shen, V., Hakimian, P., and Lima, C. D. (2002). Structural and biochemical analysis of the Obg GTP binding protein. *Structure* 10, 1581–1592.

Callaghan, A. J., Marcaida, M. J., Stead, J. A., McDowall, K. J., Scott, W. G., and Luisi, B. F. (2005). Structure of Escherichia coli RNase E catalytic domain and implications for RNA turnover. *Nature* 437, 1187.

Camire, E. J., Grossman, J. D., Thole, G. J., Fleischman, N. M., and Perlstein, D. L. (2015). The yeast Nbp35-Cfd1 cytosolic iron-sulfur cluster scaffold is an ATPase. *Journal of Biological Chemistry* 290, 23793–23802.

CARR, A. C., PENZER, G. R., PLUMBRIDGE, J. A., and IGLOI, G. L. (1975). The effects of spermine and Mg2+ on the catalytic mechanism of isoleucine: tRNA ligase. *European journal of biochemistry* 54, 169–173.

Cascales, E., Lloubès, R., and Sturgis, J. N. (2001). The TolQ–TolR proteins energize TolA and share homologies with the flagellar motor proteins MotA–MotB. *Molecular microbiology* 42, 795–807.

Cecchini, G., Schröder, I., Gunsalus, R. P., and Maklashina, E. (2002). Succinate dehydrogenase and fumarate reductase from Escherichia coli. *Biochimica et Biophysica Acta (BBA)-Bioenergetics* 1553, 140–157.

Cerveny, L., Straskova, A., Dankova, V., Hartlova, A., Ceckova, M., Staud, F., et al. (2013). Tetratricopeptide repeat motifs in the world of bacterial pathogens: role in virulence mechanisms. *Infection and immunity* 81, 629–635.

Chaitanya, M., Babajan, B., Anuradha, C. M., Naveen, M., Rajasekhar, C., Madhusudana, P., et al. (2010). Exploring the molecular basis for selective binding of Mycobacterium tuberculosis Asp kinase toward its natural substrates and feedback inhibitors: a docking and molecular dynamics study. *Journal of molecular modeling* 16, 1357–1367.

Chakravarty, S., Melton, C. N., Bailin, A., Yahr, T. L., and Anderson, G. G. (2017). Pseudomonas aeruginosa magnesium transporter MgtE inhibits type III secretion system gene expression by stimulating rsmYZ transcription. *Journal of bacteriology* 199, e00268-17.

Chan, H.-C., Feng, X., Ko, T.-P., Huang, C.-H., Hu, Y., Zheng, Y., et al. (2014). Structure and inhibition of tuberculosinol synthase and decaprenyl diphosphate synthase from Mycobacterium tuberculosis. *Journal of the American Chemical Society* 136, 2892–2896.

Chang, Y., Bruni, R., Kloss, B., Assur, Z., Kloppmann, E., Rost, B., et al. (2014). Structural basis for a pH-sensitive calcium leak across membranes. *Science* 344, 1131–1135.

Chen, K., Roberts, E., and Luthey-Schulten, Z. (2009). Horizontal gene transfer of zinc and non-zinc forms of bacterial ribosomal protein S4. *BMC evolutionary biology* 9, 179.

Chen, X., Boonyalai, N., Lau, C., Thipayang, S., Xu, Y., Wright, M., et al. (2013). Multiple catalytic activities of Escherichia coli lysyl‐tRNA synthetase (LysU) are dissected by site‐directed mutagenesis. *The FEBS journal* 280, 102–114.

Chen, Y. C., Li, C. L., Hsiao, Y. Y., Duh, Y., and Yuan, H. S. (2014). Structure and function of TatD exonuclease in DNA repair. *Nucleic Acids Research* 42, 10776–10785. doi:10.1093/nar/gku732.

Choi, H.-P., Hong, J.-W., Rhee, K.-H., and Sung, H.-C. (2004). Cloning, expression, and characterization of 5-aminolevulinic acid synthase from Rhodopseudomonas palustris KUGB306. *FEMS microbiology letters* 236, 175–181.

Choi, Y. K., Jo, P. G., and Choi, C. Y. (2008). Cadmium affects the expression of heat shock protein 90 and metallothionein mRNA in the Pacific oyster, Crassostrea gigas. *Comparative Biochemistry and Physiology Part C: Toxicology & Pharmacology* 147, 286–292.

Cisneros, G. A., Perera, L., Schaaper, R. M., Pedersen, L. C., London, R. E., Pedersen, L. G., et al. (2009). Reaction mechanism of the ε subunit of E. coli DNA polymerase III: insights into active site metal coordination and catalytically significant residues. *Journal of the American Chemical Society* 131, 1550–1556.

Crepin, T., Yaremchuk, A., Tukalo, M., and Cusack, S. (2006). Structures of two bacterial prolyl-tRNA synthetases with and without a cis-editing domain. *Structure* 14, 1511–1525.

Curti, E., Smerdon, S. J., and Davis, E. O. (2007). Characterization of the helicase activity and substrate specificity of Mycobacterium tuberculosis UvrD. *Journal of bacteriology* 189, 1542–1555.

Davies, R. B., and Abraham, E. P. (1974). Metal cofactor requirement of β-lactamase II. *Biochemical Journal* 143, 129–135.

De Bolle, X., VINALS, C., Fastrez, J., and FEYTMANS, E. (1997). Bivalent cations stabilize yeast alcohol dehydrogenase I. *Biochemical Journal* 323, 409–413.

DIGNAM, J. D., DIGNAM, S. S., and BRUMLEY, L. L. (1991). Alanyl‐tRNA synthetase from Escherichia coli, Bombyx mori and Ratus ratus: Existence of common structural features. *European journal of biochemistry* 198, 201–210.

Domanico, P. L., and Tse-Dinh, Y. C. (1991). Mechanistic studies on E. coli DNA topoisomerase I: divalent ion effects. *Journal of inorganic biochemistry* 42, 87–96.

Eide, D. J. (2006). Zinc transporters and the cellular trafficking of zinc. *Biochimica et Biophysica Acta (BBA)-Molecular Cell Research* 1763, 711–722.

El-Housseiny, G. S., Aboulwafa, M. M., and Hassouna, N. A. (2010). Cytotoxic activities of some Escherichia coli isolates: Possible mechanisms and approaches for inhibition. *J Am Sci* 6, 10.

El Qaidi, S., Zhu, C., McDonald, P., Roy, A., Maity, P. K., Rane, D., et al. (2018). High-throughput screening for bacterial glycosyltransferase inhibitors. *Frontiers in cellular and infection microbiology* 8, 435.

Erickson, H. P., Anderson, D. E., and Osawa, M. (2010). FtsZ in bacterial cytokinesis: cytoskeleton and force generator all in one. *Microbiol. Mol. Biol. Rev.* 74, 504–528.

Fang, L., Huang, S., and Lin, K. (1997). High temperature induces the synthesis of heat-shock proteins and the elevation of intracellular calcium in the coral Acropora grandis. *Coral Reefs* 16, 127–131.

Finking, R., Solsbacher, J., Konz, D., Schobert, M., Schäfer, A., Jahn, D., et al. (2002). Characterization of a new type of phosphopantetheinyl transferase for fatty acid and siderophore synthesis in Pseudomonas aeruginosa. *Journal of Biological Chemistry* 277, 50293–50302.

Fioravanti, E., Adam, V., Munier-Lehmann, H., and Bourgeois, D. (2005). The crystal structure of Mycobacterium tuberculosis thymidylate kinase in complex with 3 ‘-azidodeoxythymidine monophosphate suggests a mechanism for competitive inhibition. *Biochemistry* 44, 130–137.

Focia, P. J., Alam, H., Lu, T., Ramirez, U. D., and Freymann, D. M. (2004). Novel protein and Mg2+ configurations in the Mg2+ GDP complex of the SRP GTPase ffh. *Proteins: Structure, Function, and Bioinformatics* 54, 222–230.

Friedrich, T., and Scheide, D. (2000). The respiratory complex I of bacteria, archaea and eukarya and its module common with membrane‐bound multisubunit hydrogenases. *FEBS letters* 479, 1–5.

Friedt, J., Leavens, F. M. V, Mercier, E., Wieden, H.-J., and Kothe, U. (2013). An arginine-aspartate network in the active site of bacterial TruB is critical for catalyzing pseudouridine formation. *Nucleic acids research* 42, 3857–3870.

Garmory, H. S., and Titball, R. W. (2004). ATP-binding cassette transporters are targets for the development of antibacterial vaccines and therapies. *Infection and immunity* 72, 6757–6763.

Gibson, M. M., Bagga, D. A., Miller, C. G., and Maguire, M. E. (1991). Magnesium transport in Salmonella typhimurium: the influence of new mutations conferring Co2+ resistance on the CorA Mg2+ transport system. *Molecular microbiology* 5, 2753–2762.

Goedken, E. R., and Marqusee, S. (1999). Metal binding and activation of the ribonuclease H domain from Moloney murine leukemia virus. *Protein engineering* 12, 975–980.

Gordon, G. L., and Doelle, H. W. (1976). Purification, Properties and Immunological Relationship of l (+)‐Lactate Dehydrogenase from Lactobacillus casei. *European journal of biochemistry* 67, 543–555.

Griepenburg, U., Blasczyk, K., Kappl, R., Hüttermann, J., and Auling, G. (1998). A Divalent Metal Site in the Small Subunit of the Manganese-Dependent Ribonucleotide Reductase of Corynebacterium a mmoniagenes. *Biochemistry* 37, 7992–7996.

Groisman, E. A., Hollands, K., Kriner, M. A., Lee, E.-J., Park, S.-Y., and Pontes, M. H. (2013). Bacterial Mg2+ homeostasis, transport, and virulence. *Annual review of genetics* 47, 625–646.

GRUNAU, J. A., KNIGHT, E., HART, S., and GUNSALUS, I. C. Succinyl Coenzyme A Synthetase from Escherichia coli.

Harlow, L. S., Kadziola, A., Jensen, K. F., and Larsen, S. (2004). Crystal structure of the phosphorolytic exoribonuclease RNase PH from Bacillus subtilis and implications for its quaternary structure and tRNA binding. *Protein science* 13, 668–677.

He, Q., Wang, F., Liu, S., Zhu, D., Cong, H., Gao, F., et al. (2016). Structural and biochemical insight into the mechanism of Rv2837c from Mycobacterium tuberculosis as a c-di-NMP phosphodiesterase. *Journal of Biological Chemistry* 291, 3668–3681.

Hensley, M. P., Tierney, D. L., and Crowder, M. W. (2011). Zn (II) binding to Escherichia coli 70S ribosomes. *Biochemistry* 50, 9937–9939.

Hinks, J. A., Evans, M. C. W., de Miguel, Y., Sartori, A. A., Jiricny, J., and Pearl, L. H. (2002). An iron-sulfur cluster in the family 4 uracil-DNA glycosylases. *Journal of Biological Chemistry* 277, 16936–16940.

Hizi, A., and Herzig, E. (2015). dUTPase: the frequently overlooked enzyme encoded by many retroviruses. *Retrovirology* 12, 70.

Hoganson, D. A., and Stahly, D. P. (1975). Regulation of dihydrodipicolinate synthase during growth and sporulation of Bacillus cereus. *Journal of bacteriology* 124, 1344–1350.

Holmgren, A. (1980). “Pyridine nucleotide—disulfide oxidoreductases,” in *Dehydrogenases* (Springer), 149–180.

Holmgren, A. (1985). Thioredoxin. *Annual review of biochemistry* 54, 237–271.

Holmquist, M. (2000). Alpha beta-hydrolase fold enzymes structures, functions and mechanisms. *Current Protein and Peptide Science* 1, 209–235.

Huynh, T. N., Luo, S., Pensinger, D., Sauer, J.-D., Tong, L., and Woodward, J. J. (2015). An HD-domain phosphodiesterase mediates cooperative hydrolysis of c-di-AMP to affect bacterial growth and virulence. *Proceedings of the National Academy of Sciences* 112, E747–E756.

Ito, T., Hemmi, H., Kataoka, K., Mukai, Y., and Yoshimura, T. (2008). A novel zinc-dependent D-serine dehydratase from Saccharomyces cerevisiae. *Biochemical Journal* 409, 399–406.

JENSEN, M., COOL, R. H., MORTENSEN, K. K., CLARK, B. F. C., and PARMEGGIANI, A. (1989). Structure‐function relationships of elongation factor Tu: Isolation and activity of the guanine‐nucleotide‐binding domain. *European journal of biochemistry* 182, 247–255.

Jenvert, R.-M., and Schiavone, L. H. (2007). The flexible N-terminal domain of ribosomal protein L11 from Escherichia coli is necessary for the activation of stringent factor. *Journal of molecular biology* 365, 764–772.

Jonna, V. R., Crona, M., Rofougaran, R., Lundin, D., Johansson, S., Brännström, K., et al. (2015). Diversity in overall activity regulation of ribonucleotide reductase. *Journal of Biological Chemistry* 290, 17339–17348.

JÖRNVALL, H., PERSSON, B., and JEFFERY, J. (1987). Characteristics of alcohol/polyol dehydrogenases: The zinc‐containing long‐chain alcohol dehydrogenases. *European Journal of Biochemistry* 167, 195–201.

Jude, F., Köhler, T., Branny, P., Perron, K., Mayer, M. P., Comte, R., et al. (2003). Posttranscriptional control of quorum-sensing-dependent virulence genes by DksA in Pseudomonas aeruginosa. *Journal of bacteriology* 185, 3558–3566.

Jukič, M., Gobec, S., and Sova, M. (2019). Reaching toward underexplored targets in antibacterial drug design. *Drug development research* 80, 6–10.

Julio, S. M., Heithoff, D. M., Provenzano, D., Klose, K. E., Sinsheimer, R. L., Low, D. A., et al. (2001). DNA Adenine Methylase Is Essential for Viability and Plays a Role in the Pathogenesis of Yersinia pseudotuberculosis andVibrio cholerae. *Infection and immunity* 69, 7610–7615.

KAHN, D., FROMANT, M., FAYAT, G., DESSEN, P., and BLANQUET, S. (1980). Methionyl‐transfer‐RNA transformylase from Escherichia coli: purification and characterisation. *European journal of biochemistry* 105, 489–497.

Kaur, G., Kumar, V., Arora, A., Tomar, A., Sur, R., and Dutta, D. (2017). Affected energy metabolism under manganese stress governs cellular toxicity. *Scientific reports* 7, 11645.

Kazmierczak, M. J., Wiedmann, M., and Boor, K. J. (2005). Alternative sigma factors and their roles in bacterial virulence. *Microbiology and Molecular Biology Reviews* 69, 527–543. doi:10.1128/MMBR.69.4.527.

Kerff, F., Petrella, S., Mercier, F., Sauvage, E., Herman, R., Pennartz, A., et al. (2010). Specific structural features of the N-acetylmuramoyl-L-alanine amidase AmiD from Escherichia coli and mechanistic implications for enzymes of this family. *Journal of molecular biology* 397, 249–259.

Keto-Timonen, R., Hietala, N., Palonen, E., Hakakorpi, A., Lindström, M., and Korkeala, H. (2016). Cold shock proteins: a minireview with special emphasis on Csp-family of enteropathogenic Yersinia. *Frontiers in microbiology* 7, 1151.

Kiontke, S., Geisselbrecht, Y., Pokorny, R., Carell, T., Batschauer, A., and Essen, L. (2011). Crystal structures of an archaeal class II DNA photolyase and its complex with UV‐damaged duplex DNA. *The EMBO journal* 30, 4437–4449.

KONG, M.-Y., YAN, K.-G., MA, C.-Y., and GAO, N. (2016). Distinct Binding and Enzymatic Activities of Two Ribosome 鄄 dependent NTPases YchF and YihA. *Progress in Biochemistry and Biophysics* 43, 570–578.

Kow, Y. W. (1989). Mechanism of action of Escherichia coli exonuclease III. *Biochemistry* 28, 3280–3287.

Kristensen, O., Laurberg, M., Liljas, A., Kastrup, J. S., and Gajhede, M. (2004). Structural characterization of the stringent response related exopolyphosphatase/guanosine pentaphosphate phosphohydrolase protein family. *Biochemistry* 43, 8894–8900.

Kumaran, S., Patel, M., and Jordan, F. (2013). Nuclear Magnetic Resonance Approaches in the Study of 2-Oxo Acid Dehydrogenase Multienzyme Complexes—A Literature Review. *Molecules* 18, 11873–11903.

Kurilova, S. A., Bogdanova, A. V, Nazarova, T. I., and Avaeva, S. M. (1984). Changes in the E. coli inorganic pyrophosphatase activity on interaction with magnesium, zinc, calcium and fluoride ions. *Bioorganicheskaya Khimiya* 10, 1153–1160.

Kuron, A., Korycka-Machala, M., Brzostek, A., Nowosielski, M., Doherty, A., Dziadek, B., et al. (2014). Evaluation of DNA primase DnaG as a potential target for antibiotics. *Antimicrobial agents and chemotherapy* 58, 1699–1706.

Kutchma, A. J., Hoang, T. T., and Schweizer, H. P. (1999). Characterization of a Pseudomonas aeruginosa fatty acid biosynthetic gene cluster: purification of acyl carrier protein (ACP) and malonyl-coenzyme A: ACP transacylase (FabD). *Journal of bacteriology* 181, 5498–5504.

Kuznetsova, E., Nocek, B., Brown, G., Makarova, K. S., Flick, R., Wolf, Y. I., et al. (2015). Functional diversity of haloacid dehalogenase superfamily phosphatases from Saccharomyces cerevisiae biochemical, structural, and evolutionary insights. *Journal of Biological Chemistry* 290, 18678–18698.

Kwak, J. H., Shin, K., Woo, J.-S., Kim, M. K., Kim, S. Il, Eom, S. H., et al. (2002). Expression, purification, and crystallization of glutamyl-tRNA (Gln) specific amidotransferase from Bacillus stearothermophilus. *Molecules and cells* 14, 374–381.

Lacher, K., and Schafer, G. (1993). Archaebacterial adenylate kinase from the thermoacidophile Sulfolobus acidocaldarius: purification, characterization, and partial sequence. *Archives of biochemistry and biophysics* 302, 391–397.

Lai, C.-Y., and Cronan, J. E. (2003). β-Ketoacyl-acyl carrier protein synthase III (FabH) is essential for bacterial fatty acid synthesis. *Journal of Biological Chemistry* 278, 51494–51503.

Lamb, H. K., Thompson, P., Elliott, C., Charles, I. G., Richards, J., Lockyer, M., et al. (2007). Functional analysis of the GTPases EngA and YhbZ encoded by Salmonella typhimurium. *Protein science* 16, 2391–2402.

Langklotz, S., Baumann, U., and Narberhaus, F. (2012). Structure and function of the bacterial AAA protease FtsH. *Biochimica et Biophysica Acta (BBA)-Molecular Cell Research* 1823, 40–48.

Laursen, B. S., Sørensen, H. P., Mortensen, K. K., and Sperling-Petersen, H. U. (2005). Initiation of protein synthesis in bacteria. *Microbiol. Mol. Biol. Rev.* 69, 101–123.

Lawal, A., Jejelowo, O., Chopra, A. K., and Rosenzweig, J. A. (2011). Ribonucleases and bacterial virulence. *Microbial biotechnology* 4, 558–571.

Lebbink, J. H. G., Fish, A., Reumer, A., Natrajan, G., Winterwerp, H. H. K., and Sixma, T. K. (2010). Magnesium coordination controls the molecular switch function of DNA mismatch repair protein MutS. *Journal of Biological Chemistry* 285, 13131–13141.

Lee, J. Y., Chang, C., Song, H. K., Moon, J., Yang, J. K., Kim, H., et al. (2000). Crystal structure of NAD+‐dependent DNA ligase: modular architecture and functional implications. *The EMBO journal* 19, 1119–1129.

Lee, S.-H., Taguchi, H., Yoshimura, E., Minagawa, E., Kaminogawa, S., Ohta, T., et al. (1994). Carboxypeptidase Taq, a thermostable zinc enzyme, from Thermus aquaticus YT-1: molecular cloning, sequencing, and expression of the encoding gene in Escherichia coli. *Bioscience, biotechnology, and biochemistry* 58, 1490–1495.

Liew, S. M., Tay, S. T., and Puthucheary, S. D. (2013). Enzymatic and molecular characterisation of leucine aminopeptidase of Burkholderia pseudomallei. *BMC microbiology* 13, 110.

Liu, K., Myers, A. R., Pisithkul, T., Claas, K. R., Satyshur, K. A., Amador-Noguez, D., et al. (2015). Molecular mechanism and evolution of guanylate kinase regulation by (p) ppGpp. *Molecular cell* 57, 735–749.

Lloyd, A. J., Brandish, P. E., Gilbey, A. M., and Bugg, T. D. H. (2004). Phospho-N-acetyl-muramyl-pentapeptide translocase from Escherichia coli: catalytic role of conserved aspartic acid residues. *Journal of bacteriology* 186, 1747–1757.

Loftus, S. R., Walker, D., Maté, M. J., Bonsor, D. A., James, R., Moore, G. R., et al. (2006). Competitive recruitment of the periplasmic translocation portal TolB by a natively disordered domain of colicin E9. *Proceedings of the National Academy of Sciences* 103, 12353–12358.

Lu, J., Vlamis-Gardikas, A., Kandasamy, K., Zhao, R., Gustafsson, T. N., Engstrand, L., et al. (2013). Inhibition of bacterial thioredoxin reductase: an antibiotic mechanism targeting bacteria lacking glutathione. *The FASEB Journal* 27, 1394–1403.

Lusetti, S. L., Shaw, J. J., and Cox, M. M. (2003). Magnesium ion-dependent activation of the RecA protein involves the C terminus. *Journal of Biological Chemistry* 278, 16381–16388.

Ma, Z., Jacobsen, F. E., and Giedroc, D. P. (2009). Metal transporters and metal sensors: how coordination chemistry controls bacterial metal homeostasis. *Chemical reviews* 109, 4644.

Maher, M. J., Akimoto, S., Iwata, M., Nagata, K., Hori, Y., Yoshida, M., et al. (2009). Crystal structure of A3B3 complex of V‐ATPase from Thermus thermophilus. *The EMBO journal* 28, 3771–3779.

Marquis, R. E., Mayzel, K., and Carstensen, E. L. (1976). Cation exchange in cell walls of gram-positive bacteria. *Canadian journal of microbiology* 22, 975–982.

Marshall, S. A., Fisher, K., Cheallaigh, A. N., White, M. D., Payne, K. A. P., Parker, D. A., et al. (2017). Oxidative maturation and structural characterization of prenylated FMN binding by UbiD, a decarboxylase involved in bacterial ubiquinone biosynthesis. *Journal of Biological Chemistry* 292, 4623–4637.

Mathur, D., Malik, G., and Garg, L. C. (2006). Biochemical and functional characterization of triosephosphate isomerase from Mycobacterium tuberculosis H37Rv. *FEMS microbiology letters* 263, 229–235.

McEwan, A. R., Raab, A., Kelly, S. M., Feldmann, J., and Smith, M. C. M. (2011). Zinc is essential for high-affinity DNA binding and recombinase activity of φ C31 integrase. *Nucleic acids research* 39, 6137–6147.

Meier, C., Carter, L. G., Winter, G., Owens, R. J., Stuart, D. I., and Esnouf, R. M. (2007). Structure of 5-formyltetrahydrofolate cyclo-ligase from Bacillus anthracis (BA4489). *Acta Crystallographica Section F: Structural Biology and Crystallization Communications* 63, 168–172.

Meyer, R. R., and Laine, P. S. (1990). The single-stranded DNA-binding protein of Escherichia coli. *Microbiology and Molecular Biology Reviews* 54, 342–380.

Meyer, S., Scrima, A., Versees, W., and Wittinghofer, A. (2008). Crystal structures of the conserved tRNA-modifying enzyme GidA: implications for its interaction with MnmE and substrate. *Journal of molecular biology* 380, 532–547.

Mihara, H., and Esaki, N. (2002). Bacterial cysteine desulfurases: their function and mechanisms. *Applied microbiology and biotechnology* 60, 12–23.

Missoury, S., Plancqueel, S., Li de la Sierra-Gallay, I., Zhang, W., Liger, D., Durand, D., et al. (2018). The structure of the TsaB/TsaD/TsaE complex reveals an unexpected mechanism for the bacterial t6A tRNA-modification. *Nucleic acids research* 46, 5850–5860.

Mizutani, K., Machida, Y., Unzai, S., Park, S.-Y., and Tame, J. R. H. (2004). Crystal structures of the catalytic domains of pseudouridine synthases RluC and RluD from Escherichia coli. *Biochemistry* 43, 4454–4463.

Moraes, G. L., Gomes, G. C., De Sousa, P. R. M., Alves, C. N., Govender, T., Kruger, H. G., et al. (2015). Structural and functional features of enzymes of Mycobacterium tuberculosis peptidoglycan biosynthesis as targets for drug development. *Tuberculosis* 95, 95–111.

Mowbray, S., Kathiravan, M., Pandey, A., and Odell, L. (2014). Inhibition of glutamine synthetase: a potential drug target in Mycobacterium tuberculosis. *Molecules* 19, 13161–13176.

Munshi, T., Gupta, A., Evangelopoulos, D., Guzman, J. D., Gibbons, S., Keep, N. H., et al. (2013). Characterisation of ATP-dependent Mur ligases involved in the biogenesis of cell wall peptidoglycan in Mycobacterium tuberculosis. *PloS one* 8, e60143.

Nakamura, A., Sheppard, K., Yamane, J., Yao, M., Söll, D., and Tanaka, I. (2009). Two distinct regions in Staphylococcus aureus GatCAB guarantee accurate tRNA recognition. *Nucleic acids research* 38, 672–682.

Neupane, D. P., Avalos, D., Fullam, S., Roychowdhury, H., and Yukl, E. T. (2017). Mechanisms of zinc binding to the solute-binding protein AztC and transfer from the metallochaperone AztD. *Journal of Biological Chemistry* 292, 17496–17505.

Nguyen, C., Kasinathan, G., Leal-Cortijo, I., Musso-Buendia, A., Kaiser, M., Brun, R., et al. (2005). Deoxyuridine triphosphate nucleotidohydrolase as a potential antiparasitic drug target. *Journal of medicinal chemistry* 48, 5942–5954.

Niefind, K., Müller, J., Riebel, B., Hummel, W., and Schomburg, D. (2003). The crystal structure of R-specific alcohol dehydrogenase from Lactobacillus brevis suggests the structural basis of its metal dependency. *Journal of molecular biology* 327, 317–328.

Nikonov, O., Stolboushkina, E., Nikulin, A., Hasenöhrl, D., Bläsi, U., Manstein, D. J., et al. (2007). New insights into the interactions of the translation initiation factor 2 from archaea with guanine nucleotides and initiator tRNA. *Journal of molecular biology* 373, 328–336.

Nisa, S., Blokpoel, M. C. J., Robertson, B. D., Tyndall, J. D. A., Lun, S., Bishai, W. R., et al. (2010). Targeting the chromosome partitioning protein ParA in tuberculosis drug discovery. *Journal of antimicrobial chemotherapy* 65, 2347–2358.

Nurmohamed, S., Vaidialingam, B., Callaghan, A. J., and Luisi, B. F. (2009). Crystal structure of Escherichia coli polynucleotide phosphorylase core bound to RNase E, RNA and manganese: implications for catalytic mechanism and RNA degradosome assembly. *Journal of molecular biology* 389, 17–33.

Ogura, T., and Wilkinson, A. J. (2001). AAA+ superfamily ATPases: common structure–diverse function. *Genes to Cells* 6, 575–597.

Owen, G. A., Pascoe, B., Kallifidas, D., and Paget, M. S. B. (2007). Zinc-responsive regulation of alternative ribosomal protein genes in Streptomyces coelicolor involves Zur and σR. *Journal of bacteriology* 189, 4078–4086.

Palló, A., Oláh, J., Gráczer, É., Merli, A., Závodszky, P., Weiss, M. S., et al. (2014). Structural and energetic basis of isopropylmalate dehydrogenase enzyme catalysis. *The FEBS journal* 281, 5063–5076.

Palzkill, T. (2013). Metallo‐β‐lactamase structure and function. *Annals of the New York Academy of Sciences* 1277, 91–104.

Payne, C. M., Baban, J., Horn, S. J., Backe, P. H., Arvai, A. S., Dalhus, B., et al. (2012). Hallmarks of processivity in glycoside hydrolases from crystallographic and computational studies of the Serratia marcescens chitinases. *Journal of Biological Chemistry* 287, 36322–36330.

Pearce, F. G., Hudson, A. O., Loomes, K., and Dobson, R. C. J. (2017). “Dihydrodipicolinate synthase: structure, dynamics, function, and evolution,” in *Macromolecular Protein Complexes* (Springer), 271–289.

Petrov, A. S., Bernier, C. R., Hsiao, C., Okafor, C. D., Tannenbaum, E., Stern, J., et al. (2012). RNA–magnesium–protein interactions in large ribosomal subunit. *The Journal of Physical Chemistry B* 116, 8113–8120.

Prado, S., Villarroya, M., Medina, M., and Armengod, M.-E. (2013). The tRNA-modifying function of MnmE is controlled by post-hydrolysis steps of its GTPase cycle. *Nucleic acids research* 41, 6190–6208.

Puchegger, S., Redl, B., and Stöffler, G. (1990). Purification and properties of a thermostable fumarate hydratase from the archaeobacterium Sulfolobus solfataricus. *Microbiology* 136, 1537–1541.

Ramón-Maiques, S., Marina, A., Gil-Ortiz, F., Fita, I., and Rubio, V. (2002). Structure of acetylglutamate kinase, a key enzyme for arginine biosynthesis and a prototype for the amino acid kinase enzyme family, during catalysis. *Structure* 10, 329–342.

Rawlings, N. D., and Barrett, A. J. (1995). Evolutionary families of metallopeptidases. *Methods in Enzymology* 248, 183–228. doi:10.1016/0076-6879(95)48015-3.

Reddy, G. K., and Wendisch, V. F. (2014). Characterization of 3-phosphoglycerate kinase from Corynebacterium glutamicum and its impact on amino acid production. *BMC microbiology* 14, 54.

Rensing, C., and Grass, G. (2003). Escherichia coli mechanisms of copper homeostasis in a changing environment. *FEMS microbiology reviews* 27, 197–213.

Ricagno, S., De Rosa, M., Aliverti, A., Zanetti, G., and Bolognesi, M. (2007). The crystal structure of FdxA, a 7Fe ferredoxin from Mycobacterium smegmatis. *Biochemical and biophysical research communications* 360, 97–102.

RIGLER, R., PACHMANN, U., HIRSCH, R., and ZACHAU, H. G. (1976). On the Interaction of Seryl‐tRNA Synthetase with tRNASer: A Contribution to the Problem of Synthetase‐tRNA Recognition. *European journal of biochemistry* 65, 307–315.

Roberts, M. M., Coker, A. R., Fossati, G., Mascagni, P., Coates, A. R. M., and Wood, S. P. (2003). Mycobacterium tuberculosis chaperonin 10 heptamers self-associate through their biologically active loops. *Journal of bacteriology* 185, 4172–4185.

Robinson, A., J Causer, R., and E Dixon, N. (2012). Architecture and conservation of the bacterial DNA replication machinery, an underexploited drug target. *Current drug targets* 13, 352–372.

Roche, B., Aussel, L., Ezraty, B., Mandin, P., Py, B., and Barras, F. (2013). Reprint of: Iron/sulfur proteins biogenesis in prokaryotes: formation, regulation and diversity. *Biochimica et Biophysica Acta (BBA)-Bioenergetics* 1827, 923–937.

Rodríguez-Zavala, J. S., Pardo, J. P., and Moreno-Sánchez, R. (2000). Modulation of 2-oxoglutarate dehydrogenase complex by inorganic phosphate, Mg2+, and other effectors. *Archives of biochemistry and biophysics* 379, 78–84.

Rollin‐Genetet, F., Berthomieu, C., Davin, A., and Quéméneur, E. (2004). Escherichia coli thioredoxin inhibition by cadmium: two mutually exclusive binding sites involving Cys32 and Asp26. *European journal of biochemistry* 271, 1299–1309.

Rouhier, N., Couturier, J., Johnson, M. K., and Jacquot, J.-P. (2010). Glutaredoxins: roles in iron homeostasis. *Trends in biochemical sciences* 35, 43–52.

Roujeinikova, A., Baldock, C., Simon, W. J., Gilroy, J., Baker, P. J., Stuitje, A. R., et al. (2002). X-ray crystallographic studies on butyryl-ACP reveal flexibility of the structure around a putative acyl chain binding site. *Structure* 10, 825–835.

Rozeboom, H. J., Yu, S., Mikkelsen, R., Nikolaev, I., Mulder, H. J., and Dijkstra, B. W. (2015). Crystal structure of quinone‐dependent alcohol dehydrogenase from P seudogluconobacter saccharoketogenes. A versatile dehydrogenase oxidizing alcohols and carbohydrates. *Protein Science* 24, 2044–2054.

Ryan, R. P., Fouhy, Y., Lucey, J. F., and Dow, J. M. (2006). Cyclic di-GMP signaling in bacteria: recent advances and new puzzles. *Journal of bacteriology* 188, 8327–8334.

Schandel, K. A., Muller, M. M., and Webster, R. E. (1992). Localization of TraC, a protein involved in assembly of the F conjugative pilus. *Journal of bacteriology* 174, 3800–3806.

Schmid, J., Heider, D., Wendel, N. J., Sperl, N., and Sieber, V. (2016). Bacterial glycosyltransferases: challenges and opportunities of a highly diverse enzyme class toward tailoring natural products. *Frontiers in microbiology* 7, 182.

Schmidt, A., Schiesswohl, M., Völker, U., Hecker, M., and Schumann, W. (1992). Cloning, sequencing, mapping, and transcriptional analysis of the groESL operon from Bacillus subtilis. *Journal of bacteriology* 174, 3993–3999.

Schmitt, E., Panvert, M., Mechulam, Y., and Blanquet, S. (1997). General Structure/Function Properties of Microbial Methionyl‐Trna Synthetases. *European journal of biochemistry* 246, 539–547.

Schobert, B. (1998). Do ATP4− and Mg2+ bind stepwise to the F1‐ATPase of Halobacterium saccharovorum? *European journal of biochemistry* 254, 363–370.

Schuldt, L., Weyand, S., Kefala, G., and Weiss, M. S. (2009). The three-dimensional Structure of a mycobacterial DapD provides insights into DapD diversity and reveals unexpected particulars about the enzymatic mechanism. *Journal of molecular biology* 389, 863–879.

Selvy, P. E., Lavieri, R. R., Lindsley, C. W., and Brown, H. A. (2011). Phospholipase D: enzymology, functionality, and chemical modulation. *Chemical reviews* 111, 6064–6119.

Sissi, C., and Palumbo, M. (2009). Effects of magnesium and related divalent metal ions in topoisomerase structure and function. *Nucleic acids research* 37, 702–711.

Sloof, P., Hunter, J. B., Garrett, R. A., and Branlant, C. (1978). RNA-RNA interactions in the binding site of protein L24 on 23S ribosomal RNA of Escherichia coli: 1. Evidence for their occurrence between widely separated sequence regions. *Nucleic acids research* 5, 3503–3514.

Sola, M., Drew, D. L., Blanco, A. G., Gomis-Rüth, F. X., and Coll, M. (2006). The cofactor-induced pre-active conformation in PhoB. *Acta Crystallographica Section D: Biological Crystallography* 62, 1046–1057.

Soni, R. K., Mehra, P., Choudhury, N. R., Mukhopadhyay, G., and Dhar, S. K. (2003). Functional characterization of Helicobacter pylori DnaB helicase. *Nucleic acids research* 31, 6828–6840.

Soufo, H. J. D., Reimold, C., Linne, U., Knust, T., Gescher, J., and Graumann, P. L. (2010). Bacterial translation elongation factor EF-Tu interacts and colocalizes with actin-like MreB protein. *Proceedings of the National Academy of Sciences* 107, 3163–3168.

Spencer, P., and Jordan, P. M. (1993). Purification and characterization of 5-aminolaevulinic acid dehydratase from Escherichia coli and a study of the reactive thiols at the metal-binding domain. *Biochemical Journal* 290, 279–287.

Stepanova, E., Lee, J., Ozerova, M., Semenova, E., Datsenko, K., Wanner, B. L., et al. (2007). Analysis of promoter targets for Escherichia coli transcription elongation factor GreA in vivo and in vitro. *Journal of bacteriology* 189, 8772–8785.

Stonehouse, E., Kovacikova, G., Taylor, R. K., and Skorupski, K. (2008). Integration host factor positively regulates virulence gene expression in Vibrio cholerae. *Journal of bacteriology* 190, 4736–4748.

Sturton, R. G., and Brindley, D. N. (1977). Factors controlling the activities of phosphatidate phosphohydrolase and phosphatidate cytidylyltransferase. The effects of chlorpromazine, demethylimipramine, cinchocaine, norfenfluramine, mepyramine and magnesium ions. *Biochemical Journal* 162, 25–32.

Suksomtip, M., Liu, P., Anderson, T., Tungpradabkul, S., Wood, D. W., and Nester, E. W. (2005). Citrate synthase mutants of Agrobacterium are attenuated in virulence and display reduced vir gene induction. *Journal of bacteriology* 187, 4844–4852.

Sullivan, S. M., Mishra, R., Neubig, R. R., and Maddock, J. R. (2000). Analysis of guanine nucleotide binding and exchange kinetics of the Escherichia coli GTPase Era. *Journal of Bacteriology* 182, 3460–3466.

Sun, W., Pertzev, A., and Nicholson, A. W. (2005). Catalytic mechanism of Escherichia coli ribonuclease III: kinetic and inhibitor evidence for the involvement of two magnesium ions in RNA phosphodiester hydrolysis. *Nucleic acids research*33, 807–815.

Suzuki, T., and Miyauchi, K. (2010). Discovery and characterization of tRNAIle lysidine synthetase (TilS). *FEBS letters* 584, 272–277.

Tanaka, Y., Ōmura, S., Araki, K., and Nakayama, K. (1981). Derepression of glycine decarboxylase synthesis by magnesium phosphate in Nocardia butanica. *Agricultural and Biological Chemistry* 45, 2661–2664.

Tang, Q., Liu, Y.-P., Yan, X.-X., and Liang, D.-C. (2014). Structural and functional characterization of Cys4 zinc finger motif in the recombination mediator protein RecR. *DNA repair* 24, 10–14.

Tanimoto, K., Higashi, N., Nishioka, M., Ishikawa, K., and Taya, M. (2008). Characterization of thermostable aminoacylase from hyperthermophilic archaeon Pyrococcus horikoshii. *The FEBS journal* 275, 1140–1149.

Thompson, A. K., Gray, J., Liu, A., and Hosler, J. P. (2012). The roles of Rhodobacter sphaeroides copper chaperones PCuAC and Sco (PrrC) in the assembly of the copper centers of the aa3-type and the cbb3-type cytochrome c oxidases. *Biochimica et Biophysica Acta (BBA)-Bioenergetics* 1817, 955–964.

Thompson, D., and Simonson, T. (2006). Molecular dynamics simulations show that bound Mg2+ contributes to amino acid and aminoacyl adenylate binding specificity in aspartyl-tRNA synthetase through long range electrostatic interactions. *Journal of Biological Chemistry* 281, 23792–23803.

Trasnea, P., Utz, M., Khalfaoui‐Hassani, B., Lagies, S., Daldal, F., and Koch, H. (2016). Cooperation between two periplasmic copper chaperones is required for full activity of the cbb3‐type cytochrome c oxidase and copper homeostasis in Rhodobacter capsulatus. *Molecular microbiology* 100, 345–361.

Trumpower, B. L. (1990). Cytochrome bc1 complexes of microorganisms. *Microbiology and Molecular Biology Reviews* 54, 101–129.

Tsutakawa, S. E., Shin, D. S., Mol, C. D., Izumi, T., Arvai, A. S., Mantha, A. K., et al. (2013). Conserved structural chemistry for incision activity in structurally non-homologous apurinic/apyrimidinic endonuclease APE1 and endonuclease IV DNA repair enzymes. *Journal of Biological Chemistry* 288, 8445–8455.

Tu, H.-M., Yang, Y.-S., Li, Y., and Wang, E.-D. (2000). *Effect of rare earth ions on kinetic properties of Escherichia coli leucyl-tRNA synthetase*.

Tytgat, I., Colacino, E., Tulkens, P. M., Poupaert, J. H., Prévost, M., and Van Bambeke, F. (2009). DD-ligases as a potential target for antibiotics: past, present and future. *Current medicinal chemistry* 16, 2566–2580.

Ugalde, S. O., Boot, M., Commandeur, J. N. M., Jennings, P., Bitter, W., and Vos, J. C. (2019). Function, essentiality, and expression of cytochrome P450 enzymes and their cognate redox partners in Mycobacterium tuberculosis: are they drug targets? *Applied Microbiology and Biotechnology*, 1–18.

Van Houten, B. (1990). Nucleotide excision repair in Escherichia coli. *Microbiology and Molecular Biology Reviews* 54, 18–51.

Vashishtha, A. K., Wang, J., and Konigsberg, W. H. (2016). Different divalent cations alter the kinetics and fidelity of DNA polymerases. *Journal of Biological Chemistry* 291, 20869–20875.

Vasilyev, N., and Serganov, A. (2015). Structures of RNA complexes with the Escherichia coli RNA pyrophosphohydrolase RppH unveil the basis for specific 5′-end-dependent mRNA decay. *Journal of Biological Chemistry* 290, 9487–9499.

Vercruysse, M., Köhrer, C., Davies, B. W., Arnold, M. F. F., Mekalanos, J. J., RajBhandary, U. L., et al. (2014). The highly conserved bacterial RNase YbeY is essential in Vibrio cholerae, playing a critical role in virulence, stress regulation, and RNA processing. *PLoS pathogens* 10, e1004175.

VieBrock, L., Evans, S. M., Beyer, A. R., Larson, C. L., Beare, P. A., Ge, H., et al. (2015). Orientia tsutsugamushi ankyrin repeat-containing protein family members are Type 1 secretion system substrates that traffic to the host cell endoplasmic reticulum. *Frontiers in cellular and infection microbiology* 4, 186.

Villa, T. G., and Vinas, M. (2016). *New weapons to control bacterial growth*. Springer.

Waksman, G., and Orlova, E. V (2014). Structural organisation of the type IV secretion systems. *Current opinion in microbiology* 17, 24–31.

Walker, J. R., Hervas, C., Ross, J. D., Blinkova, A., Walbridge, M. J., Pumarega, E. J., et al. (2000). Escherichia coli DNA polymerase III τ-and γ-subunit conserved residues required for activity in vivo and in vitro. *Journal of bacteriology* 182, 6106–6113.

Wallrapp, F. H., Pan, J.-J., Ramamoorthy, G., Almonacid, D. E., Hillerich, B. S., Seidel, R., et al. (2013). Prediction of function for the polyprenyl transferase subgroup in the isoprenoid synthase superfamily. *Proceedings of the National Academy of Sciences* 110, E1196–E1202.

Wang, J., and Julin, D. A. (2004). DNA helicase activity of the RecD protein from Deinococcus radiodurans. *Journal of Biological Chemistry* 279, 52024–52032.

Windgassen, T. A., Wessel, S. R., Bhattacharyya, B., and Keck, J. L. (2017). Mechanisms of bacterial DNA replication restart. *Nucleic acids research* 46, 504–519.

Wittinghofer, A., Guariguata, R., and Leberman, R. (1983). Bacterial elongation factor Ts: isolation and reactivity with elongation factor Tu. *Journal of bacteriology* 153, 1266–1271.

Wolf, J., Gerber, A. P., and Keller, W. (2002). tadA, an essential tRNA‐specific adenosine deaminase from Escherichia coli. *The EMBO journal* 21, 3841–3851.

Wolf, N. M., Abad-Zapatero, C., Johnson, M. E., and Fung, L.-M. (2014). Structures of SAICAR synthetase (PurC) from Streptococcus pneumoniae with ADP, Mg2+, AIR and Asp. *Acta Crystallographica Section D: Biological Crystallography* 70, 841–850.

Wood, Z. A., Schröder, E., Harris, J. R., and Poole, L. B. (2003). Structure, mechanism and regulation of peroxiredoxins. *Trends in biochemical sciences* 28, 32–40.

Xie, W., Zhou, C., and Huang, R. H. (2007). Structure of tRNA dimethylallyltransferase: RNA modification through a channel. *Journal of molecular biology* 367, 872–881.

Yang, J., Peng, Q., Chen, Z., Deng, C., Shu, C., Zhang, J., et al. (2013). Transcriptional regulation and characteristics of a novel N-acetylmuramoyl-L-alanine amidase gene involved in Bacillus thuringiensis mother cell lysis. *Journal of bacteriology* 195, 2887–2897.

Yeh, A. P., Chatelet, C., Soltis, S. M., Kuhn, P., Meyer, J., and Rees, D. C. (2000). Structure of a thioredoxin-like [2Fe-2S] ferredoxin from Aquifex aeolicus. *Journal of molecular biology* 300, 587–595.

Yehudai-Resheff, S., and Schuster, G. (2000). Characterization of the E. coli poly (A) polymerase: nucleotide specificity, RNA-binding affinities and RNA structure dependence. *Nucleic acids research* 28, 1139–1144.

Yoon, K. P., Misra, T. K., and Silver, S. (1991). Regulation of the cadA cadmium resistance determinant of Staphylococcus aureus plasmid pI258. *Journal of bacteriology* 173, 7643–7649.

Young, P. G., Smith, C. A., Metcalf, P., and Baker, E. N. (2008). Structures of Mycobacterium tuberculosis folylpolyglutamate synthase complexed with ADP and AMPPCP. *Acta Crystallographica Section D: Biological Crystallography* 64, 745–753.

Zhang, C.-M., Christian, T., Newberry, K. J., Perona, J. J., and Hou, Y.-M. (2003). Zinc-mediated amino acid discrimination in cysteinyl-tRNA synthetase. *Journal of molecular biology* 327, 911–917.

Zheng, L., Yang, J., Landwehr, C., Fan, F., and Ji, Y. (2005). Identification of an essential glycoprotease in Staphylococcus aureus. *FEMS microbiology letters* 245, 279–285.

Zhu, L., Lin, J., Kuang, Z., Vidal, J. E., and Lau, G. W. (2015). Deletion analysis of S treptococcus pneumoniae late competence genes distinguishes virulence determinants that are dependent or independent of competence induction. *Molecular microbiology* 97, 151–165.

Zúñiga-Ripa, A., Barbier, T., Conde-Álvarez, R., Martínez-Gómez, E., Palacios-Chaves, L., Gil-Ramírez, Y., et al. (2014). Brucella abortus depends on pyruvate phosphate dikinase and malic enzyme but not on Fbp and GlpX fructose-1, 6-bisphosphatases for full virulence in laboratory models. *Journal of bacteriology* 196, 3045–3057.

**Supplementary Table S3B. GO enriched biological network description of predicted MBPs. The GO enriched biological network of the predicted MBPs consisted of 179 nodes and 979 edges which were structured on 17 final kappascore groups.**

| **S. No.** | **GO ID** | **GO Term** | **GO Groups** | **Nr. Genes** | **Degree** | **Associated Genes Found** |
| --- | --- | --- | --- | --- | --- | --- |
| 1 | GO:0044260 | cellular macromolecule metabolic process | [Group10, Group14] | 101 | 112 | [OTT_0021, OTT_0166, OTT_0179, OTT_0183, OTT_0256, OTT_0290, OTT_0306, OTT_0314, OTT_0453, OTT_0766, OTT_0888, OTT_1002, OTT_1033, OTT_1223, OTT_1464, OTT_1581, OTT_1698, OTT_1731, OTT_1854, alaS, aspS, codA, cspA, cysS, czcR, def, dnaG, dnaJ, dnaN, dnaQ, dnaX, fusA, gatA, gatB, gltX1, gltX2, greA, gyrB, hemK, ileS, infA, infB, lepA, leuS, ligA, lysS, metS, miaA, miaB, mnmG, mraY, murA, murB, murD, murE, murF, mutS, nrdA, nth, ntrX, pcnB, pheS, pheT, phoB, pnp, priA, proS, recA, recD, recJ, recR, rho, rluC, rnc, rne, rnhB, rph, rplB, rplK, rplP, rplX, rpmF, rpoD, rpoH, rpsD, ruvC, serS, ssb, thdF, tilS, topA, trpS, truB, tsf, tuf, uvrA, xerC, xerD, xthA2, ybeY] |
| 2 | GO:0006139 | nucleobase-containing compound metabolic process | [Group10, Group13] | 93 | 101 | [OTT_0021, OTT_0166, OTT_0179, OTT_0183, OTT_0256, OTT_0290, OTT_0306, OTT_0314, OTT_0453, OTT_0766, OTT_0888, OTT_1002, OTT_1033, OTT_1223, OTT_1464, OTT_1581, OTT_1698, OTT_1731, OTT_1854, alaS, aspS, atpA, atpD, atpG, codA, coxA, cspA, cysS, czcR, dcd, dnaG, dnaJ, dnaN, dnaQ, dnaX, dut, gltX1, gltX2, gmk, greA, gyrB, ileS, leuS, ligA, lysS, metS, miaA, miaB, mnmG, murA, mutS, ndk, nrdA, nth, ntrX, nuoG, pcnB, petB, pgk, pheS, pheT, phoB, pnp, priA, proS, purC, recA, recD, recJ, recR, rho, rluC, rnc, rne, rnhB, rph, rpoD, rpoH, ruvC, serS, ssb, thdF, tilS, tmk, topA, tpiA, trpS, truB, uvrA, xerC, xerD, xthA2, ybeY] |
| 3 | GO:0034645 | cellular macromolecule biosynthetic process | [Group10, Group13, Group14] | 67 | 87 | [OTT_0166, OTT_0183, OTT_0256, OTT_0290, OTT_0306, OTT_0314, OTT_0453, OTT_0888, OTT_1033, OTT_1464, OTT_1698, OTT_1731, OTT_1854, alaS, aspS, cspA, cysS, czcR, def, dnaG, dnaJ, dnaN, dnaQ, dnaX, fusA, gatA, gatB, gltX1, gltX2, greA, gyrB, ileS, infA, infB, lepA, leuS, ligA, lysS, metS, mraY, murA, murB, murD, murE, murF, nrdA, ntrX, pheS, pheT, phoB, priA, proS, rho, rplB, rplK, rplP, rplX, rpmF, rpoD, rpoH, rpsD, serS, ssb, trpS, tsf, tuf] |
| 4 | GO:0044271 | cellular nitrogen compound biosynthetic process | [Group13, Group14] | 69 | 87 | [OTT_0166, OTT_0256, OTT_0290, OTT_0314, OTT_0453, OTT_0888, OTT_1033, OTT_1072, OTT_1464, OTT_1698, OTT_1731, OTT_1854, alaS, aspS, atpA, atpD, atpG, cspA, cysS, czcR, dcd, def, dnaG, dnaN, dnaQ, dnaX, dut, folC, fusA, gatA, gatB, gltX1, gltX2, greA, hemB, hemH, hemN, ileS, infA, infB, lepA, leuS, lysS, metS, murA, murD, ndk, ntrX, pheS, pheT, phoB, priA, proS, purC, rho, rplB, rplK, rplP, rplX, rpmF, rpoD, rpoH, rpsD, serS, tmk, trpS, tsf, tuf] |
| 5 | GO:0090304 | nucleic acid metabolic process | [Group10, Group13] | 78 | 86 | [OTT_0021, OTT_0166, OTT_0179, OTT_0183, OTT_0256, OTT_0290, OTT_0306, OTT_0314, OTT_0453, OTT_0766, OTT_0888, OTT_1002, OTT_1033, OTT_1223, OTT_1464, OTT_1581, OTT_1698, OTT_1731, OTT_1854, alaS, aspS, codA, cspA, cysS, czcR, dnaG, dnaJ, dnaN, dnaQ, dnaX, gltX1, gltX2, greA, gyrB, ileS, leuS, ligA, lysS, metS, miaA, miaB, mnmG, mutS, nrdA, nth, ntrX, pcnB, pheS, pheT, phoB, pnp, priA, proS, recA, recD, recJ, recR, rho, rluC, rnc, rne, rnhB, rph, rpoD, rpoH, ruvC, serS, ssb, thdF, tilS, topA, trpS, truB, uvrA, xerC, xerD, xthA2, ybeY] |
| 6 | GO:0009059 | macromolecule biosynthetic process | [Group10, Group13, Group14] | 67 | 83 | [OTT_0166, OTT_0183, OTT_0256, OTT_0290, OTT_0306, OTT_0314, OTT_0453, OTT_0888, OTT_1033, OTT_1464, OTT_1698, OTT_1731, OTT_1854, alaS, aspS, cspA, cysS, czcR, def, dnaG, dnaJ, dnaN, dnaQ, dnaX, fusA, gatA, gatB, gltX1, gltX2, greA, gyrB, ileS, infA, infB, lepA, leuS, ligA, lysS, metS, mraY, murA, murB, murD, murE, murF, nrdA, ntrX, pheS, pheT, phoB, priA, proS, rho, rplB, rplK, rplP, rplX, rpmF, rpoD, rpoH, rpsD, serS, ssb, trpS, tsf, tuf] |
| 7 | GO:0010467 | gene expression | [Group10, Group14] | 56 | 73 | [OTT_1002, OTT_1581, alaS, aspS, codA, cspA, cysS, czcR, def, dksA, fusA, gatA, gatB, gltX1, gltX2, greA, hscB, ileS, infA, infB, lepA, leuS, lysS, metS, miaA, miaB, mnmG, ntrX, pcnB, pheS, pheT, phoB, pnp, proS, rho, rnc, rne, rph, rplB, rplK, rplP, rplX, rpmF, rpoD, rpoH, rpsD, serS, sufA, thdF, tilS, trpS, truB, tsf, tuf, ybeY] |
| 8 | GO:1901566 | organonitrogen compound biosynthetic process | [Group14] | 54 | 69 | [OTT_1072, alaS, argB, aspS, atpA, atpD, atpG, cysS, dapA, dapE, dcd, def, dut, folC, fusA, gatA, gatB, gltX1, gltX2, glyA, hemB, hemH, hemN, ileS, infA, infB, lepA, leuS, lysC, lysS, metS, mraY, murA, murB, murD, murE, murF, ndk, pheS, pheT, proS, purC, rplB, rplK, rplP, rplX, rpmF, rpsD, serS, tmk, trpS, tsf, tuf] |
| 9 | GO:0006753 | nucleoside phosphate metabolic process | [Group16] | 14 | 68 | [atpA, atpD, atpG, coxA, dcd, dut, gmk, ndk, nuoG, petB, pgk, purC, tmk, tpiA] |
| 10 | GO:0016070 | RNA metabolic process | [Group10, Group14, Group15] | 52 | 67 | [OTT_0166, OTT_0256, OTT_0290, OTT_0314, OTT_0453, OTT_0888, OTT_1002, OTT_1033, OTT_1464, OTT_1581, OTT_1698, OTT_1731, OTT_1854, alaS, aspS, codA, cspA, cysS, czcR, dnaG, gltX1, gltX2, greA, ileS, leuS, lysS, metS, miaA, miaB, mnmG, ntrX, pcnB, pheS, pheT, phoB, pnp, priA, proS, rho, rluC, rnc, rne, rnhB, rph, rpoD, rpoH, serS, thdF, tilS, trpS, truB, ybeY] |
| 11 | GO:0009117 | nucleotide metabolic process | [Group16] | 14 | 66 | [atpA, atpD, atpG, coxA, dcd, dut, gmk, ndk, nuoG, petB, pgk, purC, tmk, tpiA] |
| 12 | GO:0009205 | purine ribonucleoside triphosphate metabolic process | [Group12, Group16] | 9 | 62 | [atpA, atpD, atpG, coxA, ndk, nuoG, petB, pgk, tpiA] |
| 13 | GO:0055086 | nucleobase-containing small molecule metabolic process | [Group16] | 14 | 62 | [atpA, atpD, atpG, coxA, dcd, dut, gmk, ndk, nuoG, petB, pgk, purC, tmk, tpiA] |
| 14 | GO:0009144 | purine nucleoside triphosphate metabolic process | [Group12, Group16] | 9 | 61 | [atpA, atpD, atpG, coxA, ndk, nuoG, petB, pgk, tpiA] |
| 15 | GO:0009199 | ribonucleoside triphosphate metabolic process | [Group12, Group16] | 9 | 61 | [atpA, atpD, atpG, coxA, ndk, nuoG, petB, pgk, tpiA] |
| 16 | GO:0006163 | purine nucleotide metabolic process | [Group16] | 11 | 61 | [atpA, atpD, atpG, coxA, gmk, ndk, nuoG, petB, pgk, purC, tpiA] |
| 17 | GO:0009150 | purine ribonucleotide metabolic process | [Group16] | 11 | 61 | [atpA, atpD, atpG, coxA, gmk, ndk, nuoG, petB, pgk, purC, tpiA] |
| 18 | GO:0009259 | ribonucleotide metabolic process | [Group16] | 11 | 61 | [atpA, atpD, atpG, coxA, gmk, ndk, nuoG, petB, pgk, purC, tpiA] |
| 19 | GO:0019693 | ribose phosphate metabolic process | [Group16] | 11 | 61 | [atpA, atpD, atpG, coxA, gmk, ndk, nuoG, petB, pgk, purC, tpiA] |
| 20 | GO:0009167 | purine ribonucleoside monophosphate metabolic process | [Group12, Group16] | 10 | 60 | [atpA, atpD, atpG, coxA, gmk, nuoG, petB, pgk, purC, tpiA] |
| 21 | GO:1901137 | carbohydrate derivative biosynthetic process | [Group16] | 14 | 60 | [atpA, atpD, atpG, dcd, dut, mraY, murA, murB, murD, murE, murF, ndk, purC, tmk] |
| 22 | GO:0009126 | purine nucleoside monophosphate metabolic process | [Group12, Group16] | 10 | 59 | [atpA, atpD, atpG, coxA, gmk, nuoG, petB, pgk, purC, tpiA] |
| 23 | GO:0009161 | ribonucleoside monophosphate metabolic process | [Group12, Group16] | 10 | 59 | [atpA, atpD, atpG, coxA, gmk, nuoG, petB, pgk, purC, tpiA] |
| 24 | GO:0072521 | purine-containing compound metabolic process | [Group16] | 11 | 59 | [atpA, atpD, atpG, coxA, gmk, ndk, nuoG, petB, pgk, purC, tpiA] |
| 25 | GO:0009141 | nucleoside triphosphate metabolic process | [Group16] | 11 | 58 | [atpA, atpD, atpG, coxA, dcd, dut, ndk, nuoG, petB, pgk, tpiA] |
| 26 | GO:0009123 | nucleoside monophosphate metabolic process | [Group16] | 12 | 57 | [atpA, atpD, atpG, coxA, dcd, dut, gmk, nuoG, petB, pgk, purC, tpiA] |
| 27 | GO:0006259 | DNA metabolic process | [Group08, Group10, Group13] | 39 | 56 | [OTT_0021, OTT_0166, OTT_0179, OTT_0183, OTT_0256, OTT_0290, OTT_0306, OTT_0314, OTT_0453, OTT_0766, OTT_0888, OTT_1033, OTT_1223, OTT_1464, OTT_1698, OTT_1731, OTT_1854, dnaG, dnaJ, dnaN, dnaQ, dnaX, gyrB, ligA, mutS, nrdA, nth, priA, recA, recD, recJ, recR, ruvC, ssb, topA, uvrA, xerC, xerD, xthA2] |
| 28 | GO:0032774 | RNA biosynthetic process | [Group10, Group13, Group15] | 22 | 56 | [OTT_0166, OTT_0256, OTT_0290, OTT_0314, OTT_0453, OTT_0888, OTT_1033, OTT_1464, OTT_1698, OTT_1731, OTT_1854, cspA, czcR, dnaG, greA, ntrX, phoB, priA, rho, rpoD, rpoH, serS] |
| 29 | GO:0046034 | ATP metabolic process | [Group12, Group16] | 8 | 56 | [atpA, atpD, atpG, coxA, nuoG, petB, pgk, tpiA] |
| 30 | GO:0015992 | proton transport | [Group12, Group16] | 7 | 54 | [atpA, atpD, atpG, coxA, coxC, kefB, petB] |
| 31 | GO:1901293 | nucleoside phosphate biosynthetic process | [Group16] | 8 | 54 | [atpA, atpD, atpG, dcd, dut, ndk, purC, tmk] |
| 32 | GO:0015672 | monovalent inorganic cation transport | [Group12, Group16] | 7 | 53 | [atpA, atpD, atpG, coxA, coxC, kefB, petB] |
| 33 | GO:1902600 | hydrogen ion transmembrane transport | [Group12, Group16] | 7 | 53 | [atpA, atpD, atpG, coxA, coxC, kefB, petB] |
| 34 | GO:0018130 | heterocycle biosynthetic process | [Group13, Group14, Group15] | 41 | 53 | [OTT_0166, OTT_0256, OTT_0290, OTT_0314, OTT_0453, OTT_0888, OTT_1033, OTT_1072, OTT_1464, OTT_1698, OTT_1731, OTT_1854, atpA, atpD, atpG, cspA, czcR, dcd, dnaG, dnaN, dnaQ, dnaX, dut, folC, greA, hemB, hemH, hemN, lipA, murA, murD, ndk, ntrX, phoB, priA, purC, rho, rpoD, rpoH, serS, tmk] |
| 35 | GO:1901362 | organic cyclic compound biosynthetic process | [Group13, Group14, Group15] | 41 | 53 | [OTT_0166, OTT_0256, OTT_0290, OTT_0314, OTT_0453, OTT_0888, OTT_1033, OTT_1072, OTT_1464, OTT_1698, OTT_1731, OTT_1854, atpA, atpD, atpG, cspA, czcR, dcd, dnaG, dnaN, dnaQ, dnaX, dut, folC, greA, hemB, hemH, hemN, lipA, murA, murD, ndk, ntrX, phoB, priA, purC, rho, rpoD, rpoH, serS, tmk] |
| 36 | GO:0006412 | translation | [Group14] | 30 | 53 | [alaS, aspS, cysS, def, fusA, gatA, gatB, gltX1, gltX2, ileS, infA, infB, lepA, leuS, lysS, metS, pheS, pheT, proS, rplB, rplK, rplP, rplX, rpmF, rpsD, serS, trpS, tsf, tuf] |
| 37 | GO:0019538 | protein metabolic process | [Group14] | 38 | 53 | [OTT_1939, alaS, aprD, aspS, ctaQ, cysS, def, ftsH, fusA, gatA, gatB, gltX1, gltX2, hemK, hscB, ileS, infA, infB, lepA, leuS, lysS, metS, pheS, pheT, proS, rplB, rplK, rplP, rplX, rpmF, rpsD, serS, sufA, trpS, tsf, tuf, ybeY] |
| 38 | GO:0009165 | nucleotide biosynthetic process | [Group16] | 8 | 53 | [atpA, atpD, atpG, dcd, dut, ndk, purC, tmk] |
| 39 | GO:0019637 | organophosphate metabolic process | [Group16] | 15 | 53 | [atpA, atpD, atpG, cdsA, coxA, dcd, dut, gmk, ndk, nuoG, petB, pgk, purC, tmk, tpiA] |
| 40 | GO:0072522 | purine-containing compound biosynthetic process | [Group16] | 5 | 53 | [atpA, atpD, atpG, ndk, purC] |
| 41 | GO:0006818 | hydrogen transport | [Group12, Group16] | 7 | 52 | [atpA, atpD, atpG, coxA, coxC, kefB, petB] |
| 42 | GO:0098662 | inorganic cation transmembrane transport | [Group12, Group16] | 9 | 51 | [atpA, atpD, atpG, coxA, coxC, kefB, mgtE, petB, znuC] |
| 43 | GO:0019438 | aromatic compound biosynthetic process | [Group13, Group14, Group15] | 40 | 51 | [OTT_0166, OTT_0256, OTT_0290, OTT_0314, OTT_0453, OTT_0888, OTT_1033, OTT_1072, OTT_1464, OTT_1698, OTT_1731, OTT_1854, atpA, atpD, atpG, cspA, czcR, dcd, dnaG, dnaN, dnaQ, dnaX, dut, folC, greA, hemB, hemH, hemN, murA, murD, ndk, ntrX, phoB, priA, purC, rho, rpoD, rpoH, serS, tmk] |
| 45 | GO:0043043 | peptide biosynthetic process | [Group14] | 30 | 51 | [alaS, aspS, cysS, def, fusA, gatA, gatB, gltX1, gltX2, ileS, infA, infB, lepA, leuS, lysS, metS, pheS, pheT, proS, rplB, rplK, rplP, rplX, rpmF, rpsD, serS, trpS, tsf, tuf] |
| 45 | GO:0043604 | amide biosynthetic process | [Group14] | 32 | 51 | [alaS, aspS, cysS, def, folC, fusA, gatA, gatB, gltX1, gltX2, ileS, infA, infB, lepA, leuS, lysS, metS, murD, pheS, pheT, proS, rplB, rplK, rplP, rplX, rpmF, rpsD, serS, trpS, tsf, tuf] |
| 46 | GO:0044267 | cellular protein metabolic process | [Group14] | 31 | 51 | [alaS, aspS, cysS, def, fusA, gatA, gatB, gltX1, gltX2, hemK, ileS, infA, infB, lepA, leuS, lysS, metS, pheS, pheT, proS, rplB, rplK, rplP, rplX, rpmF, rpsD, serS, trpS, tsf, tuf] |
| 47 | GO:0009142 | nucleoside triphosphate biosynthetic process | [Group16] | 5 | 51 | [atpA, atpD, atpG, dcd, ndk] |
| 48 | GO:0043603 | cellular amide metabolic process | [Group14] | 33 | 50 | [alaS, aspS, cysS, def, folC, fusA, gatA, gatB, gltX1, gltX2, glyA, ileS, infA, infB, lepA, leuS, lysS, metS, murD, pheS, pheT, proS, rplB, rplK, rplP, rplX, rpmF, rpsD, serS, trpS, tsf, tuf] |
| 49 | GO:0006164 | purine nucleotide biosynthetic process | [Group16] | 5 | 50 | [atpA, atpD, atpG, ndk, purC] |
| 50 | GO:0009260 | ribonucleotide biosynthetic process | [Group16] | 5 | 50 | [atpA, atpD, atpG, ndk, purC] |
| 51 | GO:0046390 | ribose phosphate biosynthetic process | [Group16] | 5 | 50 | [atpA, atpD, atpG, ndk, purC] |
| 52 | GO:0034654 | nucleobase-containing compound biosynthetic process | [Group13, Group14, Group15] | 34 | 49 | [OTT_0166, OTT_0256, OTT_0290, OTT_0314, OTT_0453, OTT_0888, OTT_1033, OTT_1464, OTT_1698, OTT_1731, OTT_1854, atpA, atpD, atpG, cspA, czcR, dcd, dnaG, dnaN, dnaQ, dnaX, dut, greA, murA, ndk, ntrX, phoB, priA, purC, rho, rpoD, rpoH, serS, tmk] |
| 53 | GO:0006518 | peptide metabolic process | [Group14] | 30 | 49 | [alaS, aspS, cysS, def, fusA, gatA, gatB, gltX1, gltX2, ileS, infA, infB, lepA, leuS, lysS, metS, pheS, pheT, proS, rplB, rplK, rplP, rplX, rpmF, rpsD, serS, trpS, tsf, tuf] |
| 54 | GO:0009127 | purine nucleoside monophosphate biosynthetic process | [Group16] | 4 | 49 | [atpA, atpD, atpG, purC] |
| 55 | GO:0009145 | purine nucleoside triphosphate biosynthetic process | [Group16] | 4 | 49 | [atpA, atpD, atpG, ndk] |
| 56 | GO:0009152 | purine ribonucleotide biosynthetic process | [Group16] | 5 | 49 | [atpA, atpD, atpG, ndk, purC] |
| 57 | GO:0009156 | ribonucleoside monophosphate biosynthetic process | [Group16] | 4 | 49 | [atpA, atpD, atpG, purC] |
| 58 | GO:0009168 | purine ribonucleoside monophosphate biosynthetic process | [Group16] | 4 | 49 | [atpA, atpD, atpG, purC] |
| 59 | GO:0009201 | ribonucleoside triphosphate biosynthetic process | [Group16] | 4 | 49 | [atpA, atpD, atpG, ndk] |
| 60 | GO:0009206 | purine ribonucleoside triphosphate biosynthetic process | [Group16] | 4 | 49 | [atpA, atpD, atpG, ndk] |
| 61 | GO:0090407 | organophosphate biosynthetic process | [Group16] | 9 | 49 | [atpA, atpD, atpG, cdsA, dcd, dut, ndk, purC, tmk] |
| 62 | GO:0019752 | carboxylic acid metabolic process | [Group07, Group11, Group14] | 35 | 48 | [acpP, acpS, alaS, argB, aspS, cysS, dapA, dapE, fabF, folC, fumC, gltX1, gltX2, glyA, icd, ileS, leuS, lipA, lysC, lysS, mdh, metS, murD, pgk, pheS, pheT, proS, sdhB, serS, sucA, sucB, sucD, tdcB, tpiA, trpS] |
| 63 | GO:0009124 | nucleoside monophosphate biosynthetic process | [Group16] | 6 | 48 | [atpA, atpD, atpG, dcd, dut, purC] |
| 64 | GO:0006812 | cation transport | [Group12, Group16] | 10 | 46 | [OTT_1942, atpA, atpD, atpG, coxA, coxC, kefB, mgtE, petB, znuC] |
| 65 | GO:0098655 | cation transmembrane transport | [Group12, Group16] | 10 | 46 | [OTT_1942, atpA, atpD, atpG, coxA, coxC, kefB, mgtE, petB, znuC] |
| 66 | GO:0098660 | inorganic ion transmembrane transport | [Group12, Group16] | 10 | 46 | [atpA, atpD, atpG, coxA, coxC, glnQ, kefB, mgtE, petB, znuC] |
| 67 | GO:0006796 | phosphate-containing compound metabolic process | [Group16] | 19 | 46 | [argB, atpA, atpD, atpG, cdsA, coxA, dcd, dut, gmk, lysC, ndk, nuoG, petB, pgk, pkcI, ppa, purC, tmk, tpiA] |
| 68 | GO:0006399 | tRNA metabolic process | [Group06, Group10, Group14] | 25 | 44 | [OTT_1002, OTT_1581, alaS, aspS, codA, cysS, gltX1, gltX2, ileS, leuS, lysS, metS, miaA, miaB, mnmG, pheS, pheT, proS, rnc, rph, serS, thdF, tilS, trpS, truB] |
| 69 | GO:0034660 | ncRNA metabolic process | [Group06, Group10, Group14] | 26 | 43 | [OTT_1002, OTT_1581, alaS, aspS, codA, cysS, gltX1, gltX2, ileS, leuS, lysS, metS, miaA, miaB, mnmG, pheS, pheT, proS, rnc, rph, serS, thdF, tilS, trpS, truB, ybeY] |
| 70 | GO:0043436 | oxoacid metabolic process | [Group07, Group11, Group14] | 35 | 43 | [acpP, acpS, alaS, argB, aspS, cysS, dapA, dapE, fabF, folC, fumC, gltX1, gltX2, glyA, icd, ileS, leuS, lipA, lysC, lysS, mdh, metS, murD, pgk, pheS, pheT, proS, sdhB, serS, sucA, sucB, sucD, tdcB, tpiA, trpS] |
| 71 | GO:0006520 | cellular amino acid metabolic process | [Group07, Group09, Group14] | 21 | 40 | [alaS, argB, aspS, cysS, dapA, dapE, gltX1, gltX2, glyA, ileS, leuS, lysC, lysS, metS, pheS, pheT, proS, serS, sucB, tdcB, trpS] |
| 72 | GO:0006260 | DNA replication | [Group13, Group14, Group15] | 23 | 40 | [OTT_0166, OTT_0183, OTT_0256, OTT_0290, OTT_0306, OTT_0314, OTT_0453, OTT_0888, OTT_1033, OTT_1464, OTT_1698, OTT_1731, OTT_1854, dnaG, dnaJ, dnaN, dnaQ, dnaX, gyrB, ligA, nrdA, priA, ssb] |
| 73 | GO:0034220 | ion transmembrane transport | [Group16] | 11 | 40 | [OTT_1942, atpA, atpD, atpG, coxA, coxC, glnQ, kefB, mgtE, petB, znuC] |
| 74 | GO:0090662 | ATP hydrolysis coupled transmembrane transport | [Group16] | 4 | 40 | [atpA, atpD, atpG, glnQ] |
| 75 | GO:0099131 | ATP hydrolysis coupled ion transmembrane transport | [Group16] | 4 | 40 | [atpA, atpD, atpG, glnQ] |
| 76 | GO:0006811 | ion transport | [Group16] | 11 | 38 | [OTT_1942, atpA, atpD, atpG, coxA, coxC, glnQ, kefB, mgtE, petB, znuC] |
| 77 | GO:0016310 | phosphorylation | [Group16] | 11 | 34 | [argB, coxA, gmk, lysC, ndk, nuoG, petB, pgk, pkcI, tmk, tpiA] |
| 78 | GO:0010556 | regulation of macromolecule biosynthetic process | [Group15] | 10 | 33 | [cspA, czcR, greA, ileS, leuS, ntrX, phoB, rho, rpoD, rpoH] |
| 79 | GO:0019219 | regulation of nucleobase-containing compound metabolic process | [Group15] | 8 | 32 | [cspA, czcR, greA, ntrX, phoB, rho, rpoD, rpoH] |
| 80 | GO:0031326 | regulation of cellular biosynthetic process | [Group15] | 10 | 32 | [cspA, czcR, greA, ileS, leuS, ntrX, phoB, rho, rpoD, rpoH] |
| 81 | GO:2000112 | regulation of cellular macromolecule biosynthetic process | [Group15] | 10 | 32 | [cspA, czcR, greA, ileS, leuS, ntrX, phoB, rho, rpoD, rpoH] |
| 82 | GO:2001141 | regulation of RNA biosynthetic process | [Group15] | 8 | 32 | [cspA, czcR, greA, ntrX, phoB, rho, rpoD, rpoH] |
| 83 | GO:0043039 | tRNA aminoacylation | [Group07, Group14] | 14 | 31 | [alaS, aspS, cysS, gltX1, gltX2, ileS, leuS, lysS, metS, pheS, pheT, proS, serS, trpS] |
| 84 | GO:0006351 | transcription, DNA-templated | [Group15] | 8 | 31 | [cspA, czcR, greA, ntrX, phoB, rho, rpoD, rpoH] |
| 85 | GO:0006355 | regulation of transcription, DNA-templated | [Group15] | 8 | 31 | [cspA, czcR, greA, ntrX, phoB, rho, rpoD, rpoH] |
| 86 | GO:0010468 | regulation of gene expression | [Group15] | 12 | 31 | [cspA, czcR, dksA, greA, ileS, leuS, ntrX, phoB, pnp, rho, rpoD, rpoH] |
| 87 | GO:0051252 | regulation of RNA metabolic process | [Group15] | 8 | 31 | [cspA, czcR, greA, ntrX, phoB, rho, rpoD, rpoH] |
| 88 | GO:0060255 | regulation of macromolecule metabolic process | [Group15] | 12 | 31 | [cspA, czcR, dksA, greA, ileS, leuS, ntrX, phoB, pnp, rho, rpoD, rpoH] |
| 89 | GO:0006418 | tRNA aminoacylation for protein translation | [Group07, Group14] | 14 | 30 | [alaS, aspS, cysS, gltX1, gltX2, ileS, leuS, lysS, metS, pheS, pheT, proS, serS, trpS] |
| 90 | GO:0043038 | amino acid activation | [Group07, Group14] | 14 | 30 | [alaS, aspS, cysS, gltX1, gltX2, ileS, leuS, lysS, metS, pheS, pheT, proS, serS, trpS] |
| 91 | GO:0071103 | DNA conformation change | [Group13, Group15] | 20 | 30 | [OTT_0021, OTT_0166, OTT_0183, OTT_0256, OTT_0290, OTT_0306, OTT_0314, OTT_0453, OTT_0888, OTT_1033, OTT_1464, OTT_1698, OTT_1731, OTT_1854, gyrB, hupA, priA, recD, topA, uvrD] |
| 92 | GO:0009889 | regulation of biosynthetic process | [Group15] | 10 | 30 | [cspA, czcR, greA, ileS, leuS, ntrX, phoB, rho, rpoD, rpoH] |
| 93 | GO:0031323 | regulation of cellular metabolic process | [Group15] | 10 | 30 | [cspA, czcR, greA, ileS, leuS, ntrX, phoB, rho, rpoD, rpoH] |
| 94 | GO:0097659 | nucleic acid-templated transcription | [Group15] | 8 | 30 | [cspA, czcR, greA, ntrX, phoB, rho, rpoD, rpoH] |
| 95 | GO:1903506 | regulation of nucleic acid-templated transcription | [Group15] | 8 | 30 | [cspA, czcR, greA, ntrX, phoB, rho, rpoD, rpoH] |
| 96 | GO:0051276 | chromosome organization | [Group13, Group15] | 20 | 29 | [OTT_0021, OTT_0166, OTT_0183, OTT_0256, OTT_0290, OTT_0306, OTT_0314, OTT_0453, OTT_0888, OTT_1033, OTT_1464, OTT_1698, OTT_1731, OTT_1854, gyrB, hupA, priA, recD, topA, uvrD] |
| 97 | GO:0051171 | regulation of nitrogen compound metabolic process | [Group15] | 10 | 29 | [cspA, czcR, greA, ileS, leuS, ntrX, phoB, rho, rpoD, rpoH] |
| 98 | GO:0080090 | regulation of primary metabolic process | [Group15] | 10 | 29 | [cspA, czcR, greA, ileS, leuS, ntrX, phoB, rho, rpoD, rpoH] |
| 99 | GO:0055085 | transmembrane transport | [Group16] | 17 | 29 | [OTT_0530, OTT_1942, abcT3, aprD, atpA, atpD, atpG, ccmA, coxA, coxC, glnQ, kefB, mgtE, msbA1, msbA2, petB, znuC] |
| 100 | GO:0046394 | carboxylic acid biosynthetic process | [Group07, Group09, Group11] | 11 | 28 | [acpP, acpS, argB, dapA, dapE, fabF, folC, glyA, lipA, lysC, murD] |
| 101 | GO:0006261 | DNA-dependent DNA replication | [Group13, Group15] | 14 | 28 | [OTT_0166, OTT_0256, OTT_0290, OTT_0314, OTT_0453, OTT_0888, OTT_1033, OTT_1464, OTT_1698, OTT_1731, OTT_1854, dnaG, gyrB, priA] |
| 102 | GO:0006269 | DNA replication, synthesis of RNA primer | [Group13, Group15] | 13 | 28 | [OTT_0166, OTT_0256, OTT_0290, OTT_0314, OTT_0453, OTT_0888, OTT_1033, OTT_1464, OTT_1698, OTT_1731, OTT_1854, dnaG, priA] |
| 103 | GO:0006023 | aminoglycan biosynthetic process | [Group16] | 6 | 28 | [mraY, murA, murB, murD, murE, murF] |
| 104 | GO:0032392 | DNA geometric change | [Group13, Group15] | 17 | 27 | [OTT_0021, OTT_0166, OTT_0183, OTT_0256, OTT_0290, OTT_0306, OTT_0314, OTT_0453, OTT_0888, OTT_1033, OTT_1464, OTT_1698, OTT_1731, OTT_1854, priA, recD, uvrD] |
| 105 | GO:0009252 | peptidoglycan biosynthetic process | [Group16] | 6 | 27 | [mraY, murA, murB, murD, murE, murF] |
| 106 | GO:0022604 | regulation of cell morphogenesis | [Group16] | 6 | 27 | [mraY, murA, murB, murD, murE, murF] |
| 107 | GO:0030203 | glycosaminoglycan metabolic process | [Group16] | 7 | 27 | [OTT_1510, mraY, murA, murB, murD, murE, murF] |
| 108 | GO:0032508 | DNA duplex unwinding | [Group13, Group15] | 17 | 26 | [OTT_0021, OTT_0166, OTT_0183, OTT_0256, OTT_0290, OTT_0306, OTT_0314, OTT_0453, OTT_0888, OTT_1033, OTT_1464, OTT_1698, OTT_1731, OTT_1854, priA, recD, uvrD] |
| 109 | GO:0000270 | peptidoglycan metabolic process | [Group16] | 7 | 26 | [OTT_1510, mraY, murA, murB, murD, murE, murF] |
| 110 | GO:0000902 | cell morphogenesis | [Group16] | 7 | 26 | [mraY, mreB, murA, murB, murD, murE, murF] |
| 111 | GO:0006022 | aminoglycan metabolic process | [Group16] | 7 | 26 | [OTT_1510, mraY, murA, murB, murD, murE, murF] |
| 112 | GO:0006024 | glycosaminoglycan biosynthetic process | [Group16] | 6 | 26 | [mraY, murA, murB, murD, murE, murF] |
| 113 | GO:0044038 | cell wall macromolecule biosynthetic process | [Group16] | 6 | 26 | [mraY, murA, murB, murD, murE, murF] |
| 114 | GO:0006396 | RNA processing | [Group06, Group14] | 15 | 25 | [OTT_1002, OTT_1581, codA, miaA, miaB, mnmG, pcnB, pnp, rnc, rne, rph, thdF, tilS, truB, ybeY] |
| 115 | GO:0016053 | organic acid biosynthetic process | [Group07, Group09, Group11] | 11 | 25 | [acpP, acpS, argB, dapA, dapE, fabF, folC, glyA, lipA, lysC, murD] |
| 116 | GO:0032989 | cellular component morphogenesis | [Group16] | 7 | 25 | [mraY, mreB, murA, murB, murD, murE, murF] |
| 117 | GO:0044036 | cell wall macromolecule metabolic process | [Group16] | 6 | 25 | [mraY, murA, murB, murD, murE, murF] |
| 118 | GO:0070589 | cellular component macromolecule biosynthetic process | [Group16] | 6 | 25 | [mraY, murA, murB, murD, murE, murF] |
| 119 | GO:0008360 | regulation of cell shape | [Group16] | 6 | 24 | [mraY, murA, murB, murD, murE, murF] |
| 120 | GO:0009132 | nucleoside diphosphate metabolic process | [Group16] | 5 | 24 | [gmk, ndk, pgk, tmk, tpiA] |
| 121 | GO:0009273 | peptidoglycan-based cell wall biogenesis | [Group16] | 6 | 24 | [mraY, murA, murB, murD, murE, murF] |
| 122 | GO:0022603 | regulation of anatomical structure morphogenesis | [Group16] | 6 | 24 | [mraY, murA, murB, murD, murE, murF] |
| 123 | GO:0051128 | regulation of cellular component organization | [Group16] | 6 | 24 | [mraY, murA, murB, murD, murE, murF] |
| 124 | GO:0071555 | cell wall organization | [Group16] | 6 | 23 | [mraY, murA, murB, murD, murE, murF] |
| 125 | GO:0072528 | pyrimidine-containing compound biosynthetic process | [Group16] | 4 | 23 | [dcd, dut, ndk, tmk] |
| 126 | GO:0034470 | ncRNA processing | [Group06, Group14] | 12 | 22 | [OTT_1002, OTT_1581, codA, miaA, miaB, mnmG, rnc, rph, thdF, tilS, truB, ybeY] |
| 127 | GO:0035556 | intracellular signal transduction | [Group15] | 4 | 22 | [OTT_1084, czcR, ntrX, phoB] |
| 128 | GO:0022900 | electron transport chain | [Group05, Group12, Group16] | 4 | 21 | [coxA, coxC, nuoG, petB] |
| 129 | GO:0008033 | tRNA processing | [Group06, Group14] | 11 | 21 | [OTT_1002, OTT_1581, codA, miaA, miaB, mnmG, rnc, rph, thdF, tilS, truB] |
| 130 | GO:0000160 | phosphorelay signal transduction system | [Group15] | 4 | 21 | [OTT_1084, czcR, ntrX, phoB] |
| 131 | GO:0006281 | DNA repair | [Group08, Group13] | 14 | 20 | [OTT_0021, OTT_0179, OTT_1223, ligA, mutS, nth, recA, recD, recJ, recR, ruvC, ssb, uvrA, xthA2] |
| 132 | GO:0006220 | pyrimidine nucleotide metabolic process | [Group16] | 4 | 20 | [dcd, dut, ndk, tmk] |
| 133 | GO:0006221 | pyrimidine nucleotide biosynthetic process | [Group16] | 4 | 20 | [dcd, dut, ndk, tmk] |
| 134 | GO:0045333 | cellular respiration | [Group05, Group12] | 11 | 19 | [coxA, coxC, fumC, icd, mdh, nuoG, petB, sdhB, sucA, sucB, sucD] |
| 135 | GO:0006974 | cellular response to DNA damage stimulus | [Group08, Group13] | 14 | 19 | [OTT_0021, OTT_0179, OTT_1223, ligA, mutS, nth, recA, recD, recJ, recR, ruvC, ssb, uvrA, xthA2] |
| 136 | GO:0090305 | nucleic acid phosphodiester bond hydrolysis | [Group08] | 14 | 19 | [OTT_0021, OTT_0179, OTT_1223, dnaN, nth, pnp, recJ, rnc, rne, rnhB, ruvC, uvrA, xthA2, ybeY] |
| 137 | GO:0072527 | pyrimidine-containing compound metabolic process | [Group16] | 4 | 19 | [dcd, dut, ndk, tmk] |
| 138 | GO:0015980 | energy derivation by oxidation of organic compounds | [Group05, Group12] | 11 | 18 | [coxA, coxC, fumC, icd, mdh, nuoG, petB, sdhB, sucA, sucB, sucD] |
| 139 | GO:0032787 | monocarboxylic acid metabolic process | [Group11] | 7 | 18 | [acpP, acpS, fabF, icd, lipA, pgk, tpiA] |
| 140 | GO:0046939 | nucleotide phosphorylation | [Group16] | 4 | 18 | [ndk, pgk, tmk, tpiA] |
| 141 | GO:1901605 | alpha-amino acid metabolic process | [Group09, Group11, Group14] | 6 | 17 | [argB, dapA, dapE, glyA, lysC, sucB] |
| 142 | GO:0033014 | tetrapyrrole biosynthetic process | [Group03] | 5 | 16 | [OTT_1072, hemB, hemH, hemN, purC] |
| 143 | GO:0009060 | aerobic respiration | [Group05] | 9 | 16 | [coxA, coxC, fumC, icd, mdh, sdhB, sucA, sucB, sucD] |
| 144 | GO:0009451 | RNA modification | [Group06] | 8 | 16 | [OTT_1002, OTT_1581, codA, miaB, mnmG, rluC, thdF, truB] |
| 145 | GO:0008652 | cellular amino acid biosynthetic process | [Group09, Group11] | 5 | 16 | [argB, dapA, dapE, glyA, lysC] |
| 146 | GO:0051188 | cofactor biosynthetic process | [Group03] | 10 | 15 | [OTT_1072, folC, hemB, hemH, hemN, iscU, lipA, murD, spl1] |
| 147 | GO:0006400 | tRNA modification | [Group06, Group14] | 7 | 15 | [OTT_1002, OTT_1581, codA, miaB, mnmG, thdF, truB] |
| 148 | GO:0043412 | macromolecule modification | [Group06] | 9 | 15 | [OTT_1002, OTT_1581, codA, hemK, miaB, mnmG, rluC, thdF, truB] |
| 149 | GO:0007165 | signal transduction | [Group15] | 5 | 15 | [OTT_1084, czcR, ntrX, phoB, yqiX] |
| 150 | GO:0006099 | tricarboxylic acid cycle | [Group05] | 7 | 14 | [fumC, icd, mdh, sdhB, sucA, sucB, sucD] |
| 151 | GO:0006101 | citrate metabolic process | [Group05] | 7 | 14 | [fumC, icd, mdh, sdhB, sucA, sucB, sucD] |
| 152 | GO:0072350 | tricarboxylic acid metabolic process | [Group05] | 7 | 14 | [fumC, icd, mdh, sdhB, sucA, sucB, sucD] |
| 153 | GO:1901607 | alpha-amino acid biosynthetic process | [Group09, Group11] | 5 | 14 | [argB, dapA, dapE, glyA, lysC] |
| 154 | GO:0006631 | fatty acid metabolic process | [Group11] | 4 | 14 | [acpP, acpS, fabF, lipA] |
| 155 | GO:0006633 | fatty acid biosynthetic process | [Group11] | 4 | 14 | [acpP, acpS, fabF, lipA] |
| 156 | GO:0008610 | lipid biosynthetic process | [Group11] | 6 | 14 | [acpP, acpS, cdsA, fabF, ispB, lipA] |
| 157 | GO:0044255 | cellular lipid metabolic process | [Group11] | 6 | 14 | [acpP, acpS, cdsA, fabF, ispB, lipA] |
| 158 | GO:0072330 | monocarboxylic acid biosynthetic process | [Group11] | 4 | 14 | [acpP, acpS, fabF, lipA] |
| 159 | GO:0015833 | peptide transport | [Group04] | 5 | 13 | [aprD, ffh, tolB, tolQ, tppB] |
| 160 | GO:0071702 | organic substance transport | [Group04] | 7 | 13 | [aprD, ccmA, ffh, glnQ, tolB, tolQ, tppB] |
| 161 | GO:0071705 | nitrogen compound transport | [Group04] | 7 | 13 | [aprD, ccmA, ffh, glnQ, tolB, tolQ, tppB] |
| 162 | GO:0042886 | amide transport | [Group04] | 5 | 12 | [aprD, ffh, tolB, tolQ, tppB] |
| 163 | GO:0006779 | porphyrin-containing compound biosynthetic process | [Group03] | 4 | 11 | [OTT_1072, hemB, hemH, hemN] |
| 164 | GO:0033013 | tetrapyrrole metabolic process | [Group03] | 5 | 11 | [OTT_1072, hemB, hemH, hemN, purC] |
| 165 | GO:0015031 | protein transport | [Group04] | 4 | 11 | [aprD, ffh, tolB, tolQ] |
| 166 | GO:0009066 | aspartate family amino acid metabolic process | [Group09] | 4 | 11 | [dapA, dapE, lysC, sucB] |
| 167 | GO:0006778 | porphyrin-containing compound metabolic process | [Group03] | 4 | 10 | [OTT_1072, hemB, hemH, hemN] |
| 168 | GO:0045184 | establishment of protein localization | [Group04] | 4 | 10 | [aprD, ffh, tolB, tolQ] |
| 169 | GO:0006310 | DNA recombination | [Group08] | 7 | 10 | [recA, recJ, recR, ruvC, ssb, xerC, xerD] |
| 170 | GO:0090501 | RNA phosphodiester bond hydrolysis | [Group08] | 5 | 10 | [pnp, rnc, rne, rnhB, ybeY] |
| 171 | GO:0006553 | lysine metabolic process | [Group09] | 4 | 10 | [dapA, dapE, lysC, sucB] |
| 172 | GO:0043648 | dicarboxylic acid metabolic process | [Group09] | 4 | 10 | [dapA, dapE, fumC, lysC] |
| 173 | GO:0006732 | coenzyme metabolic process | [Group11] | 7 | 10 | [folC, glyA, lipA, murD, pgk, sucB, tpiA] |
| 174 | GO:0006414 | translational elongation | [Group00] | 6 | 8 | [fusA, ileS, leuS, tsf, tuf] |
| 175 | GO:0019725 | cellular homeostasis | [Group02] | 5 | 7 | [grlA, grxC, sco2, tdpX1, trxA] |
| 176 | GO:0045454 | cell redox homeostasis | [Group02] | 5 | 7 | [grlA, grxC, sco2, tdpX1, trxA] |
| 177 | GO:0006508 | proteolysis | [Group01] | 5 | 6 | [OTT_1939, aprD, ctaQ, ftsH, ybeY] |
| 178 | GO:0009057 | macromolecule catabolic process | [Group08] | 4 | 5 | [OTT_1510, ftsH, pnp, rnc] |
| 179 | GO:0042254 | ribosome biogenesis | [Group08] | 4 | 5 | [der, era, rnc, ybeY] |

**Supplemetary Table S3C. GO enriched molecular network description of predicted MBPs. GO enriched molecular network of predicted MBPs had 67 nodes and 101 edges with 22 kappascore groups.**

| **S. No.** | **GO ID** | **GOTerm** | **GO Groups** | **Nr. Genes** | **Degree** | **Associated Genes Found** |
| --- | --- | --- | --- | --- | --- | --- |
| 1 | GO:0000166 | nucleotide binding | [Group20] | 98 | 111 | [OTT_0021, OTT_0166, OTT_0183, OTT_0256, OTT_0290, OTT_0306, OTT_0314, OTT_0453, OTT_0530, OTT_0625, OTT_0682, OTT_0888, OTT_1033, OTT_1464, OTT_1698, OTT_1731, OTT_1854, abcT1, abcT3, alaS, aprD, aspS, atpA, atpD, ccmA, clpX, cysS, der, dnaJ, dnaK, dnaX, engB, era, ffh, folC, ftsH, ftsZ, fusA, gatA, gatB, glnQ, gltX1, gltX2, gmk, groL, groS, gyrB, hscA, htpG, ileS, infB, lepA, leuS, lysS, metS, miaA, mkl, mnmG, mrp, msbA1, msbA2, murB, murD, murE, murF, mutS, ndk, nrdA, ntrX, nuoD, nuoF, obg, pcnB, pgk, pheS, pheT, priA, proS, purC, recA, recD, rho, serS, sucD, thdF, tilS, tlyC, tme, tmk, trpS, tuf, uup, uvrA, uvrD, virB11, ychF, znuC] |
| 2 | GO:0017076 | purine nucleotide binding | [Group20] | 90 | 104 | [OTT_0021, OTT_0166, OTT_0183, OTT_0256, OTT_0290, OTT_0306, OTT_0314, OTT_0453, OTT_0530, OTT_0682, OTT_0888, OTT_1033, OTT_1464, OTT_1698, OTT_1731, OTT_1854, abcT1, abcT3, alaS, aprD, aspS, atpA, atpD, ccmA, clpX, cysS, der, dnaJ, dnaK, dnaX, engB, era, ffh, folC, ftsH, ftsZ, fusA, gatA, gatB, glnQ, gltX1, gltX2, gmk, groL, groS, gyrB, hscA, htpG, ileS, infB, lepA, leuS, lysS, metS, miaA, mkl, mrp, msbA1, msbA2, murD, murE, murF, mutS, ndk, nrdA, ntrX, obg, pcnB, pgk, pheS, pheT, priA, proS, purC, recA, recD, rho, serS, thdF, tilS, tmk, trpS, tuf, uup, uvrA, uvrD, virB11, ychF, znuC] |
| 3 | GO:0032555 | purine ribonucleotide binding | [Group20] | 90 | 104 | [OTT_0021, OTT_0166, OTT_0183, OTT_0256, OTT_0290, OTT_0306, OTT_0314, OTT_0453, OTT_0530, OTT_0682, OTT_0888, OTT_1033, OTT_1464, OTT_1698, OTT_1731, OTT_1854, abcT1, abcT3, alaS, aprD, aspS, atpA, atpD, ccmA, clpX, cysS, der, dnaJ, dnaK, dnaX, engB, era, ffh, folC, ftsH, ftsZ, fusA, gatA, gatB, glnQ, gltX1, gltX2, gmk, groL, groS, gyrB, hscA, htpG, ileS, infB, lepA, leuS, lysS, metS, miaA, mkl, mrp, msbA1, msbA2, murD, murE, murF, mutS, ndk, nrdA, ntrX, obg, pcnB, pgk, pheS, pheT, priA, proS, purC, recA, recD, rho, serS, thdF, tilS, tmk, trpS, tuf, uup, uvrA, uvrD, virB11, ychF, znuC] |
| 4 | GO:0032553 | ribonucleotide binding | [Group20] | 91 | 103 | [OTT_0021, OTT_0166, OTT_0183, OTT_0256, OTT_0290, OTT_0306, OTT_0314, OTT_0453, OTT_0530, OTT_0682, OTT_0888, OTT_1033, OTT_1464, OTT_1698, OTT_1731, OTT_1854, abcT1, abcT3, alaS, aprD, aspS, atpA, atpD, ccmA, clpX, cysS, der, dnaJ, dnaK, dnaX, engB, era, ffh, folC, ftsH, ftsZ, fusA, gatA, gatB, glnQ, gltX1, gltX2, gmk, groL, groS, gyrB, hscA, htpG, ileS, infB, lepA, leuS, lysS, metS, miaA, mkl, mrp, msbA1, msbA2, murD, murE, murF, mutS, ndk, nrdA, ntrX, nuoF, obg, pcnB, pgk, pheS, pheT, priA, proS, purC, recA, recD, rho, serS, thdF, tilS, tmk, trpS, tuf, uup, uvrA, uvrD, virB11, ychF, znuC] |
| 5 | GO:0035639 | purine ribonucleoside triphosphate binding | [Group20] | 90 | 102 | [OTT_0021, OTT_0166, OTT_0183, OTT_0256, OTT_0290, OTT_0306, OTT_0314, OTT_0453, OTT_0530, OTT_0682, OTT_0888, OTT_1033, OTT_1464, OTT_1698, OTT_1731, OTT_1854, abcT1, abcT3, alaS, aprD, aspS, atpA, atpD, ccmA, clpX, cysS, der, dnaJ, dnaK, dnaX, engB, era, ffh, folC, ftsH, ftsZ, fusA, gatA, gatB, glnQ, gltX1, gltX2, gmk, groL, groS, gyrB, hscA, htpG, ileS, infB, lepA, leuS, lysS, metS, miaA, mkl, mrp, msbA1, msbA2, murD, murE, murF, mutS, ndk, nrdA, ntrX, obg, pcnB, pgk, pheS, pheT, priA, proS, purC, recA, recD, rho, serS, thdF, tilS, tmk, trpS, tuf, uup, uvrA, uvrD, virB11, ychF, znuC] |
| 6 | GO:0032559 | adenyl ribonucleotide binding | [Group20] | 78 | 89 | [OTT_0021, OTT_0166, OTT_0183, OTT_0256, OTT_0290, OTT_0306, OTT_0314, OTT_0453, OTT_0530, OTT_0682, OTT_0888, OTT_1033, OTT_1464, OTT_1698, OTT_1731, OTT_1854, abcT1, abcT3, alaS, aprD, aspS, atpA, atpD, ccmA, clpX, cysS, dnaJ, dnaK, dnaX, folC, ftsH, gatA, gatB, glnQ, gltX1, gltX2, gmk, groL, groS, gyrB, hscA, htpG, ileS, leuS, lysS, metS, miaA, mkl, mrp, msbA1, msbA2, murD, murE, murF, mutS, ndk, nrdA, ntrX, pcnB, pgk, pheS, pheT, priA, proS, purC, recA, recD, rho, serS, tilS, tmk, trpS, uup, uvrA, uvrD, virB11, ychF, znuC] |
| 7 | GO:0005524 | ATP binding | [Group20] | 78 | 88 | [OTT_0021, OTT_0166, OTT_0183, OTT_0256, OTT_0290, OTT_0306, OTT_0314, OTT_0453, OTT_0530, OTT_0682, OTT_0888, OTT_1033, OTT_1464, OTT_1698, OTT_1731, OTT_1854, abcT1, abcT3, alaS, aprD, aspS, atpA, atpD, ccmA, clpX, cysS, dnaJ, dnaK, dnaX, folC, ftsH, gatA, gatB, glnQ, gltX1, gltX2, gmk, groL, groS, gyrB, hscA, htpG, ileS, leuS, lysS, metS, miaA, mkl, mrp, msbA1, msbA2, murD, murE, murF, mutS, ndk, nrdA, ntrX, pcnB, pgk, pheS, pheT, priA, proS, purC, recA, recD, rho, serS, tilS, tmk, trpS, uup, uvrA, uvrD, virB11, ychF, znuC] |
| 8 | GO:0030554 | adenyl nucleotide binding | [Group20] | 78 | 88 | [OTT_0021, OTT_0166, OTT_0183, OTT_0256, OTT_0290, OTT_0306, OTT_0314, OTT_0453, OTT_0530, OTT_0682, OTT_0888, OTT_1033, OTT_1464, OTT_1698, OTT_1731, OTT_1854, abcT1, abcT3, alaS, aprD, aspS, atpA, atpD, ccmA, clpX, cysS, dnaJ, dnaK, dnaX, folC, ftsH, gatA, gatB, glnQ, gltX1, gltX2, gmk, groL, groS, gyrB, hscA, htpG, ileS, leuS, lysS, metS, miaA, mkl, mrp, msbA1, msbA2, murD, murE, murF, mutS, ndk, nrdA, ntrX, pcnB, pgk, pheS, pheT, priA, proS, purC, recA, recD, rho, serS, tilS, tmk, trpS, uup, uvrA, uvrD, virB11, ychF, znuC] |
| 9 | GO:0017111 | nucleoside-triphosphatase activity | [Group18, Group20, Group21] | 48 | 66 | [OTT_0021, OTT_0166, OTT_0183, OTT_0256, OTT_0290, OTT_0306, OTT_0314, OTT_0453, OTT_0530, OTT_0888, OTT_1033, OTT_1464, OTT_1698, OTT_1731, OTT_1854, abcT1, abcT3, aprD, atpA, atpD, atpG, ccmA, era, ffh, ftsH, ftsZ, fusA, glnQ, gyrB, infB, lepA, mkl, mrp, msbA1, msbA2, obg, priA, recA, recD, rho, thdF, tuf, uup, uvrA, uvrD, ychF, znuC] |
| 10 | GO:0016462 | pyrophosphatase activity | [Group18, Group20, Group21] | 50 | 63 | [OTT_0021, OTT_0166, OTT_0183, OTT_0256, OTT_0290, OTT_0306, OTT_0314, OTT_0453, OTT_0530, OTT_0888, OTT_1033, OTT_1464, OTT_1698, OTT_1731, OTT_1854, abcT1, abcT3, aprD, atpA, atpD, atpG, ccmA, dut, era, ffh, ftsH, ftsZ, fusA, glnQ, gyrB, infB, lepA, mkl, mrp, msbA1, msbA2, obg, ppa, priA, recA, recD, rho, thdF, tuf, uup, uvrA, uvrD, ychF, znuC] |
| 11 | GO:0016818 | hydrolase activity, acting on acid anhydrides, in phosphorus-containing anhydrides | [Group18, Group20, Group21] | 50 | 62 | [OTT_0021, OTT_0166, OTT_0183, OTT_0256, OTT_0290, OTT_0306, OTT_0314, OTT_0453, OTT_0530, OTT_0888, OTT_1033, OTT_1464, OTT_1698, OTT_1731, OTT_1854, abcT1, abcT3, aprD, atpA, atpD, atpG, ccmA, dut, era, ffh, ftsH, ftsZ, fusA, glnQ, gyrB, infB, lepA, mkl, mrp, msbA1, msbA2, obg, ppa, priA, recA, recD, rho, thdF, tuf, uup, uvrA, uvrD, ychF, znuC] |
| 12 | GO:0046872 | metal ion binding | [Group01] | 58 | 60 | [OTT_0682, acpS, alaS, clpX, codA, coxA, ctaQ, cycM, cysS, dapE, def, dksA, dnaG, dnaJ, dut, engB, fbcH, ftsH, gltX2, grlA, gyrB, hemB, hemH, icd, ileS, iscU, ligA, lipA, mgtE, miaB, mrp, ndk, nth, nuoB, nuoE, nuoF, nuoG, obg, pheS, pheT, pnp, ppa, priA, recR, rnc, rnhB, rpoH, ruvC, sdhB, sodB, spl1, thdF, tme, topA, uppS, uvrA, ybeY] |
| 13 | GO:0003677 | DNA binding | [Group18] | 44 | 46 | [OTT_0021, OTT_0166, OTT_0179, OTT_0256, OTT_0290, OTT_0306, OTT_0314, OTT_0453, OTT_0888, OTT_1033, OTT_1223, OTT_1464, OTT_1698, OTT_1731, OTT_1854, cspA, czcR, dnaG, dnaN, dnaQ, dnaX, greA, gyrB, hupA, ligA, mutS, nth, ntrX, phoB, priA, recA, recD, recR, rpoD, rpoH, spo0J, ssb, topA, uup, uvrA, uvrD, xerC, xerD, xthA2] |
| 14 | GO:0016887 | ATPase activity | [Group20, Group21] | 25 | 35 | [OTT_0021, OTT_0530, abcT1, abcT3, aprD, atpA, atpD, atpG, ccmA, ftsH, glnQ, gyrB, mkl, mrp, msbA1, msbA2, priA, recA, recD, rho, uup, uvrA, uvrD, ychF, znuC] |
| 15 | GO:0003723 | RNA binding | [Group17] | 29 | 34 | [alaS, era, ffh, fusA, gltX1, gltX2, ileS, infA, infB, lysS, pcnB, pheS, pheT, pnp, rho, rluC, rnc, rne, rnhB, rph, rplB, rplK, rplP, rplX, rpsD, truB, tsf, tuf] |
| 16 | GO:0004386 | helicase activity | [Group18, Group20] | 18 | 26 | [OTT_0021, OTT_0166, OTT_0183, OTT_0256, OTT_0290, OTT_0306, OTT_0314, OTT_0453, OTT_0888, OTT_1033, OTT_1464, OTT_1698, OTT_1731, OTT_1854, priA, recD, rho, uvrD] |
| 17 | GO:0042623 | ATPase activity, coupled | [Group20, Group21] | 17 | 26 | [OTT_0021, OTT_0530, abcT3, aprD, atpA, atpD, atpG, ccmA, glnQ, gyrB, msbA1, msbA2, priA, recA, recD, rho, uvrD] |
| 18 | GO:0003678 | DNA helicase activity | [Group18, Group20] | 17 | 23 | [OTT_0021, OTT_0166, OTT_0183, OTT_0256, OTT_0290, OTT_0306, OTT_0314, OTT_0453, OTT_0888, OTT_1033, OTT_1464, OTT_1698, OTT_1731, OTT_1854, priA, recD, uvrD] |
| 19 | GO:0005525 | GTP binding | [Group19] | 13 | 22 | [der, engB, era, ffh, ftsZ, fusA, infB, lepA, obg, thdF, tuf, ychF] |
| 20 | GO:0032550 | purine ribonucleoside binding | [Group19] | 13 | 22 | [der, engB, era, ffh, ftsZ, fusA, infB, lepA, obg, thdF, tuf, ychF] |
| 21 | GO:0032561 | guanyl ribonucleotide binding | [Group19] | 13 | 22 | [der, engB, era, ffh, ftsZ, fusA, infB, lepA, obg, thdF, tuf, ychF] |
| 22 | GO:0004518 | nuclease activity | [Group15] | 15 | 21 | [OTT_0021, OTT_0179, OTT_0766, OTT_1223, dnaN, nth, pnp, recJ, rnc, rne, rnhB, ruvC, uvrA, xthA2, ybeY] |
| 23 | GO:0019001 | guanyl nucleotide binding | [Group19] | 13 | 21 | [der, engB, era, ffh, ftsZ, fusA, infB, lepA, obg, thdF, tuf, ychF] |
| 24 | GO:0001883 | purine nucleoside binding | [Group19] | 13 | 20 | [der, engB, era, ffh, ftsZ, fusA, infB, lepA, obg, thdF, tuf, ychF] |
| 25 | GO:0032549 | ribonucleoside binding | [Group19] | 13 | 20 | [der, engB, era, ffh, ftsZ, fusA, infB, lepA, obg, thdF, tuf, ychF] |
| 26 | GO:0015075 | ion transmembrane transporter activity | [Group21] | 12 | 20 | [OTT_1942, atpA, atpD, atpG, coxA, coxC, glnQ, kefB, mgtE, petB, yqiX, znuC] |
| 27 | GO:0042626 | ATPase activity, coupled to transmembrane movement of substances | [Group21] | 10 | 20 | [OTT_0530, abcT3, aprD, atpA, atpD, atpG, ccmA, glnQ, msbA1, msbA2] |
| 28 | GO:0015405 | P-P-bond-hydrolysis-driven transmembrane transporter activity | [Group21] | 10 | 19 | [OTT_0530, abcT3, aprD, atpA, atpD, atpG, ccmA, glnQ, msbA1, msbA2] |
| 29 | GO:0003924 | GTPase activity | [Group19] | 10 | 18 | [era, ffh, ftsZ, fusA, infB, lepA, obg, thdF, tuf] |
| 30 | GO:0008324 | cation transmembrane transporter activity | [Group21] | 10 | 18 | [OTT_1942, atpA, atpD, atpG, coxA, coxC, kefB, mgtE, petB, znuC] |
| 31 | GO:0015399 | primary active transmembrane transporter activity | [Group21] | 10 | 18 | [OTT_0530, abcT3, aprD, atpA, atpD, atpG, ccmA, glnQ, msbA1, msbA2] |
| 32 | GO:0016820 | hydrolase activity, acting on acid anhydrides, catalyzing transmembrane movement of substances | [Group21] | 10 | 18 | [OTT_0530, abcT3, aprD, atpA, atpD, atpG, ccmA, glnQ, msbA1, msbA2] |
| 33 | GO:0004812 | aminoacyl-tRNA ligase activity | [Group17] | 14 | 17 | [alaS, aspS, cysS, gltX1, gltX2, ileS, leuS, lysS, metS, pheS, pheT, proS, serS, trpS] |
| 34 | GO:0016876 | ligase activity, forming aminoacyl-tRNA and related compounds | [Group17] | 14 | 17 | [alaS, aspS, cysS, gltX1, gltX2, ileS, leuS, lysS, metS, pheS, pheT, proS, serS, trpS] |
| 35 | GO:0022890 | inorganic cation transmembrane transporter activity | [Group21] | 9 | 17 | [atpA, atpD, atpG, coxA, coxC, kefB, mgtE, petB, znuC] |
| 36 | GO:0022853 | active ion transmembrane transporter activity | [Group21] | 4 | 16 | [atpA, atpD, atpG, glnQ] |
| 37 | GO:0042625 | ATPase coupled ion transmembrane transporter activity | [Group21] | 4 | 16 | [atpA, atpD, atpG, glnQ] |
| 38 | GO:0046914 | transition metal ion binding | [Group12] | 12 | 15 | [clpX, codA, coxA, dksA, dnaG, ftsH, gltX2, ileS, iscU, priA, rpoH, ybeY] |
| 39 | GO:0015077 | monovalent inorganic cation transmembrane transporter activity | [Group21] | 7 | 15 | [atpA, atpD, atpG, coxA, coxC, kefB, petB] |
| 40 | GO:0015078 | hydrogen ion transmembrane transporter activity | [Group21] | 7 | 14 | [atpA, atpD, atpG, coxA, coxC, kefB, petB] |
| 41 | GO:0000049 | tRNA binding | [Group17] | 9 | 13 | [alaS, gltX1, gltX2, ileS, lysS, pheS, pheT, rph, rplP] |
| 42 | GO:0008270 | zinc ion binding | [Group12] | 10 | 12 | [clpX, codA, dksA, dnaG, ftsH, gltX2, ileS, priA, rpoH, ybeY] |
| 43 | GO:0004519 | endonuclease activity | [Group15] | 10 | 12 | [OTT_0179, OTT_0766, OTT_1223, nth, rnc, rnhB, ruvC, uvrA, xthA2, ybeY] |
| 45 | GO:0051539 | 4 iron, 4 sulfur cluster binding | [Group16] | 8 | 12 | [OTT_1002, lipA, miaB, nth, nuoB, nuoF, nuoG, sdhB] |
| 45 | GO:0050136 | NADH dehydrogenase (quinone) activity | [Group16] | 4 | 11 | [nuoB, nuoC, nuoF, nuoG] |
| 46 | GO:0019843 | rRNA binding | [Group17] | 8 | 10 | [era, infA, rnc, rplB, rplK, rplP, rplX, rpsD] |
| 47 | GO:0016779 | nucleotidyltransferase activity | [Group09] | 9 | 9 | [cdsA, dnaG, dnaN, dnaQ, dnaX, lipA, pcnB, pnp, rph] |
| 48 | GO:0003954 | NADH dehydrogenase activity | [Group16] | 4 | 9 | [nuoB, nuoC, nuoF, nuoG] |
| 49 | GO:0008137 | NADH dehydrogenase (ubiquinone) activity | [Group16] | 4 | 9 | [nuoB, nuoC, nuoF, nuoG] |
| 50 | GO:0016655 | oxidoreductase activity, acting on NAD(P)H, quinone or similar compound as acceptor | [Group16] | 4 | 9 | [nuoB, nuoC, nuoF, nuoG] |
| 51 | GO:0008135 | translation factor activity, RNA binding | [Group19] | 6 | 8 | [fusA, infA, infB, tsf, tuf] |
| 52 | GO:0000287 | magnesium ion binding | [Group00] | 6 | 7 | [acpS, obg, pheS, pheT, ppa, uppS] |
| 53 | GO:0016301 | kinase activity | [Group06] | 7 | 7 | [argB, gmk, lysC, ndk, pgk, pkcI, tmk] |
| 54 | GO:0030170 | pyridoxal phosphate binding | [Group14] | 5 | 7 | [OTT_1072, glyA, spl1, tdcB] |
| 55 | GO:0070279 | vitamin B6 binding | [Group14] | 5 | 7 | [OTT_1072, glyA, spl1, tdcB] |
| 56 | GO:0004540 | ribonuclease activity | [Group15] | 5 | 7 | [pnp, rnc, rne, rnhB, ybeY] |
| 57 | GO:0051537 | 2 iron, 2 sulfur cluster binding | [Group03] | 6 | 6 | [OTT_0568, grlA, nuoE, sdhB, spl1] |
| 58 | GO:0008237 | metallopeptidase activity | [Group13] | 4 | 6 | [OTT_1939, ctaQ, ftsH, ybeY] |
| 59 | GO:0070011 | peptidase activity, acting on L-amino acid peptides | [Group13] | 4 | 6 | [OTT_1939, ctaQ, ftsH, ybeY] |
| 60 | GO:0004527 | exonuclease activity | [Group15] | 4 | 6 | [OTT_0021, dnaN, pnp, recJ] |
| 61 | GO:0050660 | flavin adenine dinucleotide binding | [Group02] | 4 | 5 | [OTT_0625, mnmG, murB, tlyC] |
| 62 | GO:0070035 | purine NTP-dependent helicase activity | [Group04] | 4 | 5 | [OTT_0021, priA, recD, uvrD] |
| 63 | GO:0016881 | acid-amino acid ligase activity | [Group11] | 5 | 5 | [folC, murD, murE, murF, purC] |
| 64 | GO:0015036 | disulfide oxidoreductase activity | [Group05] | 4 | 4 | [grlA, grxC, trxA, trxB1] |
| 65 | GO:0016616 | oxidoreductase activity, acting on the CH-OH group of donors, NAD or NADP as acceptor | [Group07] | 4 | 4 | [icd, mdh, murB, tme] |
| 66 | GO:0016747 | transferase activity, transferring acyl groups other than amino-acyl groups | [Group08] | 4 | 4 | [OTT_1072, fabD, fabF, sucB] |
| 67 | GO:0016811 | hydrolase activity, acting on carbon-nitrogen (but not peptide) bonds, in linear amides | [Group10] | 4 | 4 | [OTT_1510, dapE, def, gatA] |

**Supplementary Table S3D. Description of putative virulent MBPs of *O. tsutsugamushi*.**

| **S.No.** | **Sequence ID** | **Uniprot ID** | **Gene** | **Metal bound** | **Subcellular location** | **Domain** | **Function** | **Broad Class** | **Toxin** | **DrugBank ID** |
| --- | --- | --- | --- | --- | --- | --- | --- | --- | --- | --- |
| 1 | WP_012460810.1 | B3CQP7 | *purC* | Mg | Cytoplasm | SAICAR synthetase | Purine biosynthesis pathway | Metabolism | Yes | DB00128 |
| 2 | WP_012460966.1 | B3CQ27 | *acpS* | Mg, Ca, Ni | Cytoplasm | Phosphopantetheine-protein transferase domain | Biosynthesis of fatty acid and siderophores | Metabolism | Yes | DB01992, DB04447 |
| 3 | WP_012461072.1 | NF | NF | Mg, Ca | Cytoplasm | Pyruvate, phosphate dikinase | Pyruvate metabolic process | Metabolism | Yes | DB02522, DB08357 |
| 4 | WP_012461235.1 | NF | NF | Zn | Cytoplasm | Citrate synthase | Energy generation and carbon assimilation | Metabolism | Yes | DB01992, DB04272 |
| 5 | WP_012461304.1 | B3CRE7 | *murD* | Mg, Mn, Ni | Cytoplasm | UDP-N-acetylmuramoylalanine--D-glutamate ligase [murD]. | Regulation of cell division and cell shape of bacterial cell wall | Metabolism | Yes | DB01673, DB02314, DB03801, DB08105, DB08106, DB08107, DB08108, DB08112 |
| 6 | WP_012461438.1 | B3CSI8 | *argB* | Mg, Ni | Cytoplasm | Acetylglutamate kinase | Arginine biosynthesis | Metabolism | Yes | DB04075 |
| 7 | WP_012461493.1 | B3CSG3 | *murA* | Mg, Ca | Cytoplasm | UDP-N-acetylglucosamine 1-carboxyvinyltransferase (murA) | UDP-N-acetylgalactosamine biosynthetic process | Metabolism | Yes | DB01879, DB02435, DB02995, DB04174, DB04474, DB03397, DB00828 |
| 8 | WP_012461532.1 | B3CSQ9 | *murB* | Mg, Ca | Cytoplasm | UDP-N-acetylenolpyruvoylglucosamine reductase (murB) | Biogenesis of cellwall peptidoglycan | Metabolism | Yes | DB03147, DB03147, DB07296 |
| 9 | WP_012461533.1 | NA | NA | Mg, Ca | Cytoplasm | D-alanine--D-alanine ligase, C-terminal | Biosynthesis of cellwall peptidoglycan | Metabolism | Yes | DB07805, DB00260 |
| 10 | WP_012461772.1 | B3CTL4 | *fdxA* | Fe | Cytoplasm | 7Fe ferredoxin | Electron transport | Metabolism | Yes | DB08689 |
| 11 | WP_012461775.1 | B3CTL7 | *glnA* | Mg, Mn | Cytoplasm | Glutamine synthetase, catalytic domain | Nitrogen compound metabolic process | Metabolism | Yes | DB02663 |
| 12 | WP_012462053.1 | B3CUK6 | *dcd* | Mg | Cytoplasm | Deoxycytidine triphosphate deaminase/ dUTPase-like | 2'-deoxyribonucleotide metabolic process | Metabolism | Yes | DB02333, DB03258, |
| 13 | WP_012460848.1 | B3CQ87 | *OTT_0126* | Mn | Cytoplasm | HD domain | Nucleotide metabolism and signal transduction | Cell signaling | Yes | DB02836, DB04315 |
| 14 | WP_012460866.1 | B3CS26 | *OTT_0148* | Mn | Cytoplasm | HD domain | Nucleotide metabolism and signal transduction | Cell signaling | Yes | DB02836, DB04315 |
| 15 | WP_012460872.1 | B3CS47 | *OTT_0154* | Mn | Cytoplasm | HD domain | Nucleotide metabolism and signal transduction | Cell signaling | Yes | DB02836, DB04315 |
| 16 | WP_012461036.1 | B3CQB3 | *OTT_0351* | Mn | Cytoplasm | HD domain | Nucleotide metabolism and signal transduction | Cell signaling | Yes | DB02836, DB04315 |
| 17 | WP_012461119.1 | NF | NF | Mn | Cytoplasm | HD domain | Nucleotide metabolism and signal transduction | Cell signaling | Yes | DB02836, DB04315 |
| 18 | WP_012461298.1 | B3CRD9 | *OTT_0665* | Mn | Cytoplasm | HD domain | Nucleotide metabolism and signal transduction | Cell signaling | Yes | DB02836, DB04315 |
| 19 | WP_012461371.1 | B3CRR8 | *OTT_0759* | Mn | Cytoplasm | HD domain | Nucleotide metabolism and signal transduction | Cell signaling | Yes | DB02836, DB04315 |
| 20 | WP_012461472.1 | NF | NF | Mn | Cytoplasm | HD domain | Nucleotide metabolism and signal transduction | Cell signaling | Yes | DB02836, DB04315 |
| 21 | WP_012461589.1 | B3CSY3 | *OTT_1022* | Mn | Cytoplasm | HD domain | Nucleotide metabolism and signal transduction | Cell signaling | Yes | DB02836, DB04315 |
| 22 | WP_012461744.1 | B3CTI2 | *OTT_1221* | Mn | Cytoplasm | HD domain | Nucleotide metabolism and signal transduction | Cell signaling | Yes | DB02836, DB04315 |
| 23 | WP_012461761.1 | NF | NF | Mn | Cytoplasm | HD domain | Nucleotide metabolism and signal transduction | Cell signaling | Yes | DB02836, DB04315 |
| 24 | WP_012461942.1 | B3CU58 | *OTT_1447* | Mn | Cytoplasm | HD domain | Nucleotide metabolism and signal transduction | Cell signaling | Yes | DB02836, DB04315 |
| 25 | WP_012462073.1 | B3CUN0 | *OTT_1619* | Mn | Cytoplasm | HD domain | Nucleotide metabolism and signal transduction | Cell signaling | Yes | DB02836, DB04315 |
| 26 | WP_012462097.1 | B3CUR0 | *OTT_1649* | Mn | Cytoplasm | HD domain | Nucleotide metabolism and signal transduction | Cell signaling | Yes | DB02836, DB04315 |
| 27 | WP_012462180.1 | B3CV23 | *OTT_1762* | Mn | Cytoplasm | HD domain | Nucleotide metabolism and signal transduction | Cell signaling | Yes | DB02836, DB04315 |
| 28 | WP_012462254.1 | B3CVA8 | *OTT_1847* | Mg | Cytoplasm | HD domain | Nucleotide metabolism and signal transduction | Cell signaling | Yes | DB02836, DB04315 |
| 29 | WP_012462260.1 | B3CVB4 | *OTT_1853* | Mn | Cytoplasm | HD domain | Nucleotide metabolism and signal transduction | Cell signaling | Yes | DB02836, DB04315 |
| 30 | WP_012462292.1 | NF | NF | Mn | Cytoplasm | HD domain | Nucleotide metabolism and signal transduction | Cell signaling | Yes | DB02836, DB04315 |
| 31 | WP_012462309.1 | B3CVJ1 | *OTT_1930* | Mn | Cytoplasm | HD domain | Nucleotide metabolism and signal transduction | Cell signaling | Yes | DB02836, DB04315 |
| 32 | WP_041621594.1 | NF | NF | Mn | Cytoplasm | HD domain | Nucleotide metabolism and signal transduction | Cell signaling | Yes | DB02836, DB04315 |
| 33 | WP_041621687.1 | NF | NF | Mg | Cytoplasm | Tetrahydrodipicolinate N-succinyltransferase, transferase hexapeptide repeat family | Lysine biosynthesis via diaminopimelate | Metabolism | Yes | DB01856, DB01992, DB03134, DB03699, DB03905 |
| 34 | WP_041621803.1 | NF | NF | Mg, Mn, Ni | Cytoplasm | UDP-N-acetylmuramate--L-alanine ligase (murC) | Biogenesis of cellwall peptidoglycan | Metabolism | Yes | DB01673, DB03909, DB04395, DB01673, DB02314, DB03801, DB08105, DB08106, DB08107, DB08108, DB08112 |
| 35 | WP_012461229.1 | B3CRJ6 | *OTT_0579* | Mg | InnerMembrane | Glycosyl transferase family group 2 | Biosynthesis of carbohydrates | Metabolism | Yes | No |
| 36 | WP_012461310.1 | B3CRW7 | *lysC* | Mg | Cytoplasm | Aspartate kinase domain | Biosynthesis of bacterial cellwall consituents peptidoglycan | Metabolism | Yes | No |
| 37 | WP_012461379.1 | B3CRS7 | *priA* | Zn | Cytoplasm | Prisomal protein N/DEAD/DEAH box helicase domain | Resumption of DNA replication | Gene expression and regulation | Yes | No |
| 38 | WP_012461393.1 | B3CRY3 | *OTT_0782* | Ca | InnerMembrane | Quinoprotein alcohol dehydrogenase-like domain | Oxidation of alcohol | Metabolism | Yes | No |
| 39 | WP_012461981.1 | B3CUC1 | *OTT_1510* | Zn | Cytoplasm | N-acetylmuramoyl-L-alanine amidase domain | Peptidoglycal catabolic process | Metabolism | Yes | No |
| 40 | WP_012462252.1 | B3CVA3 | *OTT_1842* | Ca | InnerMembrane | Glycoside hydrolase family 18, catalytic domain | Carbohydrate metabolic process | Metabolism | Yes | No |
| 41 | WP_012460897.1 | B3CQU4 | *OTT_0179* | Mg, Ca, Mn | Cytoplasm | AP endonuclease 1 | DNA Base-exision repair | Gene expression and regulation | Yes | DB04967 |
| 42 | WP_012460984.1 | B3CQ43 | *rpoD* | Mg | Cytoplasm | RNA polymerase sigma-70 | Regulate expression of prokaryotic transcription genes | Gene expression and regulation | Yes | DB08874 |
| 43 | WP_012461003.1 | B3CQ63 | *ntrX* | Mg | Cytoplasm | RNA polymerase sigma factor 54 interaction domain | Regulate expression of prokaryotic transcription genes | Gene expression and regulation | Yes | DB01857, DB09462, DB02355, DB02596, DB07706, DB02461, DB03487, DB04156 |
| 45 | WP_012461164.1 | B3CR61 | *Tmk* | Mg, Ca | Cytoplasm | Thymidylate kinase | DNA replication: Phosphorylation of dTMP to form dTDP | Gene expression and regulation | Yes | DB03280 |
| 45 | WP_012461168.1 | B3CR65 | *rho* | Mg, Ca | Cytoplasm | Rho termination factor, RNA-binding | Transcription termination | Gene expression and regulation | Yes | DB07384, DB07394, DB08399, DB04216, DB08629, DB08949, DB12695 |
| 46 | WP_012461332.1 | B3CRN4 | *OTT_0717* | Fe | Cytoplasm | Uracil-DNA glycosylase family 4 | Base excision repair | Gene expression and regulation | Yes | DB03419 |
| 47 | WP_012461360.1 | B3CRT8 | *dnaQ* | Ca, Mn | Cytoplasm | DNA polymerase 3, epsilon subunit | DNA replication, nucleotide and base excision repair | Gene expression and regulation | Yes | DB01643 |
| 48 | WP_012461492.1 | B3CSG2 | *ftsZ* | Mg, Ca | Cytoplasm | Tubulin/FtsZ family, GTPase domain | Bacterial cell division | Gene expression and regulation | Yes | DB01864, DB04272, DB04315, DB03532 |
| 49 | WP_012461603.1 | B3CT01 | *recA* | Mg, Ca | Cytoplasm | DNA recombination and repair protein RecA | DNA repair and recombination | Gene expression and regulation | Yes | DB02930, DB03222, DB04444, DB02930, DB03222, DB04395, DB02930, DB12742, DB04395 |
| 50 | WP_012461746.1 | B3CTI4 | *OTT_1223* | Mg, Ca, Mn | Cytoplasm | AP endonuclease 1 | DNA Base-exision repair | Gene expression and regulation | Yes | DB04967 |
| 51 | WP_012461765.1 | NF | NF | Ca | Cytoplasm | NusB antitermination factor | Transcription antitermination | Gene expression and regulation | Yes | DB04272 |
| 52 | WP_012461805.1 | B3CTQ7 | *rpoH* | Mg | Cytoplasm | RNA polymerase sigma-70 | Regulate expression of prokaryotic transcription genes | Gene expression and regulation | Yes | DB08874, DB08226, DB08266 |
| 53 | WP_012461831.1 | B3CTT6 | *topA* | Mg, Ca, Zn | Cytoplasm | DNA topoisomerase, type IA | DNA metabolism, topological change and chromosome condensation | Gene expression and regulation | Yes | DB00487, DB01051, DB01643, DB01812, DB04205 |
| 54 | WP_012461879.1 | NF | NF | Mg, Ca, Zn, Mn | Cytoplasm | DNA gyrase A | DNA metabolism, topological change and chromosome condensation | Gene expression and regulation | Yes | DB00537, DB11943, DB00218, DB00365, DB00467, DB00487, DB00537, DB00685, DB00978, DB01059, DB01137, DB01155, DB01165, DB01208, DB01405, DB00827, DB04576, DB06771, DB09047, DB12924, DB00537, DB01044, DB06771, DB00817, DB11943, DB12924, DB00218, DB00365, DB00467, DB00487, DB00537, DB00685, DB00978, DB01059, DB01137, DB01155, DB01165, DB01208, DB01405, DB04576, DB06771, DB09047, DB04395, DB05022, DB05488, DB06042, DB06362, DB06421, DB00694, DB08651, DB00380, DB00773, DB00970, DB00276, DB00380, DB00385, DB00444, DB00773, DB00997, DB01177, DB01204, DB00445, DB01179, DB01645, DB00537, DB00467, DB04576, DB0113, DB00978, DB00218, DB01059, DB00487, DB01208, DB00685, DB01165, DB05129, DB04975, DB04978, DB04967, DB05022, DB06042, DB05920, DB05706, DB06263, DB06362, DB06420, DB00694, DB06421, DB09047, DB06013, DB00970 |
| 55 | WP_012462032.1 | B3CUI3 | *dnaN* | Ca | Cytoplasm | DNA polymerase III, beta chain | DNA replication | Gene expression and regulation | Yes | DB06998 |
| 56 | WP_012462162.1 | B3CV01 | *dnaX* | Mg, Zn | Cytoplasm | DNA polymerase III, subunit gamma/ tau | DNA replication | Gene expression and regulation | Yes | DB02836, DB04315 |
| 57 | WP_011944565.1 | B3CSR8 | *rppH* | Mg | Cytoplasm | RNA pyrophosphohydrolase RppH | 5'-end-dependent mRNA decay | Gene expression and regulation | Yes | No |
| 58 | WP_012460745.1 | B3CQE0 | *OTT_0021* | Mg | Cytoplasm | UvrD-like DNA helicase | DNA repair, replication and recombination | Gene expression and regulation | Yes | No |
| 59 | WP_012460873.1 | NF | NF | Mg, Ca | Cytoplasm | DnaB-like helicase | DNA replication: Separation of DNA duplexes into single strands in an ATP-dependent manner | Gene expression and regulation | Yes | No |
| 60 | WP_012460901.1 | B3CS37 | *OTT_0183* | Mg, Ca | Cytoplasm | DnaB-like helicase | DNA replication: Separation of DNA duplexes into single strands in an ATP-dependent manner | Gene expression and regulation | Yes | No |
| 61 | WP_012460958.1 | NF | NF | Mg, Ca | Cytoplasm | DnaB-like helicase | DNA replication: Separation of DNA duplexes into single strands in an ATP-dependent manner | Gene expression and regulation | Yes | No |
| 62 | WP_012460983.1 | B3CQ42 | *dnaG* | Zn | Cytoplasm | Zinc finger, CHC2-type, DNA primase, DnaG | DNA replication: RNA primer synthesis | Gene expression and regulation | Yes | No |
| 63 | WP_012460994.1 | B3CQ55 | *rne* | Mg, Zn | Cytoplasm | Ribonuclease E/G family | mRNA degradation | Gene expression and regulation | Yes | No |
| 64 | WP_012461008.1 | B3CQ68 | *OTT_0306* | Ca | Cytoplasm | DnaB-like helicase | DNA replication: Separation of DNA duplexes into single strands in an ATP-dependent manner | Gene expression and regulation | Yes | No |
| 65 | WP_012461098.1 | B3CQX5 | *spo0J* | Co | Cytoplasm | ParB-like nuclease domain | Chromosome partition | Gene expression and regulation | Yes | No |
| 66 | WP_012461508.1 | B3CSI0 | *ruvC* | Mg | Cytoplasm | Crossover junction endodeoxyribonuclease RuvC | DNA recombination and repair | Gene expression and regulation | Yes | No |
| 67 | WP_012461635.1 | B3CT36 | *cinA* | Mg | OuterMembrane | Competence-damaged protein, CinA | Genetic recombination | Gene expression and regulation | Yes | No |
| 68 | WP_012461640.1 | B3CT42 | *ybeY* | Ni | Cytoplasm | Endoribonuclease YbeY | rRNA processing | Gene expression and regulation | Yes | No |
| 69 | WP_012461806.1 | B3CTQ8 | *OTT_1297* | Cu | InnerMembrane | Copper chaperone PCu(A)C | Biogenesis and assembly of respiratory complex | Protein folding | Yes | No |
| 70 | WP_012462272.1 | B3CVC6 | *dksA* | Zn | Cytoplasm | Zinc finger, DksA/TraR C4-type | Regulation of rRNA promoters and virulence factor formation | Gene expression and regulation | Yes | No |
| 71 | WP_012462273.1 | B3CVC7 | *xerC* | Mg, Zn | Cytoplasm | Integrase/recombinase | Site specific recombination | Gene expression and regulation | Yes | No |
| 72 | WP_012462325.1 | NF | NF | Mg, Ca, Cu | Cytoplasm | DnaB-like helicase | DNA replication: Separation of DNA duplexes into single strands in an ATP-dependent manner | Gene expression and regulation | Yes | No |
| 73 | WP_012460740.1 | B3CQD5 | *rpmF* | Zn | Cytoplasm | Zinc-binding ribosomal protein | Translation and response to stress | Gene expression and regulation | Yes | DB13179 |
| 74 | WP_012461247.1 | B3CRL4 | *pheT* | Mg, Mn | Cytoplasm | Phenylalanine-tRNA ligase, class IIc, beta subunit | Phenyalanyl-tRNA aminoacylation | Gene expression and regulation | Yes | DB07817, DB00120 |
| 75 | WP_012460749.1 | B3CQE4 | *hupA* | Mn, Ni, Cd | Cytoplasm | Integration host factor (IHF)-like DNA-binding domain | Modulation of chromosomal conformation | Gene expression and regulation | Yes | No |
| 76 | WP_012461804.1 | B3CTQ6 | *greA* | Zn | Cytoplasm | Transcription elongation factor, GreA/GreB, N-terminal | Induce nucleolytic activity of RNAP and response to stress | Gene expression and regulation | Yes | No |
| 77 | WP_012461876.1 | B3CTY7 | *infA* | Zn | Cytoplasm | Translation initiation factor IF-1 | Translational initiation | Gene expression and regulation | Yes | No |
| 78 | WP_012461055.1 | B3CQR2 | *czcR* | Mg | Cytoplasm | OmpR/PhoB-type DNA-binding domain | Stress sensor response regulator | Cell signaling | Yes | DB02355, DB02596, DB07706, DB01972, DB01857 |
| 79 | WP_012461643.1 | B3CT45 | *OTT_1084* | Mg, Ca, Zn, Mn | Cytoplasm | Diguanylate cyclase, GGDEF domain | Biosynthesis of c-di-GMP | Cell signaling | Yes | DB01972, DB02355, DB02596, DB07706, DB01857, DB02461, DB03487, DB04156 |
| 80 | WP_012461406.1 | B3CRZ6 | *phoB* | Mg, Ca | Cytoplasm | OmpR/PhoB-type DNA-binding domain | Stress sensor response regulator | Cell signaling | Yes | No |
| 81 | WP_012462223.1 | B3CV68 | *OTT_1807* | Mg, Ca, Mn | InnerMembrane | EAL domain | Hydrolysis of c-di-GMP | Cell signaling | Yes | No |
| 82 | WP_041621607.1 | NF | NF | Zn | InnerMembrane | Phospholipase D/Transphosphatidylase | secondary messenger formation, host invasion and colonization | Cell signaling | Yes | No |
| 83 | WP_041621579.1 | B3CVM6 | *msbA2* | Mg | InnerMembrane | Type-IV secretion system protein TraC/Conjugative transfer ATPase | Conjugal transfer, synthesis and assembly of F conjugative pilus | Transport | Yes | No |
| 84 | WP_012461905.1 | B3CU18 | *uvrA* | Mg, Zn, Fe | Cytoplasm | UvrABC system subunit A | DNA repair | Gene expression and regulation | Yes | DB00997, DB00171, DB00778, DB04881, DB04905, DB05449, DB06240, DB06191, DB00661, DB01232, DB00220 |
| 85 | WP_012460742.1 | B3CQD7 | *yqiX* | Zn | Periplasm | Bacterial solute-binding proteins, family 3 | Transport of solute ions and virulence | Transport | Yes | No |
| 86 | WP_012460950.1 | B3CSA8 | *mgtE* | Mg, Ca, Mn | InnerMembrane | Magnesium transporter, MgtE intracellular domain | Metal ion transport | Transport | Yes | No |
| 87 | WP_012461355.1 | B3CRT2 | *tolQ* | Ca | InnerMembrane | MotA/TolQ/ExbB proton channel family | Ion potential-driven molecular motors | Transport | Yes | No |
| 88 | WP_012462319.1 | B3CVK3 | *OTT_1942* | Zn | InnerMembrane | Cation efflux protein | Zinc efflux | Transport | Yes | No |
| 89 | WP_012462008.1 | B3CUF4 | *trxB1* | Mg, Ca, Fe, Cd | Cytoplasm | Thioredoxin reductase | Response to superoxide radical | Stress response regulator | Yes | DB03147, DB00548, DB03147, DB03147, DB00262, DB00157, DB00143, DB01644, DB02153, DB02553, DB02895, DB03147, DB03310, DB03867, DB07393, DB07714, DB09110, DB11135, DB09130, DB09061 |
| 90 | WP_012461884.1 | B3CTZ6 | *rph* | Cd | Cytoplasm | Ribonuclease PH, bacterial-type | tRNA processing | Gene expression and regulation | Yes | DB03309 |
| 91 | WP_012462041.1 | B3CUJ2 | *OTT_1581* | Mg | Cytoplasm | tRNA threonylcarbamoyl adenosine modification protein TsaE | tRNA threonylcarbamoyladenosine modification | Post translational modification | Yes | No |
| 92 | WP_012462044.1 | B3CUJ5 | *tilS* | Mg | Cytoplasm | tRNA(Ile)-lysidine synthase, N-terminal | tRNA modification | Gene expression and regulation | Yes | No |
| 93 | WP_012460954.1 | B3CQ14 | *gppA* | Ca | InnerMembrane | Ppx/GppA phosphatase family | Stress response regulator and virulence | Stress response regulator | Yes | DB03382 |
| 94 | WP_012461444.1 | B3CSJ4 | *cutA* | Cu | Cytoplasm | Divalent ion tolerance protein, CutA | Response to ion tolerance | Stress response regulator | Yes | DB03975 |
| 95 | WP_012462166.1 | B3CV05 | *phnP* | Zn, Mn | Cytoplasm | Metallo-beta-lactamase | Catalyse the hydrolysis of almost all beta-lactam antibacterials | Antimicrobial resistance | Yes | No |
| 96 | WP_050731342.1 | NF | NF | Ca, Zn, Mn | Cytoplasm | Metallo-beta-lactamase | Catalyse the hydrolysis of almost all beta-lactam antibacterials | Antimicrobial resistance | Yes | No |
| 97 | WP_012461099.1 | B3CQX6 | *soj* | Mg | InnerMembrane | AAA domain/ParA | Chromosome partition | Gene expression and regulation | Yes | No |
| 98 | WP_012460964.1 | B3CQ25 | *groS* | Ca | Cytoplasm | Chaperonin 10 | Heat stress response and protien folding | Protein folding | Yes | DB12695 |

**NF: Not Found**

**SupplymentaryTable S3E. Physiochemical properties of putative virulent MBPs of *O. tsutsugamushi*.**

| **S.No.** | **Sequence ID** | **Number of amino acids** | **Molecular weight** | **Theoretical pI** | **Total number of negatively charged residues (Asp + Glu)** | **Total number of positively charged residues (Arg + Lys)** | **Instability index** | **Aliphatic index** | **Grand average of hydropathicity (GRAVY)** |
| --- | --- | --- | --- | --- | --- | --- | --- | --- | --- |
| 1 | WP_011944565.1 | 161 | 19255.47 | 9.66 | 14 | 24 | 41.17 | 87.83 | -0.453 |
| 2 | WP_012460740.1 | 68 | 7649.61 | 10.44 | 4 | 12 | 21.66 | 55.88 | -0.999 |
| 3 | WP_012460742.1 | 267 | 30068.09 | 8.98 | 32 | 37 | 23.03 | 117.19 | 0.025 |
| 4 | WP_012460745.1 | 1155 | 133456.08 | 5.84 | 140 | 119 | 45.6 | 105.39 | -0.116 |
| 5 | WP_012460749.1 | 102 | 11415.01 | 9.3 | 11 | 15 | 29.38 | 79.41 | -0.5 |
| 6 | WP_012460810.1 | 244 | 28417.09 | 5.99 | 30 | 28 | 24.18 | 111.43 | 0.031 |
| 7 | WP_012460848.1 | 195 | 22736.73 | 8.67 | 27 | 30 | 44.48 | 111.54 | -0.245 |
| 8 | WP_012460866.1 | 195 | 22860.92 | 8.86 | 26 | 30 | 53.66 | 111.54 | -0.217 |
| 9 | WP_012460872.1 | 183 | 21554.86 | 8.49 | 26 | 28 | 42.48 | 96.5 | -0.427 |
| 10 | WP_012460873.1 | 110 | 12707.24 | 5.35 | 18 | 14 | 53.68 | 76.18 | -0.755 |
| 11 | WP_012460897.1 | 261 | 30613.28 | 8.78 | 30 | 34 | 49.68 | 99.69 | -0.278 |
| 12 | WP_012460901.1 | 93 | 10866.08 | 4.46 | 20 | 11 | 45.28 | 79.57 | -0.724 |
| 13 | WP_012460950.1 | 455 | 51434.05 | 5.57 | 44 | 34 | 34.29 | 112.09 | 0.291 |
| 14 | WP_012460954.1 | 487 | 55604.1 | 8.98 | 43 | 53 | 43.61 | 115.69 | 0.158 |
| 15 | WP_012460958.1 | 94 | 10987.44 | 6.27 | 15 | 14 | 53.76 | 87.13 | -0.714 |
| 16 | WP_012460964.1 | 94 | 10541.25 | 6.73 | 15 | 15 | 10.54 | 100.43 | -0.383 |
| 17 | WP_012460966.1 | 131 | 14567.11 | 9.49 | 10 | 15 | 36.67 | 128.85 | 0.224 |
| 18 | WP_012460983.1 | 591 | 68037.9 | 6.39 | 72 | 68 | 33.81 | 97.19 | -0.285 |
| 19 | WP_012460984.1 | 615 | 70852.2 | 6.42 | 98 | 94 | 48.03 | 94.15 | -0.509 |
| 20 | WP_012460994.1 | 675 | 75884.39 | 8.84 | 79 | 88 | 54.08 | 93.93 | -0.461 |
| 21 | WP_012461003.1 | 489 | 54825.42 | 9.03 | 56 | 65 | 48.77 | 104.13 | -0.201 |
| 22 | WP_012461008.1 | 143 | 15892.49 | 5.36 | 15 | 12 | 30.73 | 126.92 | 0.268 |
| 23 | WP_012461036.1 | 192 | 22832.27 | 6 | 31 | 28 | 36.51 | 99.53 | -0.415 |
| 24 | WP_012461055.1 | 245 | 27618.89 | 6.01 | 35 | 32 | 37.64 | 111.84 | -0.15 |
| 25 | WP_012461072.1 | 901 | 100472.82 | 6.74 | 92 | 88 | 36.5 | 101.24 | -0.078 |
| 26 | WP_012461098.1 | 293 | 32937.87 | 9.32 | 32 | 38 | 48.39 | 114.44 | -0.291 |
| 27 | WP_012461099.1 | 265 | 29357.27 | 7.7 | 24 | 25 | 41.81 | 118.83 | 0.116 |
| 28 | WP_012461119.1 | 130 | 14963.4 | 7.77 | 17 | 18 | 35.11 | 97.54 | -0.28 |
| 29 | WP_012461164.1 | 217 | 25171.08 | 6.13 | 29 | 27 | 41.35 | 104.65 | -0.256 |
| 30 | WP_012461168.1 | 501 | 55544.77 | 7.19 | 68 | 68 | 42.68 | 98.64 | -0.28 |
| 31 | WP_012461229.1 | 583 | 68388.55 | 8.4 | 54 | 60 | 43.42 | 115.87 | 0.201 |
| 32 | WP_012461235.1 | 103 | 11670.28 | 4.71 | 12 | 7 | 44.06 | 94.66 | -0.082 |
| 33 | WP_012461247.1 | 103 | 11670.28 | 4.71 | 12 | 7 | 44.06 | 94.66 | -0.082 |
| 34 | WP_012461298.1 | 191 | 22557.02 | 6.92 | 28 | 28 | 39.82 | 95.97 | -0.443 |
| 35 | WP_012461304.1 | 472 | 52853.25 | 8.67 | 43 | 49 | 41.08 | 96.78 | -0.218 |
| 36 | WP_012461310.1 | 404 | 44468.86 | 7.15 | 32 | 32 | 30.36 | 117.55 | 0.292 |
| 37 | WP_012461332.1 | 268 | 30088.89 | 9.23 | 19 | 28 | 40.67 | 98.28 | -0.071 |
| 38 | WP_012461355.1 | 229 | 25555.79 | 8.89 | 15 | 19 | 35.86 | 117.16 | 0.292 |
| 39 | WP_012461360.1 | 222 | 25759.69 | 7.17 | 31 | 31 | 29.47 | 97.93 | -0.348 |
| 40 | WP_012461371.1 | 191 | 22735.31 | 8.95 | 26 | 30 | 39.12 | 98.01 | -0.455 |
| 41 | WP_012461379.1 | 646 | 73619.68 | 9.39 | 57 | 81 | 46.6 | 103.1 | -0.105 |
| 42 | WP_012461393.1 | 450 | 49918.57 | 9.52 | 33 | 49 | 37.45 | 106.78 | -0.106 |
| 43 | WP_012461406.1 | 249 | 28641.57 | 9.41 | 27 | 35 | 53.43 | 124.82 | -0.047 |
| 45 | WP_012461438.1 | 313 | 34410.48 | 5.19 | 30 | 24 | 40.97 | 114.6 | 0.081 |
| 45 | WP_012461444.1 | 118 | 13589.51 | 8.42 | 12 | 14 | 44.8 | 95.93 | -0.464 |
| 46 | WP_012461472.1 | 130 | 15017.39 | 6.96 | 18 | 18 | 37.84 | 97.54 | -0.331 |
| 47 | WP_012461492.1 | 450 | 48120.49 | 4.87 | 61 | 44 | 38.99 | 92.4 | -0.164 |
| 48 | WP_012461493.1 | 421 | 45342.11 | 9.09 | 31 | 41 | 42.77 | 115.89 | 0.282 |
| 49 | WP_012461508.1 | 159 | 17391.47 | 9.52 | 12 | 18 | 33.79 | 116.48 | 0.264 |
| 50 | WP_012461532.1 | 303 | 33618.58 | 9.46 | 23 | 35 | 16.68 | 97.46 | -0.165 |
| 51 | WP_012461533.1 | 324 | 36252.96 | 6.6 | 34 | 33 | 36.28 | 103.12 | -0.013 |
| 52 | WP_012461589.1 | 191 | 22649.06 | 5.88 | 30 | 27 | 40.35 | 98.53 | -0.413 |
| 53 | WP_012461603.1 | 356 | 38739.62 | 6.68 | 43 | 42 | 32.93 | 102.72 | -0.135 |
| 54 | WP_012461635.1 | 166 | 17966.62 | 7.56 | 13 | 14 | 41.7 | 105.78 | 0.131 |
| 55 | WP_012461640.1 | 167 | 19476.46 | 6.58 | 17 | 15 | 33.01 | 110.42 | -0.14 |
| 56 | WP_012461643.1 | 489 | 55932.2 | 5.02 | 75 | 59 | 36.48 | 111.9 | -0.181 |
| 57 | WP_012461744.1 | 191 | 22754.31 | 8.44 | 28 | 30 | 44.05 | 98.53 | -0.431 |
| 58 | WP_012461746.1 | 261 | 30672.46 | 9.07 | 28 | 34 | 49.82 | 98.93 | -0.236 |
| 59 | WP_012461761.1 | 95 | 11061.5 | 7.97 | 13 | 14 | 22.63 | 75.05 | -0.619 |
| 60 | WP_012461765.1 | 162 | 18726.6 | 4.75 | 21 | 15 | 25.59 | 113.7 | 0.14 |
| 61 | WP_012461772.1 | 106 | 12111.12 | 4.8 | 20 | 13 | 39.98 | 76.23 | -0.241 |
| 62 | WP_012461775.1 | 276 | 32173.98 | 8.13 | 27 | 29 | 46.3 | 98.26 | -0.303 |
| 63 | WP_012461804.1 | 159 | 18104.87 | 6.18 | 25 | 24 | 18.77 | 106.73 | -0.27 |
| 64 | WP_012461805.1 | 299 | 34242.18 | 7.83 | 36 | 37 | 36.84 | 104.41 | -0.371 |
| 65 | WP_012461806.1 | 180 | 19988.88 | 8.38 | 13 | 15 | 46.19 | 91 | -0.164 |
| 66 | WP_012461831.1 | 788 | 89601.02 | 9 | 93 | 109 | 44.73 | 92.22 | -0.397 |
| 67 | WP_012461876.1 | 74 | 8493.1 | 9.7 | 9 | 14 | 24.29 | 100 | -0.288 |
| 68 | WP_012461879.1 | 905 | 101251.05 | 6.35 | 120 | 115 | 37.89 | 99.07 | -0.263 |
| 69 | WP_012461884.1 | 251 | 27783.42 | 8.86 | 27 | 33 | 37.48 | 104.9 | -0.022 |
| 70 | WP_012461905.1 | 957 | 105999.86 | 6.62 | 111 | 107 | 38.54 | 96.14 | -0.222 |
| 71 | WP_012461942.1 | 191 | 22759.99 | 5.57 | 31 | 25 | 50.41 | 93.46 | -0.502 |
| 72 | WP_012461981.1 | 365 | 41538.81 | 6.4 | 36 | 31 | 35.45 | 86.3 | -0.45 |
| 73 | WP_012462008.1 | 320 | 34934.82 | 8.52 | 29 | 32 | 31.46 | 86.22 | -0.149 |
| 74 | WP_012462032.1 | 432 | 49192.58 | 5.92 | 54 | 47 | 35.5 | 104.72 | -0.196 |
| 75 | WP_012462041.1 | 140 | 15892.48 | 6.09 | 14 | 13 | 40.39 | 113.57 | 0.199 |
| 76 | WP_012462044.1 | 441 | 51538.66 | 9.28 | 39 | 54 | 36.72 | 100.98 | -0.181 |
| 77 | WP_012462053.1 | 188 | 21138.36 | 6.81 | 19 | 19 | 40.79 | 83.51 | -0.164 |
| 78 | WP_012462073.1 | 247 | 28576.23 | 8.88 | 28 | 34 | 41 | 99.11 | -0.299 |
| 79 | WP_012462097.1 | 204 | 23863.83 | 9.09 | 24 | 31 | 37.16 | 105.64 | -0.307 |
| 80 | WP_012462162.1 | 559 | 62320.22 | 6.63 | 59 | 58 | 31.59 | 108.84 | 0.052 |
| 81 | WP_012462166.1 | 249 | 28579.02 | 8.08 | 23 | 25 | 37.88 | 100.6 | -0.073 |
| 82 | WP_012462180.1 | 195 | 22765.83 | 8.58 | 27 | 30 | 41.89 | 109.54 | -0.196 |
| 83 | WP_012462223.1 | 410 | 47052.04 | 8.28 | 43 | 46 | 36.6 | 104.17 | 0.149 |
| 84 | WP_012462252.1 | 372 | 43319.95 | 8.08 | 38 | 40 | 38.15 | 97.26 | -0.202 |
| 85 | WP_012462254.1 | 197 | 23280.89 | 8.85 | 25 | 29 | 41.48 | 90.56 | -0.451 |
| 86 | WP_012462260.1 | 191 | 22596.07 | 8.47 | 28 | 30 | 37.25 | 93.98 | -0.451 |
| 87 | WP_012462272.1 | 143 | 16995.19 | 5.38 | 32 | 26 | 62.21 | 75.8 | -1.165 |
| 88 | WP_012462273.1 | 312 | 35848.41 | 9.78 | 21 | 42 | 41.6 | 104.07 | -0.309 |
| 89 | WP_012462292.1 | 135 | 15984.35 | 5.59 | 21 | 17 | 49.87 | 96.81 | -0.455 |
| 90 | WP_012462309.1 | 191 | 22749.14 | 6 | 31 | 28 | 36.65 | 97.49 | -0.453 |
| 91 | WP_012462319.1 | 305 | 34733.84 | 7.18 | 26 | 26 | 30.62 | 118.23 | 0.39 |
| 92 | WP_012462325.1 | 123 | 14573.66 | 9.65 | 14 | 23 | 46.91 | 70.49 | -0.891 |
| 93 | WP_041621579.1 | 658 | 73189.14 | 7.58 | 72 | 73 | 47.36 | 92.8 | -0.12 |
| 94 | WP_041621594.1 | 191 | 22671.14 | 6.98 | 28 | 28 | 42.53 | 96.54 | -0.409 |
| 95 | WP_041621607.1 | 196 | 21407.7 | 9.54 | 17 | 26 | 32.91 | 102.55 | -0.063 |
| 96 | WP_041621687.1 | 283 | 30983.59 | 8.51 | 28 | 32 | 32.46 | 96.04 | -0.071 |
| 97 | WP_041621803.1 | 490 | 54422.66 | 6.99 | 45 | 44 | 34.79 | 110.55 | 0.09 |
| 98 | WP_050731342.1 | 578 | 64373.35 | 8.38 | 59 | 63 | 39.14 | 102.09 | -0.149 |
